# The Deubiquitinase UCHL1 Drives Susceptibility to Atrial Fibrillation by Stabilizing CaMKII‐δ

**DOI:** 10.1002/advs.77855

**Published:** 2026-09-29

**Authors:** Hai‐Lian Bi, Yu‐Hui Gu, Kai‐Wen Ren, Xiao‐Hong Yu, Yun‐Long Xia, Xiao‐Lei Yang, Hui‐Hua Li

**Affiliations:** ^1^ Institute of Cardiovascular Diseases First Affiliated Hospital of Dalian Medical University Dalian China; ^2^ Department of Cardiology First Affiliated Hospital of Dalian Medical University Dalian China; ^3^ Institute of Biological Therapy Henan Engineering Research Center for Biotherapy of Metabolic Diseases The 7th People's Hospital of Zhengzhou Henan Academy of Innovations in Medical Science Zhengzhou China

**Keywords:** atrial fibrillation, CaMKII‐δ, Ca^2+^ mishandling, deubiquitinating enzymes, UCHL1

## Abstract

Atrial fibrillation (AF) is a common arrhythmia associated with calcium (Ca^2+^) dysregulation. Ca^2+^/calmodulin‐dependent protein kinase II‐δ (CaMKII‐δ) links Ca^2+^ handling to arrhythmogenesis, but whether deubiquitination regulates its stability in AF remains unclear. We established an AF model by infusing angiotensin II (Ang II) for 3 weeks in atrial cardiomyocyte‐specific ubiquitin C‐terminal hydrolase L1 (UCHL1)‐knockout (CKO) and recombinant adeno‐associated virus serotype 9 (rAAV9)‐UCHL1‐overexpressing mice. UCHL1 was markedly upregulated in atria from Ang II‐infused mice and patients with AF. UCHL1‐CKO attenuated Ang II‐induced atrial remodeling and AF susceptibility, reduced CaMKII‐δ stability, and improved Ca^2+^ handling, whereas UCHL1 overexpression produced the opposite effects. Mechanistically, UCHL1 bound to and deubiquitinated CaMKII‐δ at K251, preventing proteasomal degradation and thereby enhancing ryanodine receptor type 2 (RyR2) phosphorylation and Ca^2+^ mishandling. The ubiquitination‐deficient CaMKII‐δ‐K251R mutant aggravated AF, whereas CaMKII‐δ knockdown or inhibition reduced the proarrhythmic effects of UCHL1 overexpression. Furthermore, the UCHL1 inhibitor 6RK73 reduced AF inducibility in mice. These findings identify UCHL1‐mediated stabilization of the CaMKII‐δ–RyR2 axis as a mechanism of AF and support UCHL1 as a potential therapeutic target.

## Graphical abstract

UCHL1 is highly upregulated in mice and patients with AF. UCHL1 directly binds to and removes K48‐linked polyubiquitin chains from CaMKII‐δ at K251 and enhances CaMKII‐δ stability, which activates RyR2, disrupts Ca^2+^ handling, and promotes AF. These findings establish UCHL1 as a critical regulator of CaMKII‐δ stability and AF‐related Ca^2+^ handling. Targeting the UCHL1‐CaMKII‐δ pathway may offer a therapeutic approach for AF.

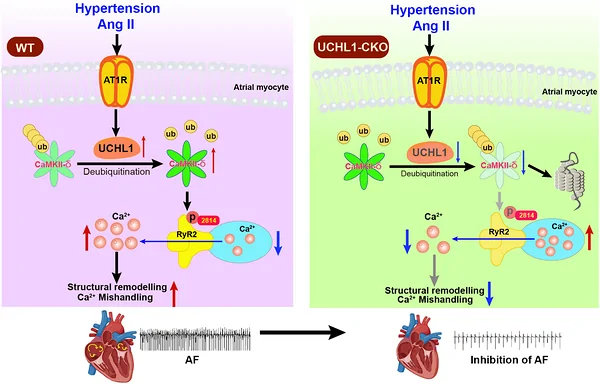

## 1 Introduction

Atrial fibrillation (AF) is the most common cardiac arrhythmia and is strongly associated with increased risks of stroke, heart failure (HF), and death [1]. Despite its high prevalence, therapeutic strategies remain suboptimal, and AF recurrence remains a major challenge. Pathological and electrophysiological studies have shown that atrial structural and electrical remodeling are central to the initiation and progression of AF [2]. Atrial arrhythmogenic remodeling is mainly characterized by atrial dilation, fibrosis, extracellular matrix deposition, inflammation, and conduction dysfunction [2]. Intracellular calcium (Ca^2+^) handling machinery, including Ca^2+^ cycling, activation of Ca^2+^/calmodulin‐dependent protein kinase II (CaMKII), and phosphorylation of ryanodine receptor type 2 (RyR2), is a critical contributor to atrial remodeling and AF development [3, 4]. CaMKII is a serine/threonine protein kinase that regulates inflammation, oxidative stress, matrix remodeling, and Ca^2+^ homeostasis in several cardiac disorders, including cardiac hypertrophy, HF, myocardial infarction (MI), ischemia/reperfusion injury (I/RI), and arrhythmias [5, 6, 7]. CaMKII‐δ is the predominant cardiac isoform and is highly expressed in atrial and ventricular myocytes from animal models and patients with persistent or postoperative AF [8, 9]. Inhibition of CaMKII activity improves intracellular Ca^2+^ handling and protects against AF in animal models and clinical settings [4]. To date, most studies have focused on CaMKII inhibition and post‐translational modifications (PTMs) of CaMKII, including oxidation, phosphorylation, S‐nitrosylation, O‐GlcNAcylation [10], and ubiquitination [11, 12]; however, the role of deubiquitination in regulating CaMKII stability remains understudied.

Ubiquitination and deubiquitination are reversible PTMs that govern intracellular protein stability and activity through a tightly regulated cascade involving E1, E2, and E3 enzymes and deubiquitinases (DUBs). DUBs are proteases that remove ubiquitin (Ub) from modified protein substrates; their dysregulation is linked to diverse cellular dysfunctions and human diseases. Accordingly, they have attracted increasing attention as therapeutic targets [13, 14]. Ubiquitin C‐terminal hydrolase L1 (UCHL1; also referred to as Parkin 5 or neuron‐specific protein PGP9.5) is a 27‐kDa protein composed of 223 amino acids and functions as a key DUB that regulates cell growth, apoptosis, and protein stability. UCHL1 is well established in neurodegenerative disorders, in which it modulates target‐protein ubiquitination [15]. Notably, it has been implicated in cardiovascular disease, including cardiac hypertrophy, vascular remodeling, MI, I/RI, hypertension, atherosclerosis, and pulmonary arterial hypertension (PAH), via different mechanisms [15, 16, 17, 18, 19, 20, 21, 22, 23]. Recent studies have begun to reveal the involvement of DUBs such as ubiquitin‐specific protease 7 (USP7) and USP38 in AF pathogenesis. USP7 was identified as a critical driver of AF, and its pharmacological inhibition alleviated angiotensin II (Ang II)‐induced AF [24]. Similarly, USP38 was shown to promote pressure overload‐ and chronic kidney disease (CKD)‐associated AF via NF‐κB/NLRP3‐mediated inflammation and STRAP‐dependent TGF‐β/SMAD signalling [25, 26]. However, none of these DUBs have been implicated in regulating CaMKII‐δ stability and Ca^2+^ homeostasis during AF development.

Here, we aimed to systematically explore the effects of atrial‐derived UCHL1 on Ang II‐induced atrial remodeling and AF susceptibility using atrial cardiomyocyte‐specific UCHL1 knockout (UCHL1‐CKO) and cardiac‐specific recombinant adeno‐associated virus serotype 9 (rAAV9)‐UCHL1‐overexpressing mice. We also investigated how UCHL1 regulates CaMKII‐δ stabilization and activates its downstream RyR2/Ca^2+^ signaling axis. The results of this study provide new insight into AF pathogenesis and identify a promising therapeutic target.

## 2 Results

### 2.1 Upregulation of UCHL1 in Mice and Patients With AF

To determine whether DUB expression is altered in Ang II‐infused mice with AF, we first profiled the mRNA and protein levels of DUB genes in saline‐ or Ang II‐infused atrial tissues of WT mice. Microarray analysis showed that three DUB genes were significantly upregulated (*p* < 0.05), whereas four were markedly downregulated (*p* < 0.05) at the mRNA level in atrial tissue of Ang II‐infused mice compared with those in saline‐treated controls (Figure 1A). Proteomic analysis showed that, compared with saline‐treated controls, four DUB proteins were upregulated (*p* < 0.05) and two were downregulated (*p* < 0.05) at the protein level in atrial tissue from Ang II‐infused mice (Figure 1B). Venn‐diagram analysis identified UCHL1 as the only DUB upregulated at the mRNA and protein levels (1.466‐fold at the mRNA level, *p* = 0.0313; 1.7‐fold at the protein level, *p* = 0.003) (Figure 1C). Furthermore, qPCR confirmed increased *UCHL1* mRNA in Ang II‐infused atrial tissues (Figure 1D). Immunoblotting and immunostaining further showed time‐dependent UCHL1 upregulation in Ang II‐treated mouse atrial tissues and cultured atrial cardiomyocytes (ACMs) (Figure 1E,F; Figure S1A), but not in atrial fibroblasts (Figure S1B). Co‐immunostaining for UCHL1 and the cardiomyocyte marker α‐actinin showed specific colocalization in ACMs, which increased markedly after Ang II stimulation (Figure 1G).

**FIGURE 1:**
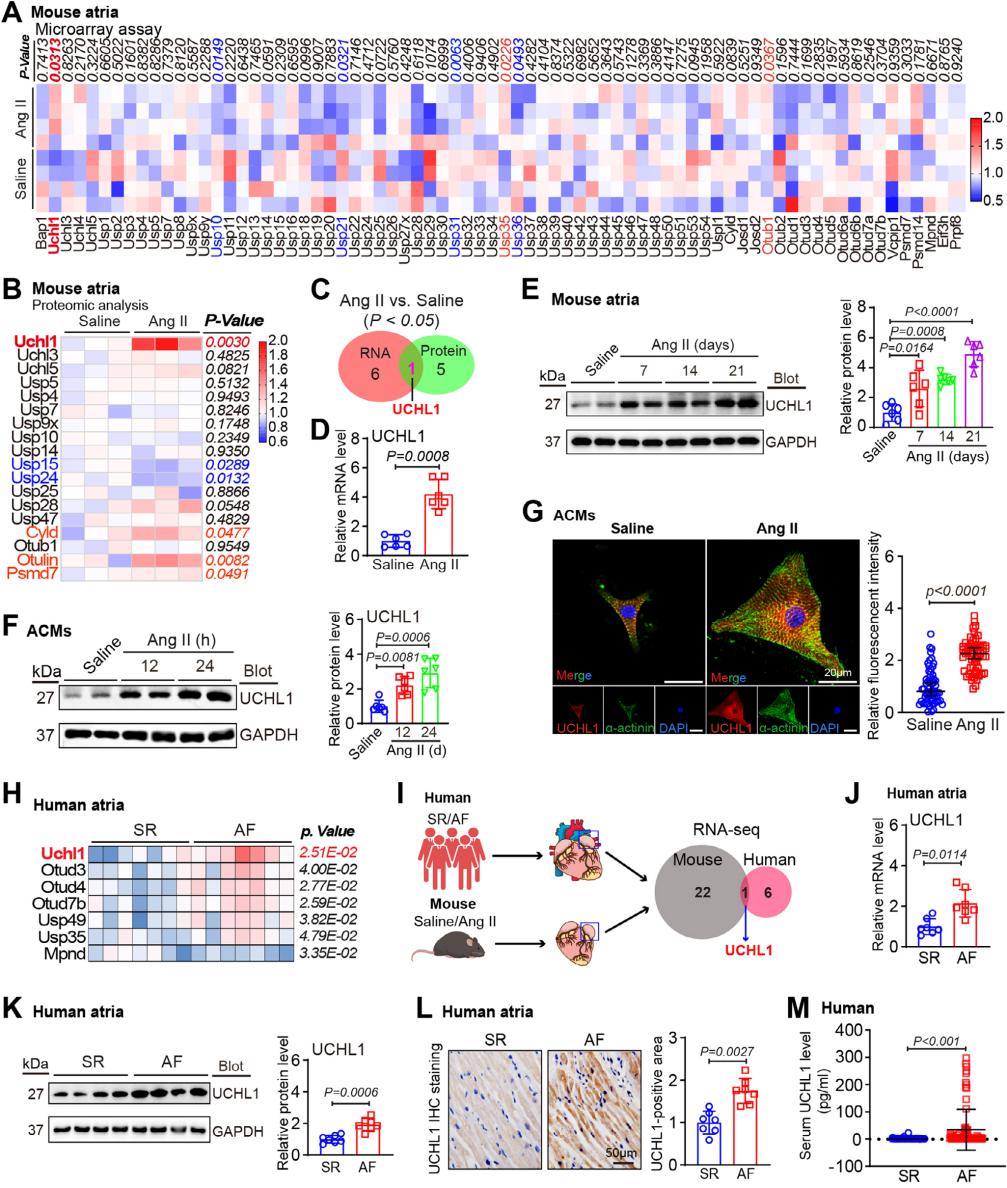
Upregulation of UCHL1 expression in atrial myocytes from mice and patients with atrial fibrillation (AF). (A) Microarray expression‐profile analysis of deubiquitinating enzyme (DUB) genes in atrial tissues of Ang II‐ and saline‐infused mice (n = 4 mice per group). *p* values were determined by unpaired two‐tailed Student's t‐test. (B) Heatmap showing the protein levels of all DUBs in the two atrial‐tissue groups identified by proteomic analysis (n = 3 mice per group). (C) Venn diagram showing the overlap of the differentially expressed DUB genes and proteins in the two atrial‐tissue groups. *p* <0.05 as the cutoff for both RNA and protein. (D) qPCR analysis of the mRNA expression of *UCHL1* in the atria of the two groups (n = 6 mice per group). (E) Representative immunoblot of UCHL1 in atria of the two groups (n = 6 mice per group). (F) Representative immunoblot of UCHL1 in primary atrial cardiomyocytes (ACMs) stimulated with saline or Ang II (100 nm) for 12 or 24 h (n = 6 independent experiments). (G) Representative images and quantification of immunofluorescence staining of UCHL1 (red) and α‐actinin (green) in ACMs 24 h after saline or Ang II treatment (n = 60 ACMs per group). (H) Heatmap of transcriptomic data in atria from patients with atrial fibrillation (AF) or sinus rhythm (SR). Differentially expressed DUB genes, including *UCHL1, OTUD3, OTUD4, OTUD7B, USP49, USP35*, and *MPND*, are shown (n = 7 patients per group). (I) Overlap of the differentially expressed DUB genes in atria from patients with AF and Ang II‐infused mice. *p <0.05* as the cutoff value. (J) qPCR analysis of UCHL1 in atrial tissues from patients with SR or AF (n = 7 patients per group). (K) Immunoblot analysis and quantitative analysis of UCHL1 protein in atria from patients with SR and AF. GAPDH was used as the loading control (n = 7 patients per group). (L) Immunohistochemical (IHC) staining of UCHL1 in atria from patients with SR or AF (n = 7 patients per group). (M) ELISA analysis of serum UCHL1 concentrations (pg/mL) in patients with SR (n = 50) and AF (n = 82). Data presented in E and F were analyzed using one‐way ANOVA followed by the Bonferroni post hoc test. Data presented in D, J, K, and L were analyzed using an unpaired Student's t‐test. Data in M were analyzed using Mann–Whitney U tests.

Next, we assessed UCHL1 expression in atrial tissues from patients with AF (n = 7) and sinus rhythm (SR) controls (n = 7). The clinical characteristics of the atrial‐biopsy cohorts are summarized in Table S1. RNA‐seq revealed that *UCHL1* was the most highly upregulated gene among the seven differentially expressed DUB genes in AF atrial tissue (fold change = 2.8125, *p* = 0.0251) (Figure 1H). Importantly, RNA‐seq analysis verified that *UCHL1* expression was consistently increased in atrial tissues from both Ang II‐infused mice and patients with AF compared with that in controls (Figure 1I). Validation using qPCR, immunoblotting, and immunohistochemical (IHC) staining confirmed increased UCHL1 expression at the mRNA and protein levels in atrial tissue of patients with AF (Figure 1J–L). Additionally, serum UCHL1 levels were significantly higher in patients with AF than in SR controls (Figure 1M). The clinical data of patients with AF (n = 82) and SR controls (n = 50) included in the serum‐sample analysis are summarized in Table S2. Finally, we evaluated the association between serum UCHL1 levels and AF incidence using univariable and multivariable logistic regression models (Table S3). After adjusting for sex, age, systolic blood pressure (SBP), serum creatinine, brain natriuretic peptide, left atrial (LA) diameter, and high‐density lipoprotein cholesterol, serum UCHL1 levels (OR = 1.293) remained significantly associated with AF incidence (Table S3). Collectively, these results suggest that increased UCHL1 in ACMs may be associated with atrial remodeling and AF pathogenesis.

### 2.2 Atrial Cardiomyocyte‐Specific Deletion of UCHL1 Attenuates Ang II‐Induced AF Susceptibility

To evaluate the functional role of UCHL1 in AF development, we first generated global heterozygous UCHL1‐knockdown (KD) mice and used age‐matched wild‐type (WT) littermates as controls (Figure S2A), because homozygous UCHL1‐knockout (UCHL1‐KO) mice die prematurely at 12 weeks of age. Immunoblotting confirmed a significant reduction in endogenous UCHL1 protein levels in the atrial tissues of UCHL1‐KD mice compared with those in WT controls (Figure S2B). After 3 weeks of angiotensin II (Ang II) infusion, intracardiac electrograms (EGMs) showed that UCHL1‐KD mice exhibited significantly lower AF inducibility (45% versus 80%) and duration (1636 versus 3186 ms) as well as less LA enlargement and a smaller atrial fibrotic area (Figure S2C–F). Additionally, atrial macrophage infiltration (CD68 staining), reactive oxygen species (ROS) levels (DHE staining), and the expression of fibrotic, proinflammatory, and pro‐oxidant markers (*COL1A1, COL3A1, IL‐1β*, and *CYBB/NOX2*) were all significantly lower in the atrial tissues of UCHL1‐KD mice than in those of WT mice (Figure S2G–I).

To further investigate whether atrial cardiomyocyte‐specific UCHL1 deletion modulates AF susceptibility, we next generated ACM‐specific UCHL1‐CKO mice by crossbreeding mice bearing *loxP*‐flanked UCHL1 (*UCHL1^fl/fl^*) alleles with ACM‐specific Cre (NPPA‐Cre) mice. *UCHL1^fl/fl^* littermates without Cre were used as controls (CTR) (Figure S3A–C). Subsequently, both UCHL1‐CKO and CTR mice were infused with Ang II (2000 ng/kg/min) for 3 weeks and were then subjected to electrical stimulation (Figure 2A). Immunoblotting confirmed an approximately 80% reduction of UCHL1 in atrial tissues (Figure 2B) but not in other tissues, such as ventricular myocardium or skeletal muscle from saline‐ or Ang II‐infused mice (Figure S3D). Similar to the observations from Ang II‐infused systemic UCHL1‐KD mice, systolic blood pressure (SBP) did not differ significantly between the UCHL1‐CKO and CTR groups during saline or Ang II infusion (Figure S3E). However, intracardiac electrograms (EGMs) revealed significantly lower AF inducibility (40% versus 80%) and a shorter AF duration (1565 versus 3030 ms) in UCHL1‐CKO mice than CTR mice after 3 weeks of Ang II infusion (Figure 2C,D). Moreover, the Ang II‐induced increase in LA diameter (LAD) in the CTR mice was also markedly attenuated in UCHL1‐CKO mice at week 3 of infusion (Figure 2E). Because atrial fibrosis, inflammation, and oxidative stress contribute to atrial structural remodeling and AF [27], we next examined these pathological processes. After 3 weeks of Ang II infusion, we observed marked increases in atrial fibrotic area (Masson staining), percentages of CD68‐positive macrophages (immunostaining), levels of superoxide (DHE staining), and the mRNA levels of *COL1A1, COL3A1, IL‐1β*, and *CYBB/NOX2* in CTR mice; however, these changes were markedly reduced in UCHL1‐CKO mice (Figure 2F–I). Further analysis revealed that multiple signaling pathways involved in atrial structural and electrical remodeling, including Ang II‐induced increases in TGF‐β (a key driver of fibrosis), NOX2 (a major source of ROS), NOD‐like pyrin domain‐containing protein 3 (NLRP3) (a key regulator of *IL‐1β* release), and potassium channel Kv1.5 (a critical regulator of atrial electrical conduction and repolarization), were all markedly reduced in the atria of UCHL1‐CKO mice compared with those in the atria of CTR mice (Figure 2J), consistent with the pathological and molecular alterations associated with AF. Overall, these data indicate that atrial cardiomyocyte‐specific UCHL1 knockout attenuates atrial remodeling and susceptibility to AF.

**FIGURE 2:**
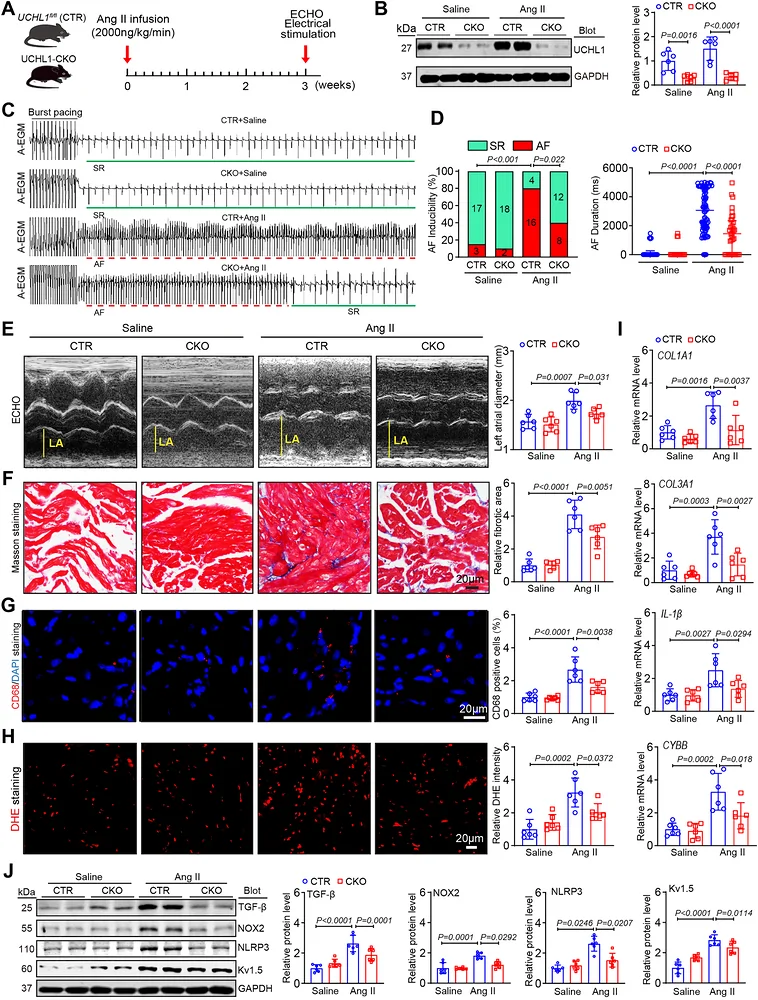
Atrial cardiomyocyte‐specific deficiency of UCHL1 alleviates Ang II‐induced AF susceptibility. (A) Schematic diagram of the induction procedure for the AF mouse model. Atrial myocyte‐specific UCHL1‐knockout mice (*UCHL1^fl/fl^*/NPPA‐Cre+, UCHL1‐CKO; 8‐week‐old male) and age‐matched littermates (*UCHL1^fl/fl^*, CTR, male) were infused with saline or Ang II (2000 ng/kg/min) for 3 weeks and then subjected to electrical stimulation. (B) Representative immunoblot (left) and quantification (right) of UCHL1 protein levels in atria (n = 6 mice per group). (C) Representative intracardiac electrogram recordings in CTR and UCHL1‐CKO mice after infusion and burst pacing. The red dotted lines indicate AF, and the green solid lines indicate SR. (D) Percentage of successful AF induction and duration of AF (n = 20 mice per group). (E) Representative images of M‐mode echocardiography (Echo) of left atrial diameter (LAD, left) and quantification of LAD (right, n = 6 mice per group). (F–H) Representative images (left) and quantification (right) of histological staining, including Masson's staining (blue), immunofluorescence staining for CD68‐positive cells (red for CD68, blue for DAPI), and DHE staining (red for reactive oxygen species [ROS]) (n = 6 mice per group). Scale bar: 20 µm. (I) qPCR analysis of the mRNA expression of *COL1A1*, *COL3A1*, *IL‐1β*, and *CYBB* (NOX2) in atria (n = 6 mice per group). (J) Representative immunoblot (left) and quantification (right) of TGF‐β, NOX2, NLRP3, and Kv1.5 in atria. The results are presented as the mean ± SD or percentage. Data in B, E–J were analyzed using two‐way ANOVA followed by the Bonferroni post hoc test. Data in D were analyzed using Fisher's exact test for the proportion of successful AF induction, and Mann–Whitney U tests followed by Dunn's multiple‐comparison test for duration of AF.

### 2.3 Cardiac‐Specific Overexpression of UCHL1 Increases AF Inducibility

To further verify the role of UCHL1 in AF development, we generated an rAAV9 vector overexpressing Flag‐tagged UCHL1 (rAAV9‐UCHL1) or an empty vector (rAAV9‐EV) and then injected them into WT mice for 2 weeks, followed by saline or Ang II infusion for another 3 weeks (Figure 3A). Immunoblotting assays confirmed that rAAV9‐UCHL1 injection markedly increased UCHL1 protein levels compared with rAAV9‐EV injection (Figure 3B). No significant differences in SBP were observed between rAAV9‐UCHL1‐ and rAAV9‐EV‐injected mice under saline or Ang II infusion (Figure S4). However, Ang II‐induced AF inducibility (100% versus 75%) and duration (3541 versus 2812 ms) were significantly greater in rAAV9‐UCHL1‐injected mice than in rAAV9‐EV‐injected controls (Figure 3C,D), indicating that UCHL1 functions as a driver of Ang II‐induced AF. Moreover, UCHL1 overexpression exacerbated Ang II‐induced increases in LAD, atrial fibrotic area, CD68‐positive macrophage infiltration, and ROS levels compared with those in the rAAV9‐EV group at week 3 of treatment (Figure 3E–H). Consistently, Ang II‐induced upregulation of *COL1A1, COL3A1, IL‐1β*, and *NOX2* mRNA levels in the atria of rAAV9‐EV‐injected controls was further enhanced in rAAV9‐UCHL1‐injected mice (Figure 3I). In addition, immunoblotting showed that the protein levels of TGF‐β, NOX2, NLRP3, and Kv1.5 in the atria of rAAV9‐UCHL1‐injected mice were significantly higher than those in rAAV9‐EV‐injected mice following Ang II infusion (Figure 3J). Together, these results demonstrate that UCHL1 overexpression promotes atrial remodeling and plays a proarrhythmic role in the progression of AF.

**FIGURE 3:**
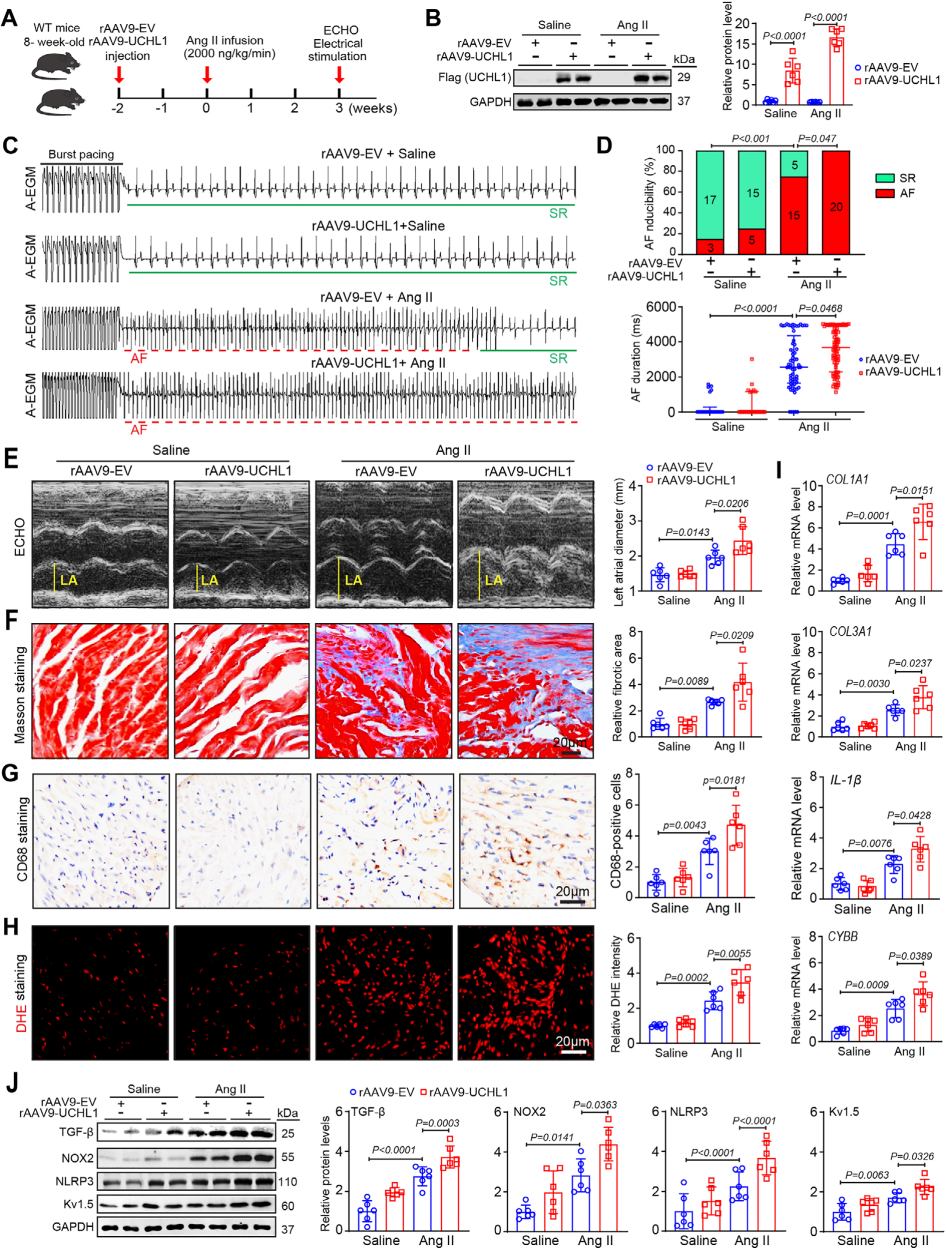
Cardiac‐specific UCHL1 overexpression aggravates Ang II‐induced AF susceptibility. (A) Schematic diagram of rAAV9 injection and AF mouse model establishment. Wild‐type (WT) (C57BL/6J, 8‐week‐old male) mice were injected with rAAV9‐EV (empty vector) or rAAV9‐UCHL1 for 2 weeks and then infused with saline and Ang II for another 3 weeks. (B) Representative immunoblots (left) and quantification (right) of UCHL1 protein levels using anti‐Flag antibody in atria (n = 6 mice per group). (C) Representative intracardiac atrial electrogram recordings in rAAV9‐EV or rAAV9‐UCHL1‐injected mice after burst pacing. Red dotted line represents AF, and green solid line represents SR. (D) Percentage of successful AF induction (left) and duration of AF (right; n = 20 mice per group). (E) Representative images of M‐mode echocardiography of LAD (left) and quantification (right) of LAD (n = 6 mice per group). (F) Representative images of Masson's staining (left) and quantification (right) of atrial fibrosis (n = 6 mice per group). Scale bar: 20 µm. (G) Representative images of IHC staining for CD68‐positive cells (n = 6 mice per group). Scale bar: 20 µm. (H) Representative images of DHE staining (left) and quantification (right) of superoxide levels in atria (n = 6 mice per group). (I) qPCR analysis of the mRNA expression of *COL1A1, COL3A1, IL‐1β*, and *CYBB* (NOX2) in atria (n = 6 mice per group). (J) Representative immunoblots (left) and quantification (right) of TGF‐β, NOX2, NLRP3, and Kv1.5 in atria (n = 6 mice per group). The results are presented as the mean ± SD or percentage. Data in B and E–J were analyzed using two‐way ANOVA followed by the Bonferroni post hoc test. Data in D were analyzed using Fisher's exact test for the proportion of successful AF induction, and Mann–Whitney U tests followed by Dunn's multiple‐comparison test for duration of AF.

### 2.4 UCHL1 Physically Binds to CaMKII‐δ

Given the critical role of UCHL1 in AF (Figures 2 and 3), we sought to elucidate the molecular mechanism by which UCHL1 regulates AF. First, we performed co‐immunoprecipitation (Co‐IP) coupled with liquid chromatography‐tandem mass spectrometry (LC‐MS/MS) to identify potential UCHL1‐interacting proteins in ACMs using an anti‐UCHL1 antibody or an immunoglobulin G (IgG) control (Figure 4A). Among 96 proteins copurified with UCHL1, we found that CaMKII‐δ (CaMK2d), a member of the calcium/calmodulin‐activated protein kinase family, ranked third among the UCHL1‐binding proteins (Figure 4B). CaMKII‐δ is a critical proarrhythmic signal in AF [4]. To validate the association between UCHL1 and CaMKII‐δ, we predicted the interaction using the ZDOCK server, focusing on UCHL1 (2–221 aa) and CaMKII‐δ (8–301 aa) (Figure 4C). Co‐IP assays conducted both in vivo and in vitro using anti‐UCHL1 or anti‐CaMKII‐δ antibodies confirmed the interaction between endogenous UCHL1 and CaMKII‐δ in mouse atrial tissues and cultured ACMs (Figure 4D,E), with Ang II treatment further enhancing this association (Figure 4F).

**FIGURE 4:**
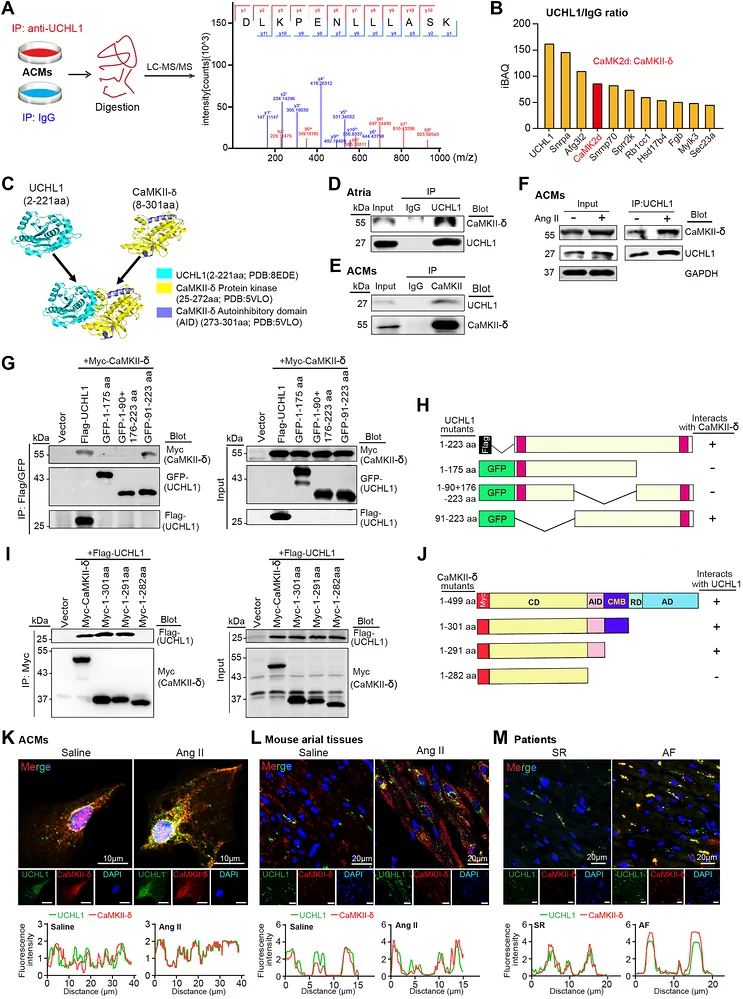
UCHL1 directly interacts with CaMKII‐δ. (A) Schematic illustration of interactome analysis for UCHL1 and identification of a peptide sequence (DLKPENLLLASK) corresponding to CaMK2d (CaMKII‐δ). (B) Relative abundance of 11 proteins immunoprecipitated by anti‐UCHL1 compared with IgG control, as indicated by UCHL1‐immunoprecipitation (IP) score/IgG‐IP score ratios. (C) Prediction of the interaction between UCHL1 proteins (2–221 aa) and CaMKII‐δ proteins (8–301 aa) using the ZDOCK server. The predicted structure was visualized using PyMOL software. (D) IP analysis for the interaction between UCHL1 and CaMKII‐δ in atria with anti‐UCHL1 antibody or IgG control (n = 3 independent experiments). (E) IP analysis for the interaction between CaMKII‐δ and UCHL1 in ACMs using anti‐CaMKII‐δ antibody or IgG (n = 3 independent experiments). (F) IP assay for the interaction between UCHL1 and CaMKII‐δ in Ang II‐stimulated ACMs (n = 3 independent experiments). (G) IP assay of the interaction between UCHL1 and CaMKII‐δ. HEK293T cells were co‐transfected with Flag‐tagged full‐length (FL) or GFP‐tagged truncated UCHL1 and Myc‐tagged FL CaMKII‐δ. Lysates were immunoprecipitated with anti‐Flag or anti‐GFP antibody (n = 3 independent experiments). (H) Mapping of the interactions between UCHL1 domains and CaMKII‐δ used for the IP assay in G. (I) IP assay for interaction between CaMKII‐δ domains and UCHL1. HEK293T cells were co‐transfected with Myc‐tagged full‐length or truncated CaMKII‐δ and Flag‐tagged FL UCHL1. Lysates were immunoprecipitated with anti‐Myc antibody (n = 3 independent experiments). (J) Mapping of the interactions between CaMKII‐δ domains and UCHL1 used for the IP assay in I. CD, catalytic domain; AID, autoinhibitory domain; CMB, calmodulin‐binding domain; RD, regulatory domain; AD, association domain; (K) Confocal microscopy images of immunofluorescence staining showing that CaMKII‐δ (red) colocalizes with UCHL1 (green) in ACMs. Scale bar: 10 µm; n = 3 independent experiments. (L) Immunofluorescence staining for CaMKII‐δ (red), UCHL1 (green), and DAPI (blue) in atria from saline‐ or Ang II‐infused mice (n = 6 mice per group). Scale bar: 20 µm. (M) Immunofluorescence staining for CaMKII‐δ (red), UCHL1 (green), and DAPI (blue) in atria from patients with SR or AF (n = 6 patients per group). Scale bar: 20 µm.

To further investigate the specific regions mediating the UCHL1–CaMKII‐δ interaction, Co‐IP assays were performed in HEK293T cells transfected with Flag‐ or GFP‐tagged full‐length UCHL1 or Myc‐tagged full‐length CaMKII‐δ and their respective truncation constructs. These experiments demonstrated that amino acids 91–223 of UCHL1 mediate its interaction with CaMKII‐δ (Figure 4G,H), whereas the autoinhibitory domain (AID; 283–291 aa) of CaMKII‐δ was required for the association of CaMKII‐δ with UCHL1 (Figure 4I,J), suggesting that the central region of UCHL1 and the AID of CaMKII‐δ are required for their interaction.

Consistent with these findings, immunostaining and confocal microscopy confirmed the colocalization of UCHL1 and CaMKII‐δ primarily in the cytoplasm of ACMs (Figure 4K), mouse atria (Figure 4L), and atrial tissues of patients with AF (Figure 4M). Notably, the expression levels of both UCHL1 and CaMKII‐δ were markedly increased in Ang II‐treated ACMs (Figure 4K) in mouse atrial tissues and atrial tissues from patients with AF (Figure 4L,M). Collectively, these results demonstrate that UCHL1 physically interacts with CaMKII‐δ in ACMs.

### 2.5 UCHL1 Deubiquitinates CaMKII‐δ

Previous studies have reported ubiquitination of CaMKII isoforms (e.g., CaMKIIα and CaMKIIβ) [11, 12], but the deubiquitination of CaMKII‐δ and its functional relevance in AF remain uncharacterized. To determine whether CaMKII‐δ is a deubiquitination substrate of UCHL1 in ACMs, we purified ubiquitinated proteins from ACMs infected with an adenoviral vector expressing UCHL1 (Ad‐UCHL1) or an empty vector (Ad‐EV) and analyzed them using Co‐IP combined with LC‐MS/MS (Figure 5A). The analysis revealed the sequence of ubiquitination sites and their flanking amino acids in UCHL1 target proteins (Figure S5A,B). Cluster analysis of differentially ubiquitinated sites defined four distinct ubiquitinome subgroups (Q1–Q4) (Figure S5C). In addition, protein domain enrichment analysis revealed that the differentially ubiquitinated proteins (DUPs) contained many functional domains, including protein kinase domains in multiple kinases (Figure S5C). Overall, ubiquitination was reduced in 1881 modified proteins in UCHL1‐overexpressing ACMs (Figure 5B). Among 96 UCHL1‐binding proteins identified in the interactome (Figure 4A), 30 overlapped with the DUPs. Of note, we identified a reduction in ubiquitination of CaMKII‐δ at lysine 251 (K251) in UCHL1‐overexpressing ACMs by mass spectrometry‐based proteomics (Figure 5C). A ubiquitination assay further confirmed that ubiquitination of CaMKII‐δ (WT), but not CaMKII‐δ (K251R), was markedly reduced by UCHL1 overexpression, suggesting that CaMKII‐δ is a potential target of UCHL1 and is ubiquitinated primarily at K251 (Figure 5D).

**FIGURE 5:**
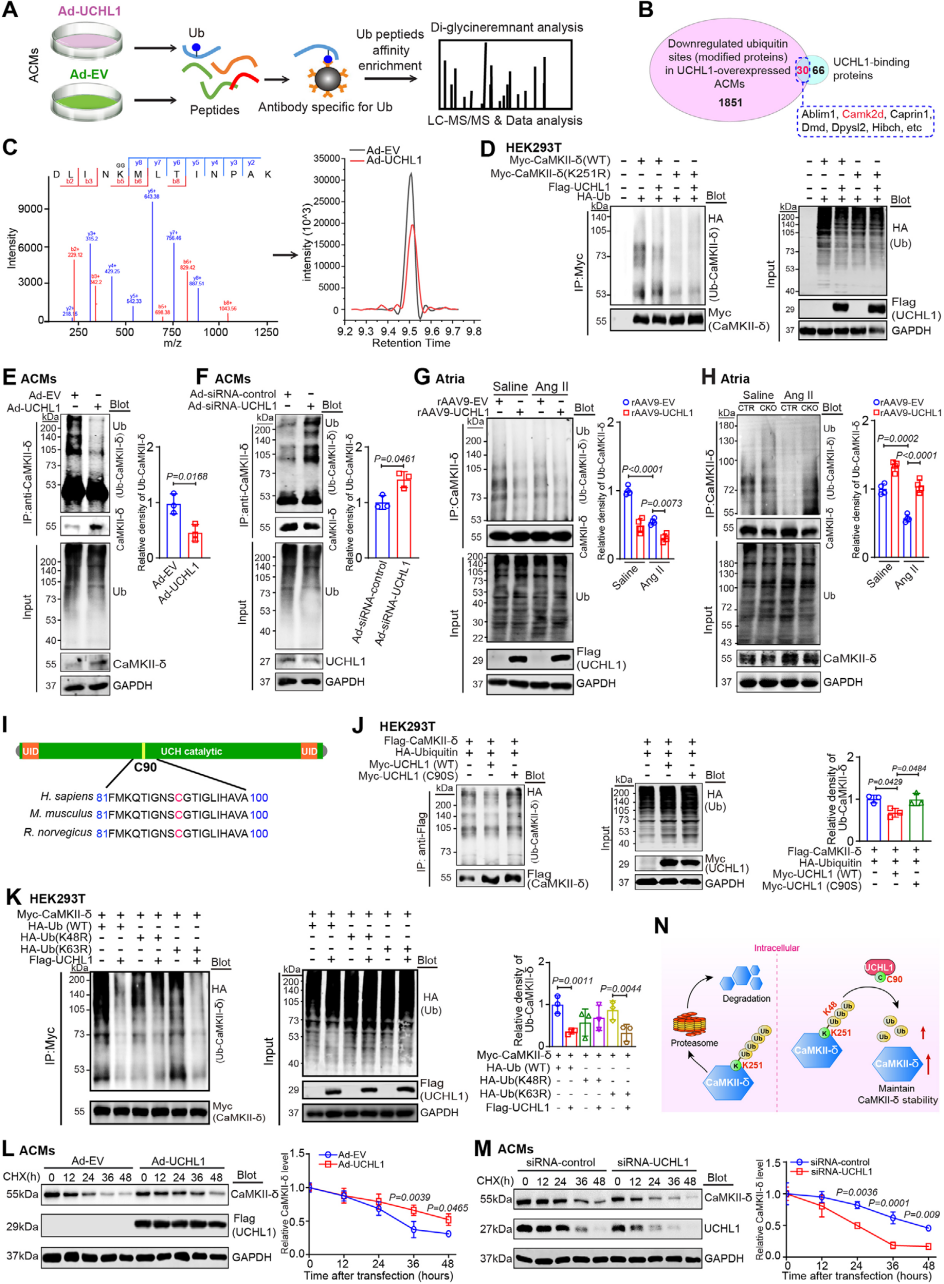
UCHL1 promotes CaMKII‐δ deubiquitination and stabilization. (A) Schematic illustration of the target protein profile of UCHL1 in cultured ACMs using ubiquitin (Ub) antibody‐conjugated beads to enrich modified peptides, followed by high‐resolution liquid chromatography‐tandem mass spectrometry (LC‐MS/MS) analysis. (B) Venn diagram depicting the proteins with reduced ubiquitination in UCHL1‐overexpressing ACMs and UCHL1‐interacting proteins in ACMs. (C) Mass spectrometry identified ubiquitination of CaMKII‐δ at lysine 251 (K251) in Ad‐EV‐ or Ad‐UCHL1‐infected ACMs, and a reduction of the modified peptide was observed in UCHL1‐overexpressing ACMs using HRM XICs. (D) Immunoprecipitation of lysates from HEK293T cells transfected with Myc‐CaMKII‐δ (WT), Myc‐CaMKII‐δ (K251R), Flag‐UCHL1, and HA‐Ub; immunoprecipitation was performed with an anti‐Myc antibody, followed by immunoblot analysis with anti‐HA, anti‐Myc, and anti‐Flag antibodies. (E, F) Ubiquitination assay (left) and quantification (right) of CaMKII‐δ in cultured ACMs infected with an adenovirus containing an empty vector (Ad‐EV), Ad‐UCHL1, Ad‐siRNA‐control, or Ad‐siRNA‐UCHL1, and treated with MG132 (10 µm) 6 h before harvest (n = 3 independent experiments). (G, H) Ubiquitination assay and quantification of CaMKII‐δ in atria from UCHL1^fl/fl^ (CTR), UCHL1‐CKO, rAAV9‐EV‐ and rAAV9‐UCHL1‐injected mice after saline or Ang II infusion (n = 4 independent experiments). (I) Schematic illustration of the UCHL1 active site (C90). (J) Ubiquitination assay of CaMKII‐δ in HEK293T cells transfected with Myc‐WT UCHL1 or Myc‐UCHL1 (C90S) together with Flag‐CaMKII‐δ and HA‐ubiquitin (Ub) and treated with MG132 (10 µm) for 6 h before harvest (n = 3 independent experiments). (K) HEK293T cells transfected with HA‐Ub (WT), HA‐Ub (K48R), or HA‐Ub (K63R) together with Myc‐CaMKII‐δ and Flag‐UCHL1 and treated with MG132 (10 µm) for 6 h before harvest. Lysates were immunoprecipitated with anti‐Myc antibody followed by immunoblot analysis with anti‐HA, anti‐Myc, and anti‐Flag antibodies, and quantification (right) of Ub‐CaMKII‐δ (detected by anti‐HA) (n = 3 independent experiments). (L, M) CHX chase assay for the half‐life of CaMKII‐δ. ACMs were infected with Ad‐EV and Ad‐UCHL1 or Ad‐siRNA‐control or Ad‐siRNA‐UCHL1 for 24 h and then treated with CHX (10 µm) for the indicated time points (left), with quantification (right) of CaMKII‐δ protein (n = 3 independent experiments). (N) Schematic of the mechanism by which UCHL1 deubiquitinates CaMKII‐δ. The results are presented as the mean ± SD. Data in E and F were analyzed using a two‐tailed unpaired t‐test. Data presented in G, H, K, L, and M were analyzed using two‐way ANOVA followed by the Bonferroni post hoc test. Data in J were analyzed using one‐way ANOVA followed by the Bonferroni post hoc test.

To further validate whether UCHL1 mediated the deubiquitination of CaMKII‐δ in vitro, ACMs were infected with Ad‐UCHL1, Ad‐EV, Ad‐siRNA‐UCHL1, or Ad‐siRNA‐control and treated with the proteasome inhibitor MG132 to inhibit protein degradation. Co‐IP and immunoblotting showed that ubiquitination levels of CaMKII‐δ were significantly lower in Ad‐UCHL1‐infected cells than in Ad‐EV controls, but substantially higher in Ad‐siRNA‐UCHL1 cells than in Ad‐siRNA‐control (Figure 5E,F), indicating that UCHL1 deubiquitinates CaMKII‐δ in cultured ACMs. Consistently, in mouse atrial tissues, CaMKII‐δ ubiquitination was reduced in rAAV9‐UCHL1–overexpressing mice and enhanced in UCHL1‐CKO mice during both saline and Ang II infusion (Figure 5G,H).

Because DUBs can prevent protein degradation by cleaving ubiquitin from target substrates, we further investigated whether UCHL1 uses its deubiquitinase activity to hydrolyze ubiquitin linkages from CaMKII‐δ. The cysteine at position 90 (C90) is a catalytic residue required for UCHL1 deubiquitinase activity [28]. The C90 residue and its surrounding residues are conserved in many species, including *Mus musculus, Homo sapiens*, and *Rattus norvegicus* (Figure 5I). We then constructed a UCHL1‐C90S mutant plasmid (cysteine‐to‐serine substitution at C90) to examine the active site of UCHL1. HEK293T cells were co‐transfected with Flag‐tagged CaMKII‐δ and HA‐tagged ubiquitin together with either Myc‐tagged UCHL1‐WT or UCHL1‐C90S. As expected, overexpression of UCHL1‐WT markedly decreased the ubiquitination levels of CaMKII‐δ, whereas UCHL1‐C90S did not reduce CaMKII‐δ ubiquitination (Figure 5J), indicating that UCHL1 deubiquitinates CaMKII‐δ in a manner dependent on its catalytic activity.

Subsequently, we sought to determine the specificity of UCHL1 for different ubiquitin chain linkages, ubiquitination assays were performed in HEK293T cells co‐transfected with plasmids expressing Myc‐CaMKII‐δ, Flag‐UCHL1 together with HA‐Ub‐WT, HA‐Ub‐K48R (lysine 48 mutated to arginine) or HA‐Ub‐K63R (lysine 63 mutated to arginine), respectively. Co‐IP with anti‐Myc antibody and immunoblotting assay with anti‐HA revealed that Flag‐UCHL1 transfection markedly reduced CaMKII‐δ ubiquitination in cells transfected with HA‐Ub‐WT and HA‐Ub‐K63, but not HA‐Ub‐K48 (Figure 5K). Moreover, we performed immunoprecipitation with anti‐CaMKII‐δ antibody in lysates from primary ACMs infected with Ad‐EV or Ad‐UCHL1, and found that Ad‐UCHL1 overexpression markedly attenuated K48‐linked polyubiquitination of CaMKII‐δ, which was confirmed using K48‐ and K63‐linkage‐specific antibodies (Figure S6A,B). Subsequently, we assessed the impact of UCHL1 on CaMKII‐δ degradation in cultured ACMs infected with Ad‐UCHL1, Ad‐EV, Ad‐siRNA‐UCHL1, or Ad‐siRNA‐control when protein synthesis was inhibited by cycloheximide (CHX). The CHX chase assay showed that UCHL1 overexpression prolonged the half‐life of CaMKII‐δ in a time‐dependent manner (36 and 48 h) (Figure 5L), whereas UCHL1 knockdown shortened the half‐life from 36 to 24 h post CHX exposure (Figure 5M). Moreover, the K251R mutant had a longer basal half‑life and was not further stabilized by UCHL1 (Figure S7A), confirming that UCHL1 prolongs CaMKII‑δ stability via K251. Notably, neither overexpression nor knockdown of UCHL1 affected CaMKII‐δ transcription in ACMs (Figure S8A,B). Overall, UCHL1 selectively cleaves K48‐linked polyubiquitin chains from CaMKII‐δ at position K251 through its catalytic activity at residue C90, thereby enhancing its stability (Figure 5N).

To further explore whether the enzymatic activity of UCHL1 at residue C90 is required for its proarrhythmic effect in vivo, we generated rAAV9‐EV, rAAV9‐UCHL1‐WT, and rAAV9‐UCHL1‐C90S (catalytically inactive mutant of UCHL1 with a cysteine at position 90 replaced by serine), which were delivered into CTR or UCHL1‐CKO mice for 2 weeks, followed by 3 weeks of Ang II infusion (Figure S9A). Immunoblotting revealed that injection of rAAV9‐UCHL1‐WT and rAAV9‐UCHL1‐C90S resulted in higher UCHL1 protein levels than rAAV9‐EV injection (Figure S9B). Intracardiac electrograms showed lower AF inducibility (40% versus 80%) and a shorter AF duration (1269 versus 2938 ms) in UCHL1‐CKO mice injected with rAAV9‐EV than in CTR mice injected with rAAV9‐EV. A similar effect was observed in UCHL1‐CKO mice injected with rAAV9‐UCHL1‐C90S, whereas this effect was markedly reversed in UCHL1‐CKO mice injected with rAAV9‐UCHL1‐WT (Figure S9C,D). Accordingly, after rAAV9‐EV injection, UCHL1‐CKO mice showed decreased LAD and atrial fibrosis compared with CTR mice. In UCHL1‐CKO mice, overexpression of UCHL1‐C90S also reduced LAD dilatation and fibrosis. However, overexpression of UCHL1‐WT produced the opposite effects (Figure S9E–H). Thus, these findings indicate that the deubiquitinase activity of UCHL1 is essential for its contribution to AF development in vivo.

Additionally, we examined whether the K251 ubiquitination site in CaMKII‐δ is critical for UCHL1‐mediated CaMKII‐δ stabilization and AF susceptibility in vivo. We generated an rAAV9 vector expressing WT CaMKII‐δ, CaMKII‐δ‐K251R (a ubiquitination‐deficient mutant of CaMKII‐δ with lysine 251 replaced by arginine), or an empty vector (EV) under the atrial myocyte‐specific NPPA promoter and then delivered them into CTR or UCHL1‐CKO mice for 2 weeks, followed by 3 weeks of Ang II infusion (Figure S10A). The protein levels of UCHL1 were lower in UCHL1‐CKO mice than in CTR mice, whereas CaMKII‐δ protein levels were higher in UCHL1‐CKO mice injected with rAAV9‐CaMKII‐δ and rAAV9‐CaMKII‐δ‐K251R than in those injected with rAAV9‐EV (Figure S10B). Compared with CTR mice, UCHL1‐CKO mice displayed reduced inducibility (45% versus 80%) and duration (1185 versus 2398 ms) of AF, LAD dilatation, atrial fibrosis, and p‐RyR2 levels after rAAV9‐EV injection and Ang II infusion. Conversely, overexpression of rAAV9‐CaMKII‐δ in UCHL1‐CKO mice markedly increased AF inducibility, LAD enlargement, and fibrosis. However, these effects were further enhanced by overexpression of CaMKII‐δ‐K251R in the same mice (Figure S10C–J). Together, these results indicate that K251 in CaMKII‐δ is an essential residue for UCHL1‐mediated CaMKII‐δ stabilization and proarrhythmic function.

### 2.6 UCHL1 Stabilizes CaMKII‐δ and Facilitates RyR2‐Mediated Calcium Mishandling

CaMKII‐δ can induce aberrant phosphorylation of RyR2 at S2814, a pivotal mechanism that disrupts Ca^2+^ handling and promotes AF [29, 30, 31]. To establish the role of UCHL1 in regulating endogenous CaMKII‐δ stability and RyR2 activation in the atria, we examined changes in CaMKII‐δ levels and RyR2 phosphorylation in UCHL1‐CKO and rAAV9‐UCHL1‐overexpressing mice. Immunoblotting revealed that Ang II significantly increased the protein levels of CaMKII‐δ and phosphorylated (p)‐RyR2 at S2814 in the atria of CTR mice, whereas this increase was markedly attenuated in the atria of UCHL1‐CKO mice (Figure 6A). Conversely, Ang II‐induced upregulation of CaMKII‐δ and p‐RyR2 (S2814) protein levels was further enhanced in the atria of rAAV9‐UCHL1‐injected mice (Figure 6B). Notably, the UCHL1‐overexpression‐mediated increase in both CaMKII‐δ and p‐RyR2 protein levels in the atria was abolished by rAAV9‐siCaMKII‐δ injection in rAAV9‐UCHL1‐ or rAAV9‐EV‐injected mice after Ang II infusion (Figure 6C). Therefore, these observations indicate that UCHL1 increases CaMKII‐δ stability, leading to RyR2 activation in the atria following Ang II infusion.

**FIGURE 6:**
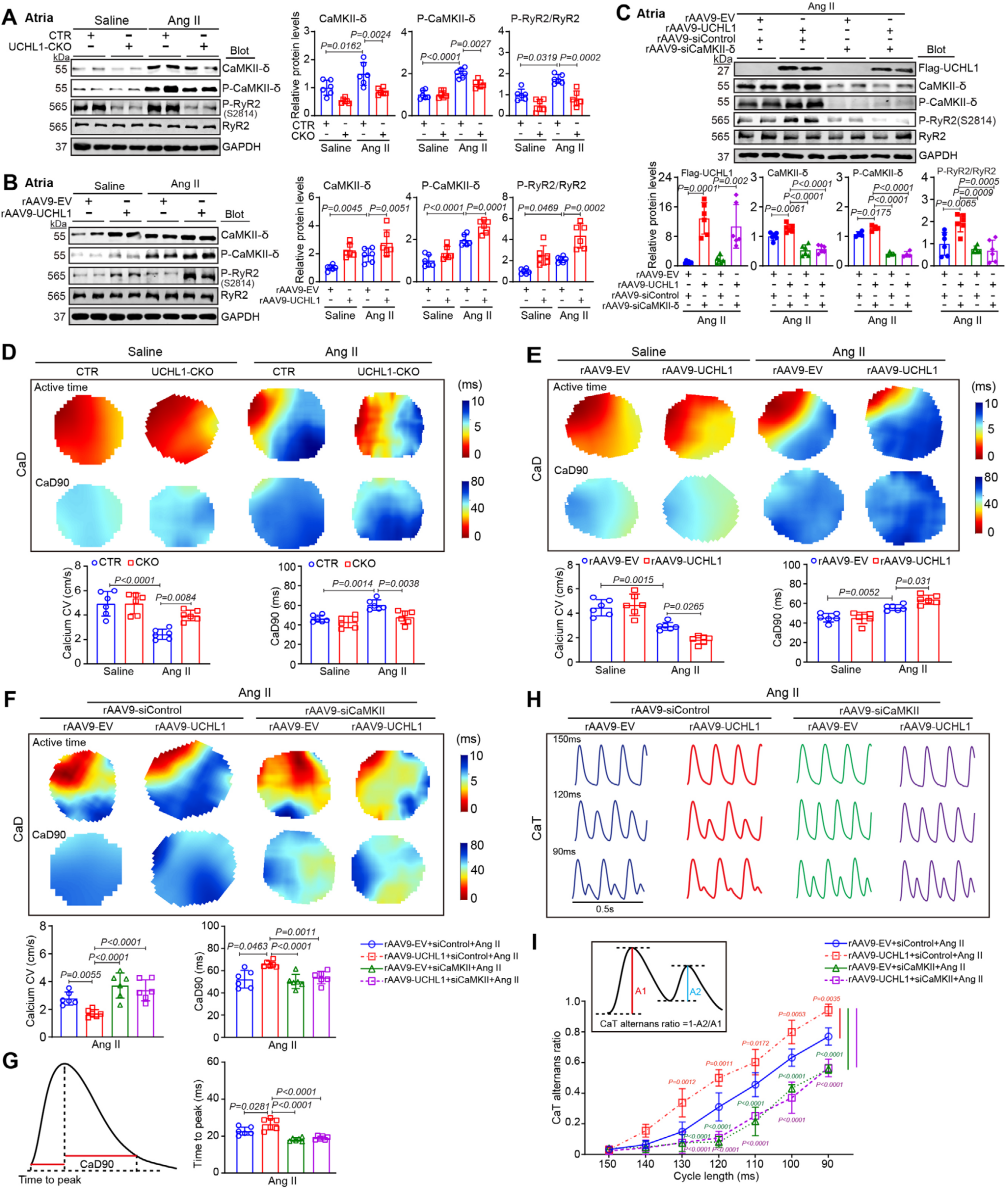
UCHL1 regulates atrial calcium handling through activation of CaMKII‐RyR2 signaling in mice. (A) Representative immunoblots (left) and quantitative analysis (right, n = 6 mice per group) of CaMKII‐δ, total and phosphorylated (p)‐RyR2 (S2814) protein levels in the atria of CTR and UCHL1‐CKO mice after saline or Ang II infusion. (B) Representative immunoblotting (left) and quantification (right, n = 6 mice per group) of CaMKII‐δ, total and p‐RyR2 (S2814) protein levels in the atria of rAAV9‐EV‐ and rAAV9‐UCHL1‐injected mice after saline or Ang II infusion. (C) Representative immunoblots (top) and quantification (bottom, n = 6 mice per group) of Flag (UCHL1), CaMKII‐δ, total and p‐RyR2 (S2814) protein levels in the atria of four groups after Ang II infusion. (D) Representative heatmaps of conduction velocity (CV) and calcium (Ca^2+^) transient duration at 90% repolarization (CaD90) at 8 Hz pacing cycle length (PCL) of 125 ms in the atria from CTR and UCHL1‐CKO mice after saline or Ang II infusion (top). Quantification of CV and CaD90 during the CaT phase (bottom, n = 6 mice per group). (E) Representative heatmaps of CV and CaD90 at 8 Hz PCL of 125 ms in the atria of rAAV9‐EV‐ and rAAV9‐UCHL1‐injected mice (top). Quantification of CV and CaD90 in the CaT phase (bottom, n = 6 mice per group). (F) Representative heatmaps of CV and CaD90 at 8 Hz PCL of 125 ms in the atria of four groups after Ang II infusion (top). Quantification of CV and CaD90 during the CaT phase in the four groups (bottom, n = 6 mice per group). (G) Schematic illustration of time‐to‐peak (TTP) and CaD90 (left). Quantification of TTP (right, n = 6 mice per group). (H) Representative waveforms of calcium transient alterations at various pacing cycle lengths (150–90 ms) in the four groups induced by a gradual decrease in pacing cycle length via electrical stimulation (n = 6 mice per group). (I) Calculation of the calcium‐transient alternans ratios (CaT alternans ratios, top). Statistical plots of the mean values of the calcium transient alternans ratio for four groups at different cycle lengths (bottom, n = 6 mice per group). The results are presented as the mean ± SD. Data were analyzed using two‐way ANOVA followed by the Bonferroni post hoc test.

Next, we focused on whether the UCHL1–CaMKII‐δ axis influences RyR2‐mediated Ca^2+^ handling in mouse atria using optical mapping, which enables assessment of Ca^2+^ handling at the whole‐heart level in vivo. Compared with CTR mice, UCHL1‐CKO mice showed attenuation of the Ang II‐induced reduction in Ca^2+^ conduction velocity (CV), Ca^2+^ transient duration at 90% repolarization (CaD90) and prolongation of the time to peak (TTP), and increase in the Ca^2+^ transient (CaT) alternans ratio at 90–110 ms in the atria (Figure 6D, Figure S11A–C). Conversely, rAAV9‐UCHL1‐overexpressing mice markedly exacerbated Ang II‐induced changes in atrial Ca^2+^‐CV, TTP, CaT alternans ratio, and CaD90 compared with those in rAAV9‐EV‐injected controls (Figure 6E, Figure S11D–F). However, UCHL1 overexpression‐induced aggravation of the Ang II response was substantially ameliorated in mice co‐injected with rAAV9‐UCHL1 and rAAV9‐siCaMKII‐δ (Figure 6F–I). A similar beneficial effect was observed in mice co‐injected with rAAV9‐EV and rAAV9‐siCaMKII‐δ (Figure 6F–I).

To further validate the effect of UCHL1‐CaMKII‐δ pathway on Ca^2+^ handling abnormalities, we performed calcium spark and calcium transient analyses in cardiomyocytes isolated from mice injected with rAAV9‐UCHL1 and rAAV9‐siCaMKII‐δ under Ang II stress using fluorescence microscopy. In the resting state, UCHL1 overexpression significantly increased Ca^2+^ spark frequency (1.9‐fold; *p* = 0.0163; Figure S12A,B). Upon electrical stimulation, Fluo‐4 fluorescence was normalized to baseline (F0) to assess Ca^2+^ transient properties. Compared with controls, UCHL1 overexpression significantly decreased Ca^2+^ transient amplitude (0.6‐fold; *p* = 0.019; Figure S12C,D) and slowed Ca^2+^ transient decay kinetics, as reflected by a significant increase in Tau (0.312 ± 0.05 s vs. 0.255 ± 0.04 s, *p* = 0.0014) (Figure S12E,F). However, UCHL1 overexpressing‐dependent Ca^2+^ abnormalities were reversed in CaMKII‐δ‐knockdown cardiomyocytes. Taken together, these findings define a novel role for UCHL1 in reinforcing Ang II‐induced abnormality of Ca^2+^ handling through activation of CaMKII‐δ–RyR2 pathway.

### 2.7 UCHL1‐CaMKII‐δ Axis Enhances AF Inducibility

To elucidate whether UCHL1 drives AF susceptibility by stabilizing CaMKII‐δ, WT mice were injected with rAAV9‐EV (empty vector) or rAAV9‐UCHL1 together with either rAAV9‐siControl or rAAV9‐siCaMKII‐δ for 2 weeks prior to Ang II infusion (Figure 7A). Immunoblotting revealed that exogenous Flag‐tagged UCHL1 was highly expressed, and endogenous CaMKII‐δ and p‐RyR2 protein levels were reduced by approximately 50% in mouse atrial tissue (Figure 6C).

**FIGURE 7:**
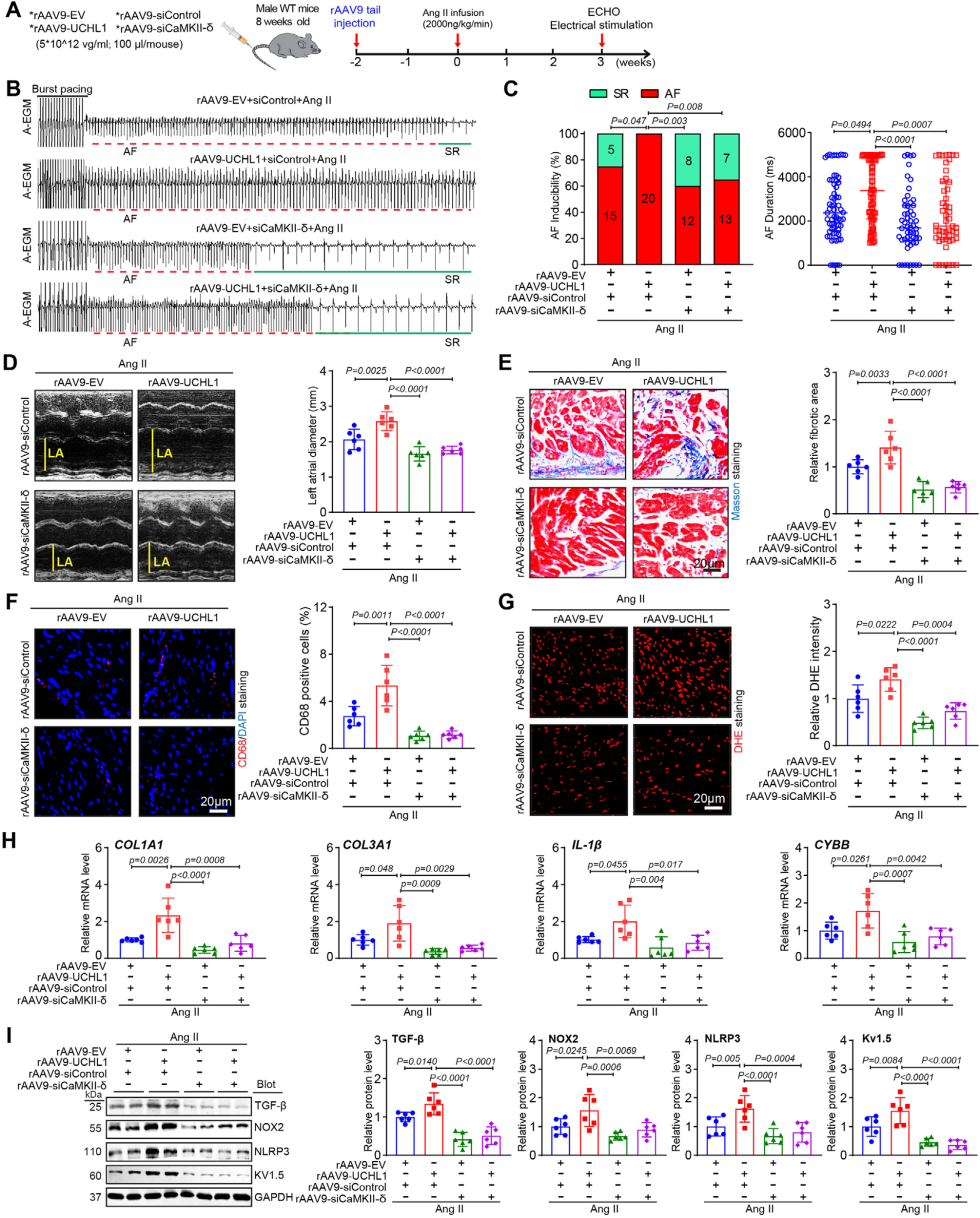
UCHL1 aggravates Ang II‐induced AF by stabilizing CaMKII‐δ. (A) Schematic illustration for rAAV9 injection and AF mouse model generation. WT mice (C57BL/6J, 8‐week‐old male) were injected with rAAV9‐EV or rAAV9‐UCHL1 (under the cardiac‐specific cTNT promoter) together with rAAV9‐siControl or rAAV9‐siCaMKII‐δ (under the cardiac‐specific cTNT promoter) for 2 weeks and then treated with Ang II for another 3 weeks. (B) Representative images of intracardiac electrogram recordings after burst pacing. Red dotted line: AF; green solid line: SR. (C) Percentage of successful AF induction (top, n = 20 mice per group) and quantitative analysis of total AF duration (bottom, n = 20 mice per group). (D) Representative images of M‐mode echocardiography (left) and quantification (right) of left atrial diameter (LAD, mm; n = 6 mice per group). (E) Representative images and quantification of Masson's staining in left atria (n = 6 mice per group). (F) Immunofluorescent staining (left) and quantification (right) of CD68‐positive macrophages in left atria (n = 6 mice per group). (G) DHE staining (left) and quantification of superoxide levels (right) in atrial tissues (n = 6 mice per group). (H) qPCR analysis of the mRNA levels of *COL1A1, COL3A1, IL‐1β*, and *CYBB* (encoding NOX2) in atria (n = 6 mice per group). (I) Representative immunoblots (left) and quantification (right) of TGF‐β, NOX2, NLRP3, and Kv1.5 in atria from four groups (n = 6 mice per group). The results are presented as mean ± SD. Data in C were analyzed using Fisher's exact test for fraction of successful AF induction, and Mann–Whitney U tests followed by Dunn's multiple comparison test for duration of AF. Data presented in D–I were analyzed using two‐way ANOVA followed by the Bonferroni post hoc test.

Compared with rAAV9‐EV‐injected mice, rAAV9‐UCHL1–overexpressing mice displayed marked increases in Ang II‐induced AF inducibility, AF duration, and LAD. These effects were largely mitigated in rAAV9‐siCaMKII‐δ–injected mice (Figure 7B–D). Next, we examined the impact of CaMKII‐δ knockdown on atrial fibrosis, inflammation, and oxidative stress in UCHL1‐overexpressing mice. As expected, UCHL1 overexpression‐dependent increases in atrial fibrotic area, numbers of CD68‐positive macrophages, superoxide levels, and the mRNA levels of *COL1A1, COL3A1, IL‐1β*, and CYBB/NOX2, as well as protein levels of TGF‐β, NOX2, NLRP3, and Kv1.5, were all attenuated in mice injected with rAAV9‐siCaMKII‐δ (Figure 7E–I). Similar protective effects of rAAV9‐siCaMKII‐δ injection were also observed in rAAV9‐EV‐injected mice (Figure 7B–I). Thus, these data support the notion that UCHL1‐mediated CaMKII‐δ stabilization confers susceptibility to AF.

To further consolidate the protective role of CaMKII‐δ inhibition in Ang II‐induced AF in UCHL1‐overexpressing mice, rAAV9‐EV‐ or rAAV9‐UCHL1‐injected WT mice were pretreated with the CaMKII inhibitor KN93 and then infused with Ang II for 3 weeks. Overexpression of UCHL1 markedly increased CaMKII‐δ protein levels, whereas administration of KN93 did not affect the expression of UCHL1 or CaMKII‐δ in mouse atrial tissues (Figure S13A,B). Moreover, UCHL1 overexpression accelerated Ang II‐induced increases in AF inducibility and duration, LAD enlargement, atrial fibrosis, superoxide production, and mRNA levels of *COL1A1, COL3A1, IL‐1β, CYBB* (NOX2), and *NOX4*. These effects were attenuated in both rAAV9‐EV‐ and rAAV9‐UCHL1‐injected mice after KN93 treatment (Figure S13C–H). Overall, these data demonstrate that UCHL1 mediates susceptibility to AF by promoting CaMKII‐δ activation.

### 2.8 Pharmacological Inhibition of UCHL1 Attenuates Susceptibility to AF

The results above demonstrate that genetic deletion of UCHL1 suppressed AF inducibility (Figure 2), and our previous study also indicated that inhibition of UCHL1 with LDN‐57444, a reversible competitive inhibitor, exerts an antiarrhythmic effect [32], suggesting that UCHL1 could be a potential target for AF. 6RK73 is a newly discovered irreversible and specific UCHL1 inhibitor [33], but its role in AF remains unclear. This prompted us to evaluate whether 6RK73 has a similar effect in an Ang II‐induced murine AF model. WT mice were administered with 6RK73 (0.5 mg/kg, three times weekly) starting 2 days before Ang II infusion, which was continued for 3 weeks (Figure 8A,B). Of note, preliminary safety assessment showed that 6RK73 treatment did not significantly alter left ventricular ejection fraction (EF%) or serum lactate dehydrogenase (LDH) levels (Figure S14A,B), suggesting favorable cardiac tolerability at the current dose. The results revealed that administration of 6RK73 markedly reduced the AF incidence and duration, LAD, atrial fibrosis, CD68‐positive macrophage infiltration, and superoxide production in Ang II‐infused mice compared with those in DMSO‐treated controls (Figure 8C–H). Similar effects were observed at the mRNA level. qPCR analysis indicated that 6RK73 markedly attenuated Ang II‐induced upregulation of *COL1A1, COL3A1, IL‐1β, IL‐6, CYBB/NOX2*, and *NOX4* in atrial tissues of mice (Figure 8I). Consistently, 6RK73 administration also significantly lowered protein levels of CaMKII‐δ, p‐RyR2, TGF‐β, NOX2, NLRP3, and Kv1.5 in atrial tissues of Ang II‐infused mice (Figure 8J). Collectively, these findings demonstrate that pharmacological inhibition of UCHL1 effectively prevents Ang II‐induced AF in mice, and highlight that UCHL1 has the potential to be a new target for AF therapy.

**FIGURE 8:**
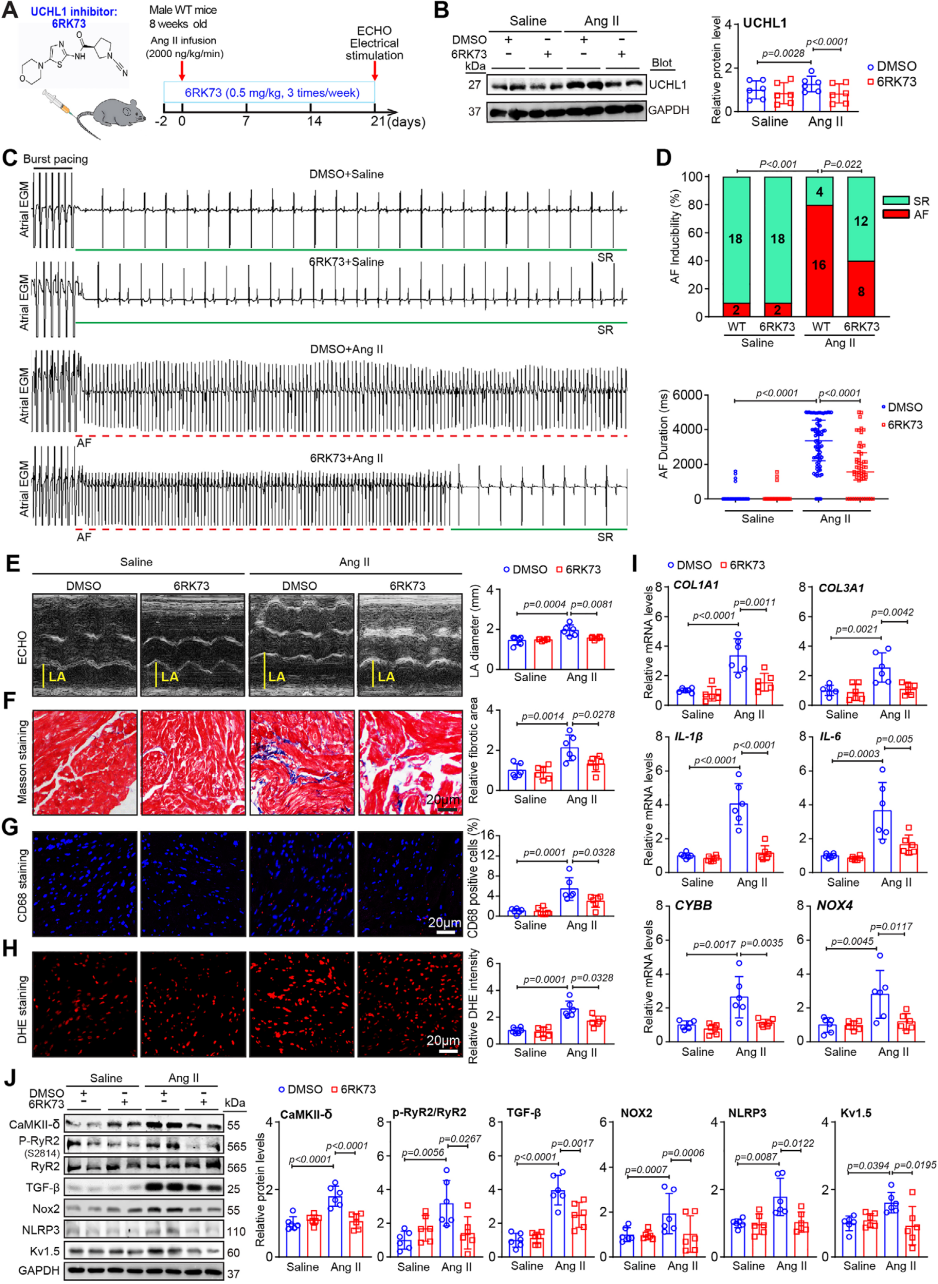
Administration of 6RK73 suppresses angiotensin II‐induced AF. (A) Experimental scheme of drug treatment in WT (C57BL/6J, 8‐week‐old male) mice treated with 6RK73, a covalent irreversible and specific inhibitor of UCHL1 (0.5 mg/kg, three times per week) for 2 days, and then infused with Ang II (2000 ng/kg/min) and 6RK73 for 3 weeks. (B) Representative immunoblots and quantification of UCHL1 protein levels using anti‐UCHL1 and anti‐GAPDH antibodies (n = 6 mice per group). (C) Representative intracardiac atrial electrogram recordings of mice treated as in A and subjected to burst pacing. Red dotted lines represent AF, and green solid lines represent sinus rhythm (SR). (D) Percentage of successful AF induction (top, n = 20 mice per group) and the duration of AF (bottom). (E) Representative M‐mode echocardiography (ECHO) images of LAD (mm) in the left atrium (n = 6 mice per group). (F–H) Representative histological images (left) and quantification (right, n = 6 mice per group), including Masson's staining for fibrosis (blue), IF staining of CD68‐positive macrophages (red for CD68 staining, blue for DAPI), and DHE staining (red for ROS) in 6RK73‐ or DMSO‐treated mice infused with saline or Ang II, as indicated. (I) qPCR analysis of the mRNA levels of *COL1A1*, *COL3A1*, *IL‐1β, IL‐6*, and *CYBB* (NOX2), and *NOX4* in atrial tissues (n = 6 mice per group). (J) Representative immunoblots of CaMKII‐δ, total and p‐RyR2 (S2814), TGF‐β, NOX2, NLRP3, and Kv1.5 protein levels (n = 6 mice per group). The results are presented as the mean ± SD. Data presented in B and E–J were analyzed using two‐way ANOVA followed by the Bonferroni post hoc test. Data in D were analyzed using Fisher's exact test for the proportion of successful AF induction, and Mann–Whitney U tests followed by Dunn's multiple‐comparison test for duration of AF.

## 3 Discussion

The current study revealed a critical role of UCHL1 in promoting AF inducibility by deubiquitinating and stabilizing CaMKII‐δ both in vitro and in vivo. The major findings are as follows: (i) UCHL1 levels were significantly upregulated in the atria of mice and patients with AF; (ii) atrial myocyte‐specific knockout of UCHL1 attenuated Ang II‐induced atrial remodeling and AF susceptibility, whereas UCHL1 overexpression aggravated these effects, establishing UCHL1 as a pivotal driver of AF pathogenesis; (iii) UCHL1 directly interacted with and stabilized CaMKII‐δ by cleaving K48‐linked polyubiquitin chains, which induced RyR2 activation and Ca^2+^ mishandling, thereby leading to AF; and (iv) administration of the UCHL1 inhibitor 6RK73 effectively reduced Ang II‐induced AF. Collectively, these observations identify a novel post‐translational mechanism by which UCHL1 mediates CaMKII‐δ deubiquitination and highlight UCHL1 as a promising therapeutic target for AF. A working model is shown in Figure S15.

DUBs are counter‐regulatory enzymes in ubiquitination, preventing protein degradation by the proteasome [34]. Human DUBs are classified into two main groups: cysteine proteases and zinc metalloproteases. They regulate protein stability, localization, and function, affecting virtually all areas of cellular physiology and biology. Several DUBs, including USP25, USP28, USP9X, USP7, USP38, and OTUD1, are key regulators of cardiovascular diseases by deubiquitinating and stabilizing SR Ca^2+^‐ATPase 2A (SERCA2a), peroxisome proliferator‐activated receptor alpha (PPARα), signal transducer and activator of transcription 3 (STAT3), and scavenger receptor‐A1 (SR‐A1) [35, 36, 37, 38, 39, 40]. Notably, USP7 and USP38 promote susceptibility to AF in mouse models through moulation of the NF‐κB and iron‐regulatory protein 2 (IRP2) pathways [24, 26, 41, 42], implying that DUBs can modulate AF. UCHL1, a member of the ubiquitin C‐terminal hydrolase family, is markedly elevated primarily in cardiac myocytes, following MI, Ang II infusion, or pressure overload, indicating that cardiomyocytes, in addition to neural tissue, are a primary source of UCHL1 under stress [16, 43, 44]. UCHL1 deubiquitinates diverse protein substrates and plays critical roles in several cardiovascular diseases, including PAH, vascular remodeling, hypertension, atherosclerosis, MI, I/RI, cardiac hypertrophy, and AF [15, 16, 17, 18, 19, 20, 21, 22, 23, 45]. UCHL1 deficiency attenuates aortic banding‐induced cardiac hypertrophy by increasing EGFR ubiquitination and degradation [16]. Deletion of UCHL1 limits post‐MI cardiac fibrosis via deubiquitination of 78‐kDa glucose‐regulated protein (GRP78) and hypoxia inducible factor‐1α (HIF‐1α) [17, 18, 43]. More recently, a study demonstrated that UCHL1 knockout attenuates PAH in different animal models by increasing AKT1 deubiquitination and AKT1 protein levels [15]. Although our earlier study showed that pharmacological inhibition of UCHL1 with LDN‐57444 confers a beneficial effect on Ang II‐induced AF [32], the precise role and underlying mechanism of UCHL1 in AF pathogenesis remain largely unexplored. In this study, we found that UCHL1 expression was highly upregulated in the atria of mice and patients with AF (Figure 1). Atrial cardiomyocyte‐specific knockdown of UCHL1 substantially inhibited atrial remodeling and AF susceptibility (Figure 2), whereas UCHL1 overexpression accelerated Ang II‐induced proarrhythmic effects (Figure 3). Notably, we confirmed that the proarrhythmic function of UCHL1 is dependent on its enzymatic activity (Figure S9). These findings extend the known roles of UCHL1 in other diseases and demonstrate that UCHL1 also exerts a proarrhythmic effect in hypertensive mice. However, the regulatory mechanism by which hypertension or Ang II upregulates UCHL1 expression remains to be explored.

Atrial arrhythmogenic remodeling involves fibrosis, altered Ca^2+^ currents, and abnormal ion channel function, which frequently predispose to AF. Ca^2+^ handling abnormalities, oxidative stress, and inflammatory signaling are key molecular drivers of AF initiation and progression. CaMKII, a multifunctional serine/threonine kinase with four isoforms (α, β, γ, and δ), is highly expressed in the myocardium. CaMKII plays a critical role in cardiovascular diseases, including myocardial I/RI, MI, cardiac hypertrophy, sinus node dysfunction, and arrhythmias, by regulating proteins involved in Ca^2+^ homeostasis, cell death, inflammation, oxidative stress, and fibrosis [46, 47, 48, 49]. CaMKII is a well‐established inducer of AF through phosphorylation of RyR2 at S2814 and disruption of Ca^2+^ handling in the atria [50, 51, 52]. Activation of the NLRP3 inflammasome, CaMKII‐dependent RyR2 phosphorylation, and Ca^2^^+^ handling abnormalities are observed in atrial tissues and cardiomyocytes from patients who develop postoperative AF [53]. Conversely, pharmacological inhibition or genetic deletion of CaMKII corrects intracellular Ca^2+^ mishandling and prevents AF initiation and progression [4, 31]. Thus, CaMKII is a central mediator of atrial remodeling and AF.

Moreover, CaMKII functions as a critical sensor for Ca^2+^, ROS, and inflammatory stress, mediating activation of NF‐κB and the NOD‐like pyrin domain‐containing protein 3 (NLRP3) inflammasome. This leads to upregulation of monocyte chemoattractant protein‐1 (MCP1) and IL‐1β, promoting macrophage infiltration and subsequent cardiac fibrosis and dysfunction induced by Ang II or pressure overload [54, 55, 56]. Notably, UCHL1 has been identified as a regulator of NLRP3, TGF‐β, and NOX signaling through different mechanisms in noncardiomyocytes [57]. UCHL1 activity supports DAF‐7/TGF‐β signaling, and its deubiquitination function has been proposed as a potential therapeutic target in lung cancer [58, 59]. However, whether UCHL1 promotes AF via profibrotic, proinflammatory, and prooxidative signaling through the CaMKII‐RyR2 axis remains unclear.

In this study, knockdown of UCHL1 in atrial cardiomyocytes significantly attenuated Ang II‐induced atrial fibrosis, macrophage infiltration, and ROS production, accompanied by downregulation of CaMKII‐δ, p‐RyR2, TGF‐β, NOX2, NLRP3, and Kv1.5 protein levels (Figure 2). Conversely, these proarrhythmic effects were exacerbated by UCHL1 overexpression (Figure 3), but were mitigated by CaMKII‐δ knockdown or inhibition in mice (Figure 6). Therefore, UCHL1‐mediated stability of CaMKII‐δ is a critical mechanism by which Ang II or hypertensive signaling induce activates the TGF‐β, NLRP3, and NOX2 pathways, promoting atrial remodeling and AF.

CaMKII expression and activity are regulated at multiple levels, including epigenetic, transcriptional, and post‐transcriptional mechanisms. Long non‐coding RNA terminal differentiation‐induced non‐coding RNA epigenetically reduces CaMKII expression during myocardial hypertrophy [60], whereas the transcription factor ΔFosB binds the CaMKII‐α gene promoter to increase CaMKII expression in the nucleus accumbens [61]. Notably, several PTMs, including phosphorylation, oxidation, and O‐GlcNAcylation, effectively modulate CaMKII activity [52, 62, 63]. High glucose levels promote O‐GlcNAcylation of CaMKII‐δ at S280, which is required for cardiomyocyte ROS production, abnormal SR Ca^2+^ release, and subsequent arrhythmia [62]. Moreover, Ang II infusion enhances ROS generation, increasing oxidized CaMKII‐δ levels in the atria of mice and patients with AF. In contrast, mutation of the oxidation sites at methionines 281 and 282 in CaMKII‐δ reduces Ang II‐induced AF, indicating that oxidized CaMKII‐δ is an important driver of AF [64]. However, whether UCHL1 regulates CaMKII‐δ stability via deubiquitination in AF has not been defined.

In this study, we performed Co‐IP combined with LC‐MS/MS to identify CaMKII‐δ as a critical target of UCHL1 in ACMs. Importantly, UCHL1 removed K48‐linked polyubiquitin chains from CaMKII‐δ at K251, thereby increasing CaMKII‐δ stability, which resulted in RyR2 activation and abnormal Ca^2+^ handling, ultimately contributing to atrial remodeling and AF inducibility. Consistently, compared with rAAV9‐CaMKII‐δ‐WT injection, overexpression of the rAAV9‐CaMKII‐δ‐K251R mutant in UCHL1‐CKO mice exacerbated Ang II‐induced atrial remodeling (Figure S10). Conversely, knockdown of CaMKII‐δ or pharmacological inhibition of its activity attenuated UCHL1 overexpression‐mediated activation of RyR2, Ca^2+^ handling abnormalities, and proarrhythmic effects in mice. In addition to the UCHL1‐regulated CaMKII‐δ‐RyR2 pathway, other factors, including AKT, NLRP3, NOXs, and interleukin‐related inflammatory mechanisms, may also contribute to atrial remodeling and AF [15, 57]. Collectively, these results reveal a previously unknown mechanism for the UCHL1‐CaMKII‐δ axis underlying Ang II‐induced AF, and provide a potential therapeutic target for AF therapy.

AF continues to cause high cardiovascular morbidity and mortality worldwide, and current therapeutic approaches, including pharmacological, gene‐based, and ablative techniques, have limited efficacy and may produce adverse effects [65]. Therefore, targeting DUBs represents a potential therapeutic strategy for treating AF. Several DUBs, such as USP38 and USP7, have gained attention as targets in AF. Inhibition of USP38 is beneficial against AF induced by pressure overload, MI, and diabetes mellitus [26, 41, 42]. Our previous studies also revealed that blocking USP7 or UCHL1 with p22077 or LDN‐57444 substantially attenuates Ang II‐induced AF in mice [24, 32]. However, no clinically approved DUB inhibitors are currently available. 6RK73, a novel covalent irreversible UCHL1 inhibitor, has recently been investigated in breast and renal cancers [33]. Here, we extend the evaluation of 6RK73 and show that its administration in mice effectively prevents Ang II‐induced AF (Figure 8), further confirming that 6RK73 could be a promising therapeutic candidate for AF, although comprehensive safety and cardiotoxicity assessments are required before any clinical translation.

This study has some limitations. First, the Ang II‐induced AF model primarily reflects neurohormonal activation and fibrotic drivers, rather than the complete pathophysiology of spontaneous or long‐standing persistent AF. Thus, this model may not fully recapitulate the diverse clinical substrates of AF (e.g., genetic, postoperative, or vagal‐dependent mechanisms). Second, the tissue samples were obtained from a specific surgical population (e.g., patients with valvular heart disease or those undergoing standalone AF ablation), which may introduce selection bias. Third, although we adjusted for clinical confounding factors to the best of our ability, residual confounding from patient comorbidities and the use of antiarrhythmic or anticoagulant agents may still affect the observed association between UCHL1 expression and AF. Fourth, given the expression of UCHL1 in the brain and other organs, possible extracardiac risks of its inhibition warrant future tissue‐specific investigations. Fifth, although our results suggest that UCHL1 mediates CaMKII‐δ deubiquitination, RyR2 activation, and Ca^2+^ handling abnormalities, additional downstream targets, and key signaling pathways remain to be explored.

## 4 Conclusion

Our data reveal a novel mechanistic link between UCHL1 and CaMKII‐δ in AF pathogenesis. To the best of our knowledge, we identified UCHL1 as a previously unrecognized deubiquitinase that enhances CaMKII‐δ stability by cleaving K48‐linked polyubiquitin chains from CaMKII‐δ at K251, thereby resulting in RyR2 activation and Ca^2+^ mishandling in the atrial myocytes and promoting AF inducibility. Pharmacological inhibition of UCHL1 with 6RK73 attenuated AF development, providing a potential therapeutic strategy for the treatment of AF.

## 5 Experimental Section/Methods

All studies involving human tissues strictly abided by the principles outlined in the World Medical Association Declaration of Helsinki and were approved by the ethics committee of the First Affiliated Hospital of Dalian Medical University (PJ‐KS‐KY‐2021‐229, to X‐HY). Informed consent was obtained from all participants. Animal experiments were approved by the Committee on Ethics of Animal Experiments of Dalian Medical University and complied with the National Institutes of Health Guide for the Care and Use of Laboratory Animals.

### 5.1 Patients and Heart Samples

Human atrial tissues were obtained from patients with AF to assess UCHL1 expression. One cohort included six patients with rheumatic mitral valve disease undergoing mitral valve surgery at the First Affiliated Hospital of Dalian Medical University, China. Patients were classified based on the presence of persistent AF: the chronic AF group (n = 3) with documented arrhythmia lasting over 6 months before surgery, and the normal SR group (n = 3) without a history of AF on previous examinations or at admission (Table S1). A second cohort included eight patients with coronary artery disease from the same hospital. Using the same criteria, patients were divided into chronic AF (n = 4) and SR (n = 4) groups (Table S1). All patients underwent routine preoperative two‐dimensional color Doppler echocardiography. Patients with heart failure (New York Heart Association class ≥ III), sick sinus syndrome, pulmonary artery disease, renal disease, or secondary thoracotomy were excluded. Left atrial appendages were collected before extracorporeal circulation and radiofrequency ablation. A portion of the tissue was fixed in 10% formalin for histology, while the remainder was snap‐frozen in liquid nitrogen and stored at −80°C for immunoblotting, transcriptomic analysis, and qPCR analysis.

Participants providing human serum samples were recruited at the First Affiliated Hospital of Dalian Medical University (Dalian, China). Initially, 103 AF patients and 103 SR controls were enrolled. After applying pre‐defined exclusion criteria (missing key clinical covariates, insufficient serum volume, or hemolyzed samples), the final analytical cohort comprised 82 AF patients and 50 SR controls. AF was diagnosed based on electrocardiogram characteristics: (1) irregular R‐R intervals when atrioventricular conduction is present; (2) absence of distinct repeating P waves replaced by fibrillation waves (“f waves”) of variable size, morphology, and interval; and (3) irregular atrial activity in accordance with the European Society of Cardiology/American College of Cardiology guidelines [66]. Detailed patient characteristics are shown in Table S2. Serum UCHL1 levels were measured using an ELISA kit.

### 5.2 Human UCHL1 ELISA

UCHL1 levels were measured using an ELISA kit (#DY6007‐05, R&D Systems, Minneapolis, MN, USA) with the optional DuoSet Ancillary Reagent Kit 2 (catalogue no. DY008), following the manufacturer's instructions. Anti‐human UCHL1 capture antibody (DY6007, 1:120; R&D Systems) was diluted in 0.2 m sodium bicarbonate (pH 9.4) was coated in 96‐well plates (100 µL/well) overnight at 4°C. After coating, the plates were washed three times with 0.05% Tween 20 in PBS (PBS‐T) and blocked with 5% BSA in PBS overnight at 4°C. Serum samples (100 µL) or UCHL1 standards (prepared in Reagent Diluent) were added and incubated at 21–23°C for 2 h, followed by three washes with PBS‐T. After three washes, the plates were incubated with biotinylated sheep anti‐human UCHL1 detection antibody (DY6007, 1:60, R&D Systems) at room temperature for 2 h. Next, the plates were incubated with streptavidin‐HRP‐biotin (DY6007, 1:40; R&D Systems) at room temperature for 30 min, and then washed three times with PBS‐T. Ultra TMB‐ELISA substrate (#34028; Thermo Fisher Scientific) was used according to the manufacturer's protocol to detect the bound proteins, and the signals were analyzed using a Promega plate reader at 450 nm. The standard curve for UCHL1 concentrations was determined using 0.078–2.5 ng/µL recombinant human UCHL1 (DY6007, R&D Systems).

### 5.3 Animals

*Uchl1^flox/+^* (*Uchl1^fl/+^*) mice and atrial cardiomyocyte‐specific *Nppa*‐Cre mice were used to generate atrial cardiomyocyte‐specific UCHL1 knockout mice (UCHL1‐CKO, *Uchl1* ^fl/fl^/*Nppa*‐*Cre* ^+^) and littermate controls (UCHL1‐CTR, *Uchl1^fl/fl^*/*Nppa‐Cre* ^−^). C57BL/6J WT and *Uchl1* ^+^/^−^ mice were obtained from Jackson Laboratory. Mice were housed under specific pathogen‐free conditions (24°C, 40–60% humidity, 12‐h light/dark cycle).

### 5.4 AF Induction and Duration in Mice

AF induction and duration were assessed as previously described [32]. The 11–13‐week‐old mice were anaesthetized with 2.5% tribromoethanol (Sigma–Aldrich) via intraperitoneal injection. A Millar 1.9 F octapolar electrophysiology catheter (Scisense, London, Ontario, Canada) was inserted through the right jugular vein into the right atrium. Atrial arrhythmias were induced using an automated multichannel electrophysiology stimulating/recording system (GY‐66328B, HuaNan Medical Science & Technology Ltd.). Thirty‐three 5‐s tachypacing bursts were applied at three voltages (3.5, 5, and 8 V) and decreasing cycle lengths (from 40 to 20 ms in 2‐ms steps) for each mouse by an investigator blinded to group assignment. AF was defined as atrial rates >1500 beats/min producing rapid, irregular atrial waves lasting ≥1 s.

### 5.5 High‐Resolution Optical Mapping

Mice were intraperitoneally injected with heparin (1000 IU/ml, 0.2 ml) and euthanized after 15 min. Hearts were excised and perfused on a Langendorff apparatus with K‐H buffer containing blebbistatin (6.84 µm), pluronic F127, and Rhod‐2 AM. Excitation light was provided by LED sources, and intracellular Ca^2^^+^ signal emission was filtered with a 590 nm bandpass filter. High‐speed Complementary Metal‐Oxide‐Semiconductor (CMOS) cameras (0.9 kHz frame rate) and the OMapScope software (v5.9.45) were used to acquire images and analyze calcium transient parameters (conduction velocity, time to peak, CaD90, and amplitude).

### 5.6 Isolation of Atrial Myocytes From Neonatal Rats

Neonatal rat atrial cardiomyocytes (NRACMs) and atrial cardiac fibroblasts (NRACFs) were enzymatically isolated from the atrial tissues of 1–3‐day‐old Sprague–Dawley rat hearts. Sprague–Dawley rat hearts were quickly removed and washed thrice with ice‐cold PBS to remove blood. Atria were separated from ventricles, cut into pieces, and dissociated in 0.04% trypsin and 0.07% type II collagenase at 37°C, as previously described [24]. The cell suspension was filtered through a 100 µm cell strainer, centrifuged at 300 × g for 10 min, and resuspended in Dulbecco's modified Eagle's medium (DMEM)/F12 (Gibco, USA) containing 20% FBS (Invitrogen) and 1% penicillin/streptomycin (P/S, Invitrogen). Cells were incubated in 100 mm plastic dishes at 37°C with 5% CO_2_ for 2 h. Atrial cardiomyocytes in the supernatant were separated from atrial fibroblasts, which had adhered to plastic dishes. Subsequently, the cells were seeded onto glass coverslips or different cell culture dishes, incubated in culture medium and 100 µm BrdU for 36 h, and used for subsequent experiments.

### 5.7 Cell Lines and Cell Culture

Embryonic kidney 293T (HEK293T) cells were obtained from the American Type Culture Collection (ATCC, Manassas, VA, USA), and were cultured in DMEM (Hyclone) supplemented with 10% FBS (Invitrogen) and 1% P/S (Invitrogen).

### 5.8 Plasmids Transfection and Adenoviral Infection

Plasmids expressing WT or various mutants of UCHL1 (mutant C90S, and truncations ΔN, ΔC, and Δ90 to 175) were prepared as described previously [16]. HA‐ubiquitin (Ub) (WT, and mutants K48R, and K63R), pcDNA3.1‐Myc‐hCaMKIID WT, and truncations (1–301, 1–291, 1–282 aa) were constructed by HedgehogBio Co. Ltd. (Shanghai, China). The plasmids were transfected with Lipofectamine 2000 (Lipo2000, Invitrogen) as described previously [16]. HEK293T cells were incubated in fresh FBS‐free medium. Plasmids and Lipo2000 were separately mixed with Opti‐MEM (Gibco; Invitrogen) for 5 min. The mixtures were combined, incubated for 20 min at room temperature, added dropwise to the cells, and incubated at 37°C for 6 h. Fresh medium containing FBS was then added, and cells were maintained at 37°C in a culture incubator until use.

For adenoviral infection, adenovirus siRNA‐control, siRNA‐UCHL1, Ad‐GFP, or Ad‐UCHL1 (Hanbio Biotechnology; Shanghai, China) [multiplicity of infection = 50] was performed as described previously [16].

### 5.9 Quantitative Real‐Time (qRT)‐PCR Analysis

qRT‐PCR was performed as reported previously [16]. Total RNA from cells and fresh atrial tissues was extracted using TRIzol reagent (Invitrogen/ Thermo Fisher Scientific). The first‐strand cDNA was synthesized using random primers/Oligo (dT)‐primer mix RT kits (11141ES60; Yeasen, Shanghai, China). Quantitative PCR was performed using SYBR Green kits (RR820A; Takara Bio Inc. Shiga, Japan) on an Applied Biosystems 7500 Real Time PCR System (Applied Biosystems). The detected mRNA levels were normalized to those of endogenous control *GAPDH*. The sequences of the qPCR primers (purchased from Sangon Biotech; Shanghai, China) are shown in Table S4.

### 5.10 Western Blotting

Atrial tissues or cells were lysed with RIPA lysis buffer containing 1 mM PMSF (Solarbio, Beijing, China) and 0.1% cocktail (Roche, Basel, Switzerland), as described previously [16]. Homogenates were lysed for 30 min on ice and centrifuged at 17 000 × g and 4°C for 15 min. The supernatant was collected and boiled for 5 min. Proteins were separated via 8–12% SDS‐PAGE and transferred onto a PVDF membrane (ISEQ00010, Millipore, Shanghai, China). Primary and secondary antibodies were used to probe for multiple proteins listed in Table S5. The whole blot was imaged and analyzed with a near‐infrared imaging system (Odyssey CLx; LI‐COR Biosciences, Lincoln, NE).

### 5.11 Immunoprecipitation (IP) and Co‐immunoprecipitation (Co‐IP) and Ubiquitylation Assays

IP was performed as described previously [16]. NRACMs, HEK293T cells, or atrial tissues were lysed in IP‐TNE buffer (50 mM Tris‐HCl, 150 mm NaCl, 0.25% Sodium deoxycholate, 1 mm EDTA, 0.5% NP‐40, pH 7.4) with PMSF (Solarbio) and 0.1% cocktail (Roche, Basel, Switzerland) on ice for 40 min. Cell or tissue extracts were collected after centrifugation at 17 × g and 4°C for 15 min. Standard procedures (BCA; Thermo Fisher Scientific) were used for immunoprecipitation. The supernatant was incubated with the appropriate antibody and the Protein A/G Magnetic Beads (B23202; Selleck Chem, USA) at 4°C overnight. The magnetic beads were centrifuged and washed twice according to the manufacturer's recommendations. The bound proteins were subjected to LC‐MS/MS or SDS‐PAGE using 8% or 10% gels followed by western blotting. The detailed information of the antibodies is listed in Table S5. For ubiquitylation assays, procedures were performed as previously described [16]. To assess endogenous CaMKII ubiquitination, atrial tissues, or HEK293T cells were infected with appropriate adenovirus or plasmids for 24 h and treated with MG132 for 6 h before harvest. Cells and tissues were lysed with IP‐TNE buffer, and standard procedures were followed thereafter as previously described [16]. CaMKII was immunoprecipitated with anti‐CaMKII, anti‐Myc, or anti‐Flag antibodies. The mixture was detected using western blotting and analyzed with appropriate antibodies (e.g., CaMKII, Ub, HA, Flag, and Myc; listed in Table S5).

### 5.12 Histological Analysis

The atrial tissues were fixed in 4% paraformaldehyde solution for at least 24 h, then embedded in paraffin. The sections (4 µm) were stained using a Masson Staining Kit (DC0032, Leagene, Beijing, China) according to the standard procedure. For the IHC assay, the heart slides were deparaffinized, and antigen retrieval was performed with the citrate buffer. The sections were stained with appropriate antibodies (including UCHL1 and CD68) for overnight at 4°C. Digital photographs of over 10 random fields from each atrial tissue were taken at 200× magnification and analyzed using Image Pro Plus 3.0 (Nikon, Japan).

### 5.13 Immunofluorescence

Immunofluorescence was performed as described previously [16]. For NRACMs, the cells were fixed in 4% paraformaldehyde for 15 min and blocked in PBS containing 1% BSA and 0.2% Triton X‐100 for 10 min at 20–23°C. The primary antibodies against α‐actinin (1:200 dilution) and UCHL1 (1:100 dilution) were incubated overnight at 4°C. For atrial tissues, the sections were blocked in PBS, containing 1% BSA, 0.025% Triton X‐100, and 0.3 m glycine, for 60 min at 20–23°C and then stained with appropriate antibodies (including UCHL1, cTNT, and CD68) overnight at 4°C. The cells/sections were incubated with the secondary Alexa Fluor 488‐conjugated Goat Anti‐Mouse antibody (ab150113; Abcam) and/or the Alexa Fluor 555 Goat Anti‐Rabbit antibody (ab150078; Abcam) for 1 h at 20–23°C. The cells were then counterstained with DAPI, mounted, and observed via fluorescence microscopy (STELLARIS 5 Confocal Microscope, Leica, Germany). The fluorescence signal was detected using LAS X (v4.4.0). All experiments and analyses were performed in a blinded manner.

### 5.14 Statistical Analysis

Data are presented as mean ± standard deviation from independent experiments (sample sizes are specified in the figures and legends). Normality was assessed using the Shapiro–Wilk test. For normally distributed data, unpaired Student's t‐test (two groups) or a one‐/two‐way analysis of variance with the Bonferroni post hoc test (≥3 groups) was applied; non‐normal data were analyzed using the Mann–Whitney U test followed by Dunn's multiple comparison test. Fisher's exact test was used to test AF inducibility. Statistical significance was defined as *p <0.05*, and analyses were performed with GraphPad Prism 9.0 and Microsoft Excel 2020.

## Author Contributions

**Hai‐Lian Bi**: funding acquisition, writing – original draft, writing – review and editing, project administration, data curation, supervision, methodology, resources, visualization. **Yu‐Hui Gu**: data curation, methodology, validation, formal analysis. **Kai‐Wen Ren**: methodology, data curation, validation, formal analysis. **Xiao‐Hong Yu**: data curation, resources. **Yun‐Long Xia**: data curation, funding acquisition, resources. **Xiao‐Lei Yang**: writing – review and editing, writing – original draft, data curation, funding acquisition, supervision. **Hui‐Hua Li**: writing – original draft, writing – review and editing, validation, data curation, project administration, supervision, formal analysis, resources, visualization.

## Ethics Statement

All studies involving human tissues strictly abided by the principles outlined in the World Medical Association Declaration of Helsinki and were approved by the ethics committee of the First Affiliated Hospital of Dalian Medical University (PJ‐KS‐KY‐2021‐229, to X.‐H.Y.). Animal experiments were approved by the Committee on Ethics of Animal Experiments of Dalian Medical University and complied with the National Institutes of Health Guide for the Care and Use of Laboratory Animals.

## Consent Statement

Informed consent was obtained from all participants.

## Conflicts of Interest

The authors declare no conflicts of interest.

## Supporting information




**Supporting File 1**: advs77855‐sup‐0001‐SuppMat.docx.

**Supporting File 2**: advs77855‐sup‐0002‐SuppMat.pdf.

## Data Availability

The data that support the findings of this study are available on request from the corresponding author. The data are not publicly available due to privacy or ethical restrictions.
