# Supplementary material for: The Deubiquitinase UCHL1 Drives Susceptibility to Atrial Fibrillation by Stabilizing CaMKII‐δ
Source: Adv Sci (Weinh). 2026 Sep 29:e77855. Online ahead of print. doi: 10.1002/advs.77855 (PMC13624307; doi:10.1002/advs.77855)

Full unedited gel for Figure 1E

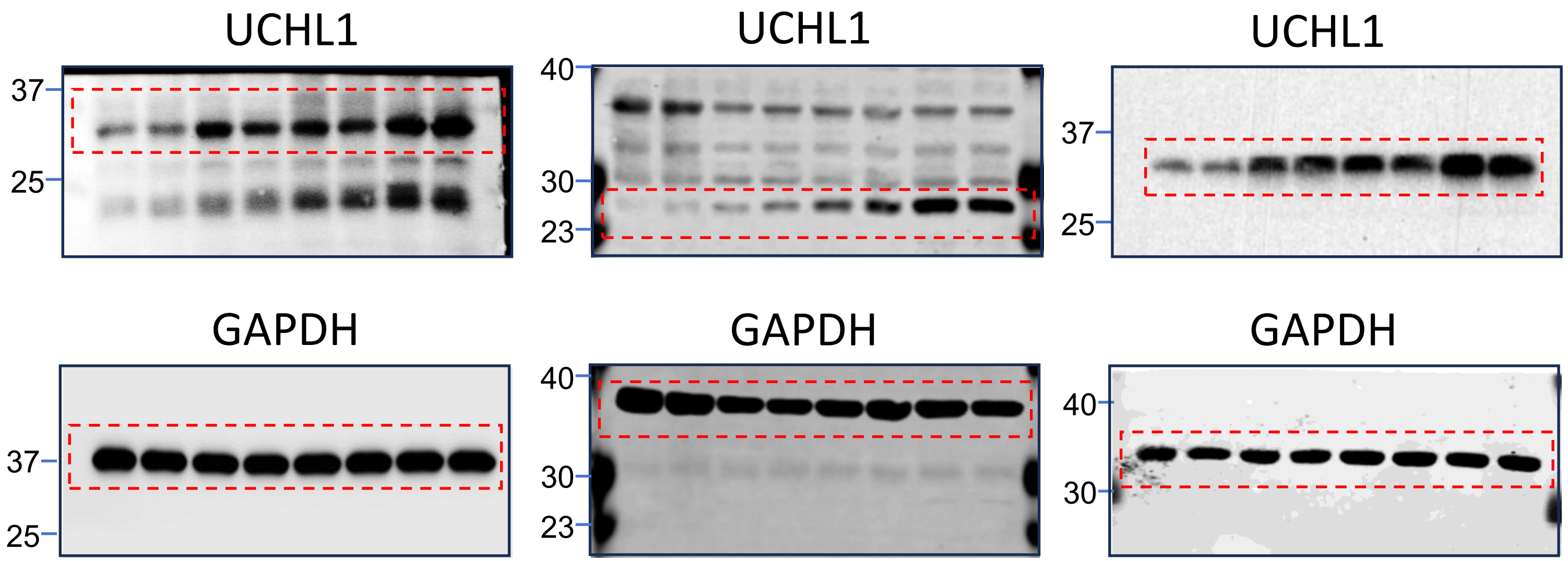

Full unedited gel for Figure 1F

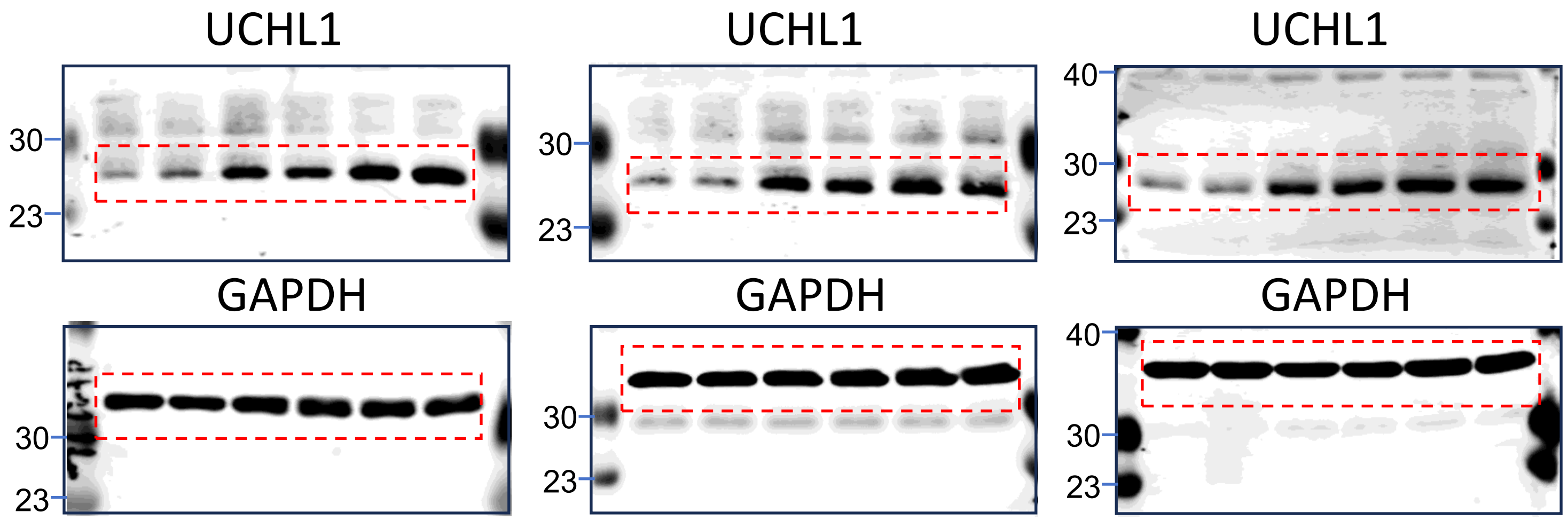

Full unedited gel for Figure 1K

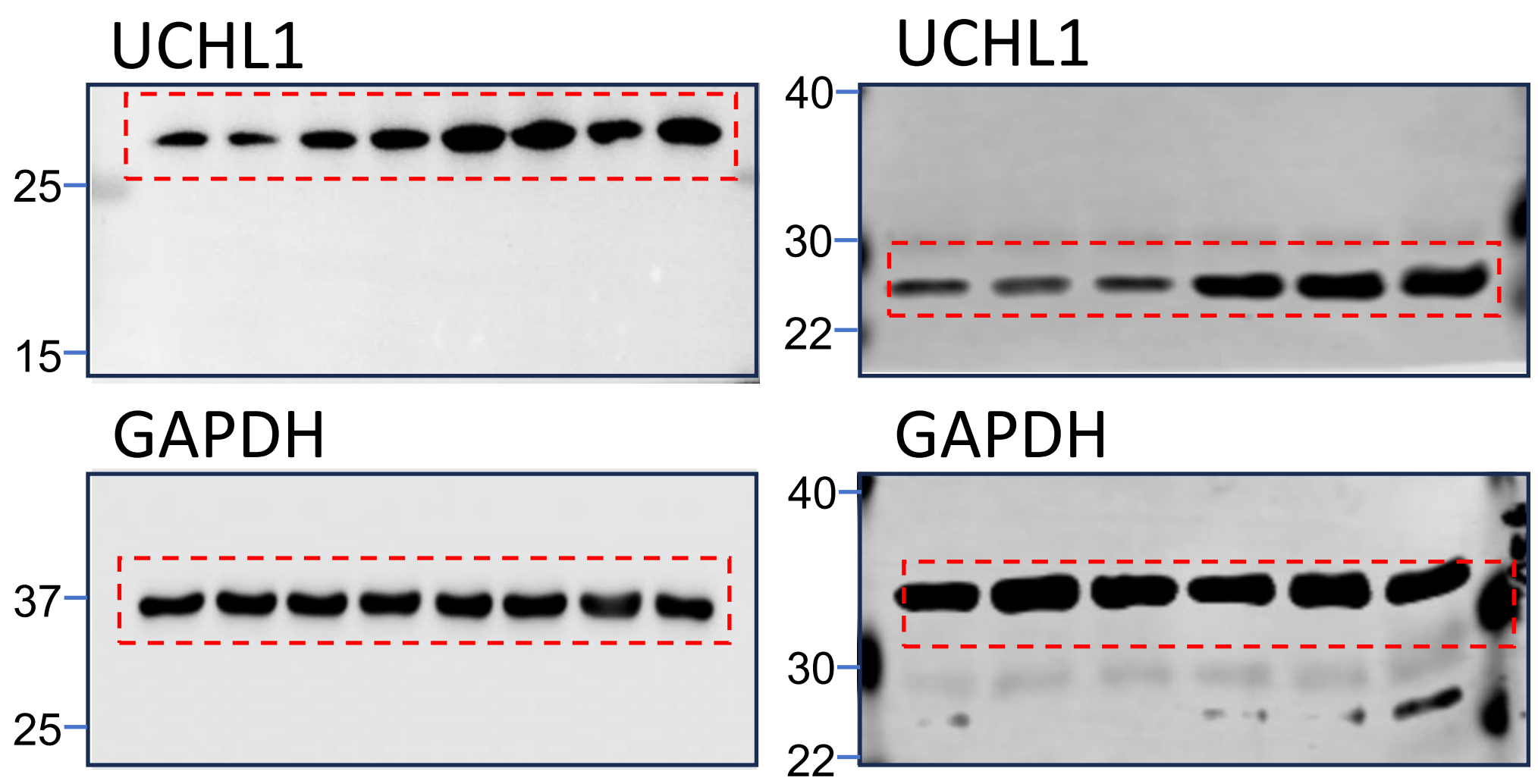

Full unedited gel for Figure 2B

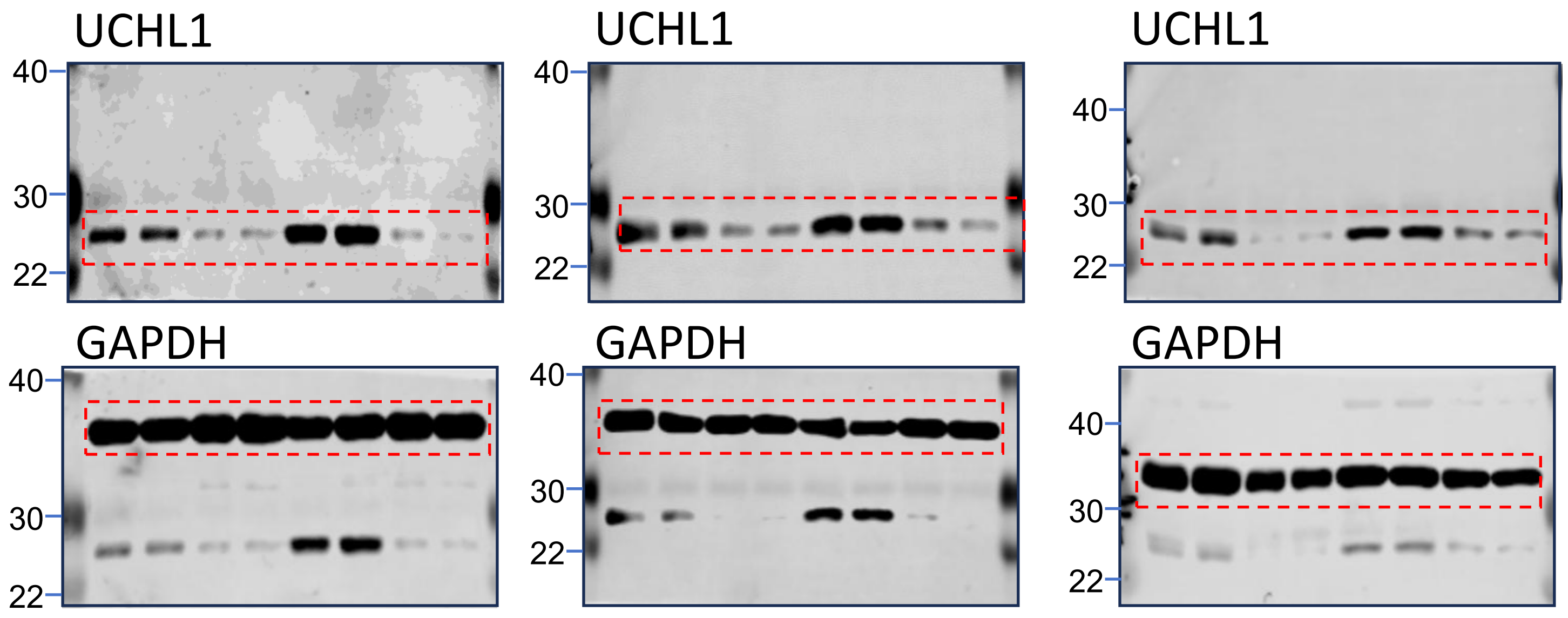

Full unedited gel for Figure 2J

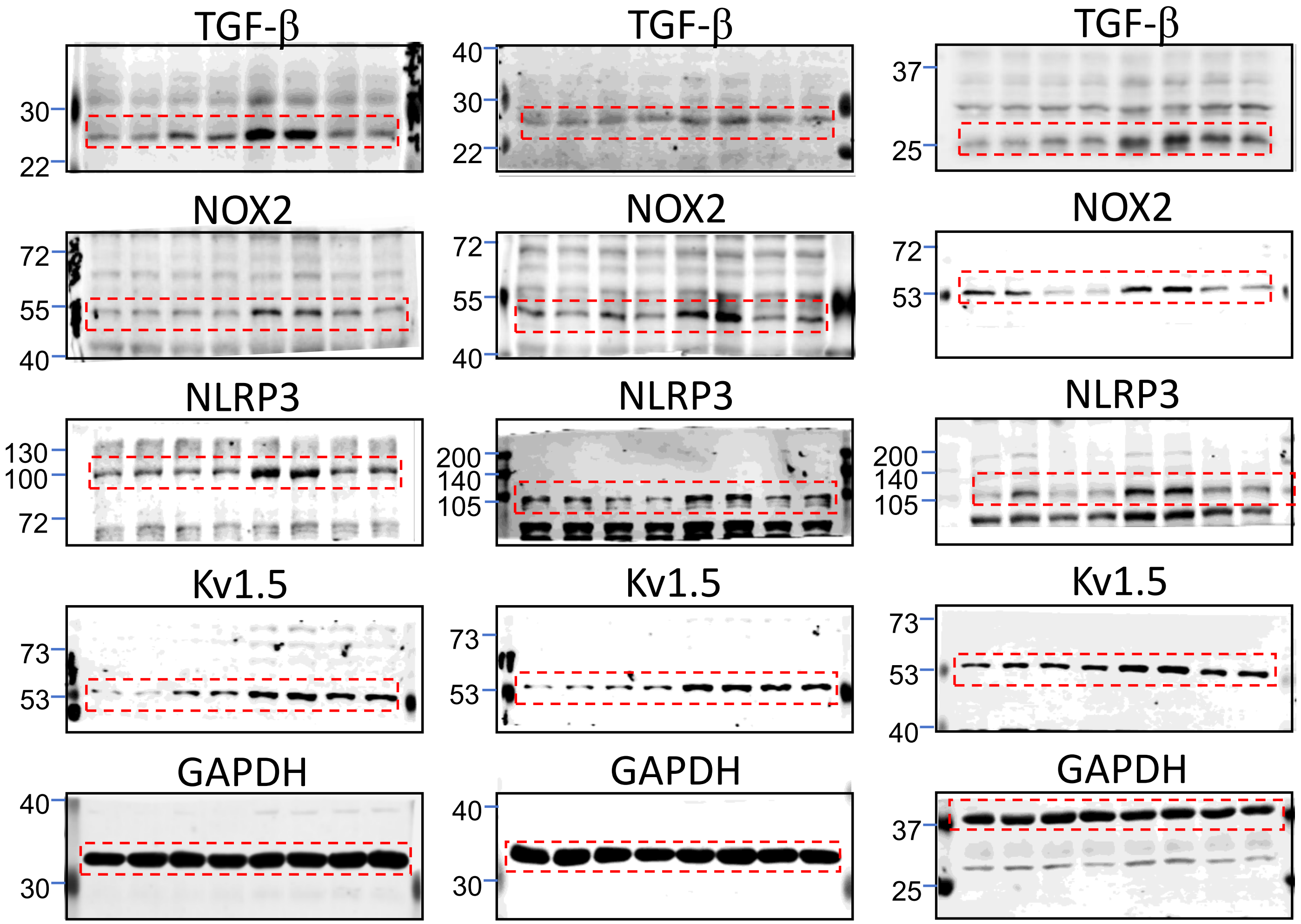

Full unedited gel for Figure 3B

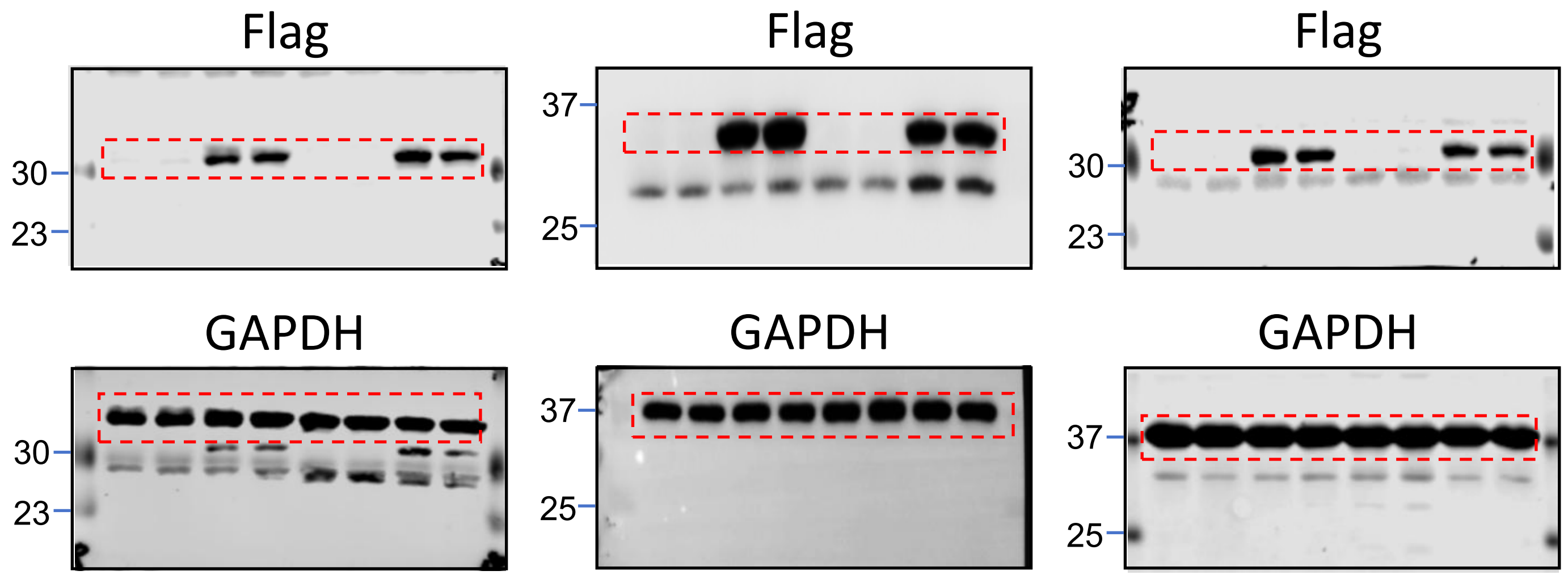

Full unedited gel for Figure 3J

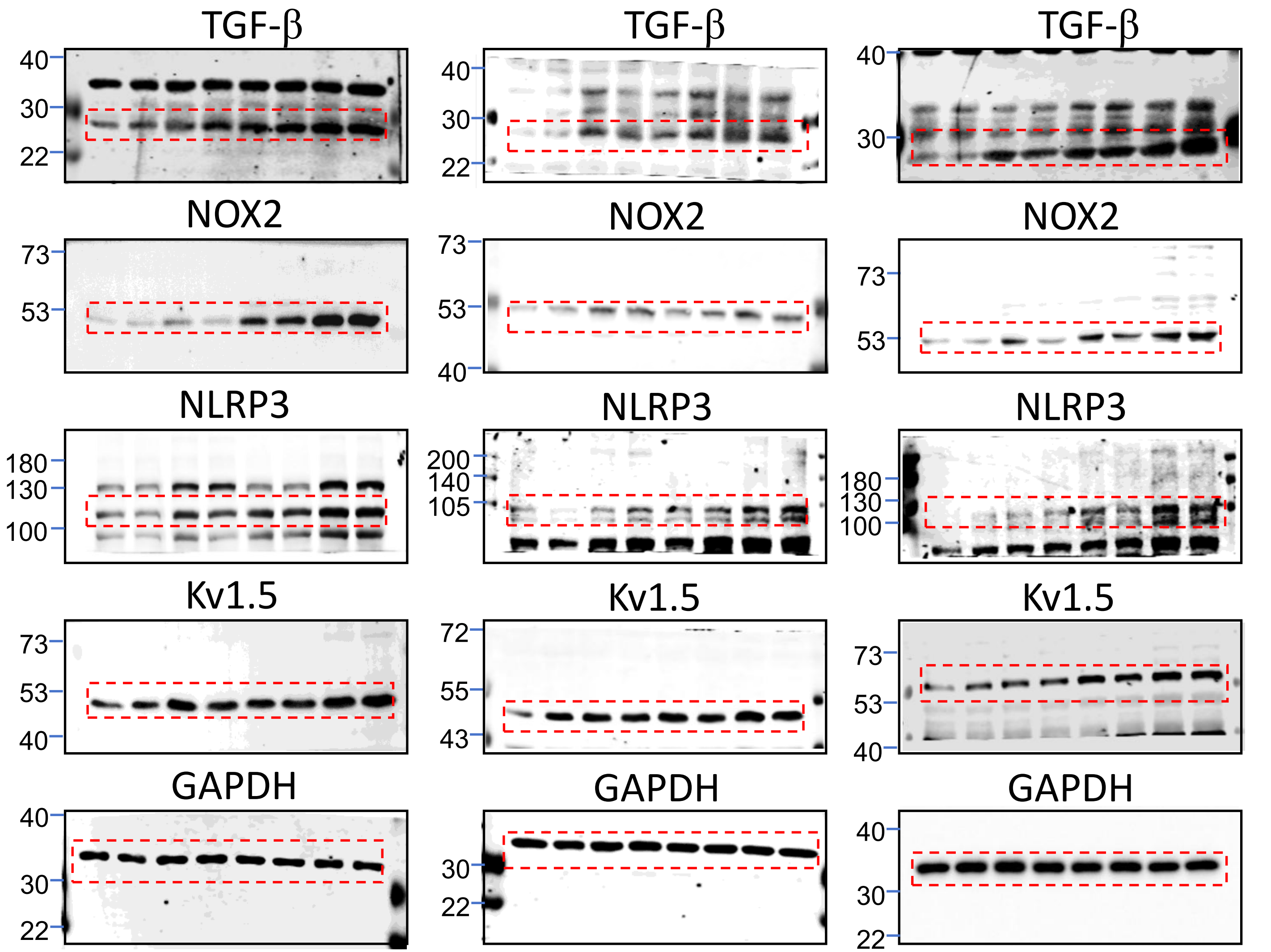

Full unedited gel for Figure 4D

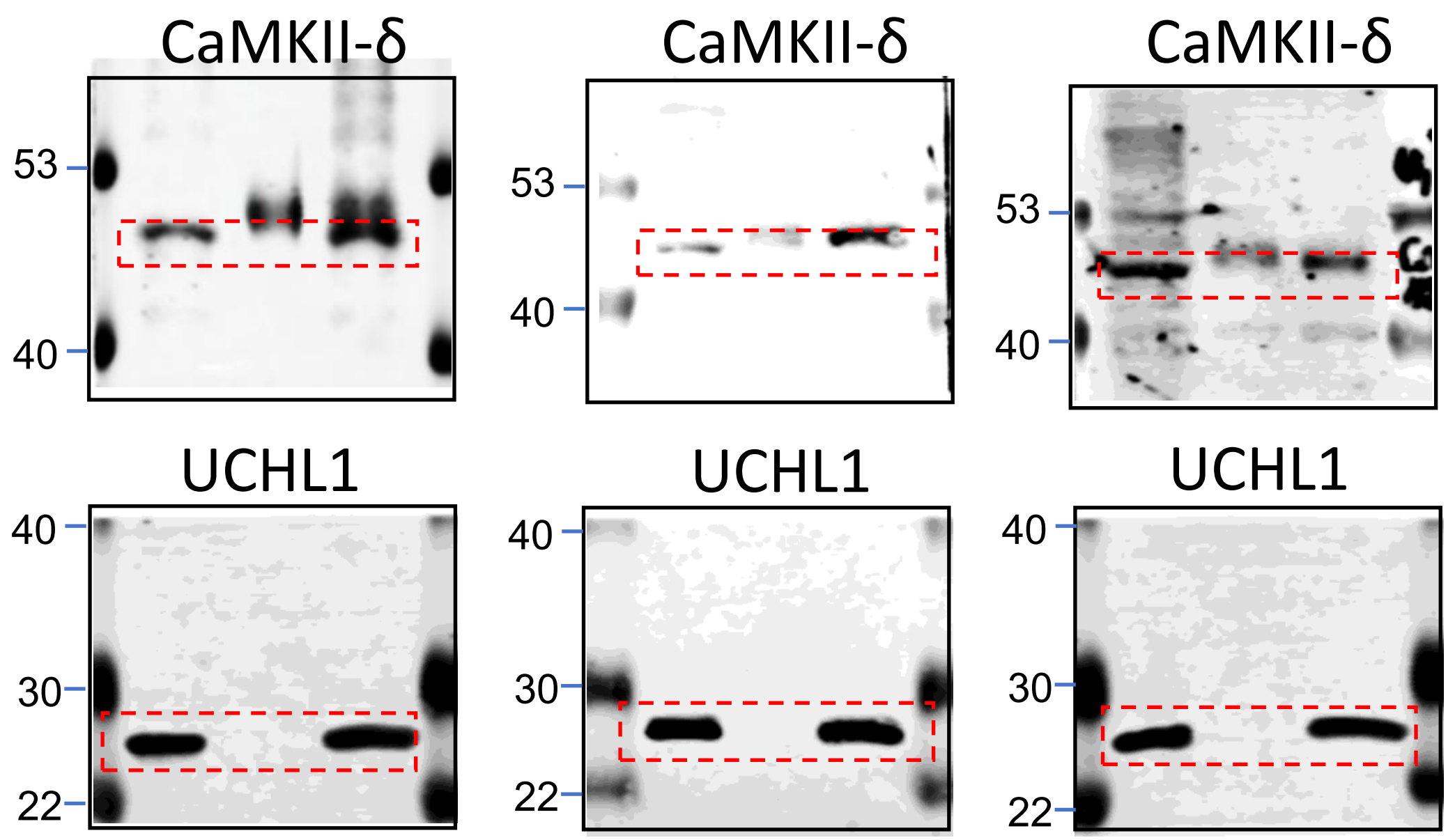

Full unedited gel for Figure 4E

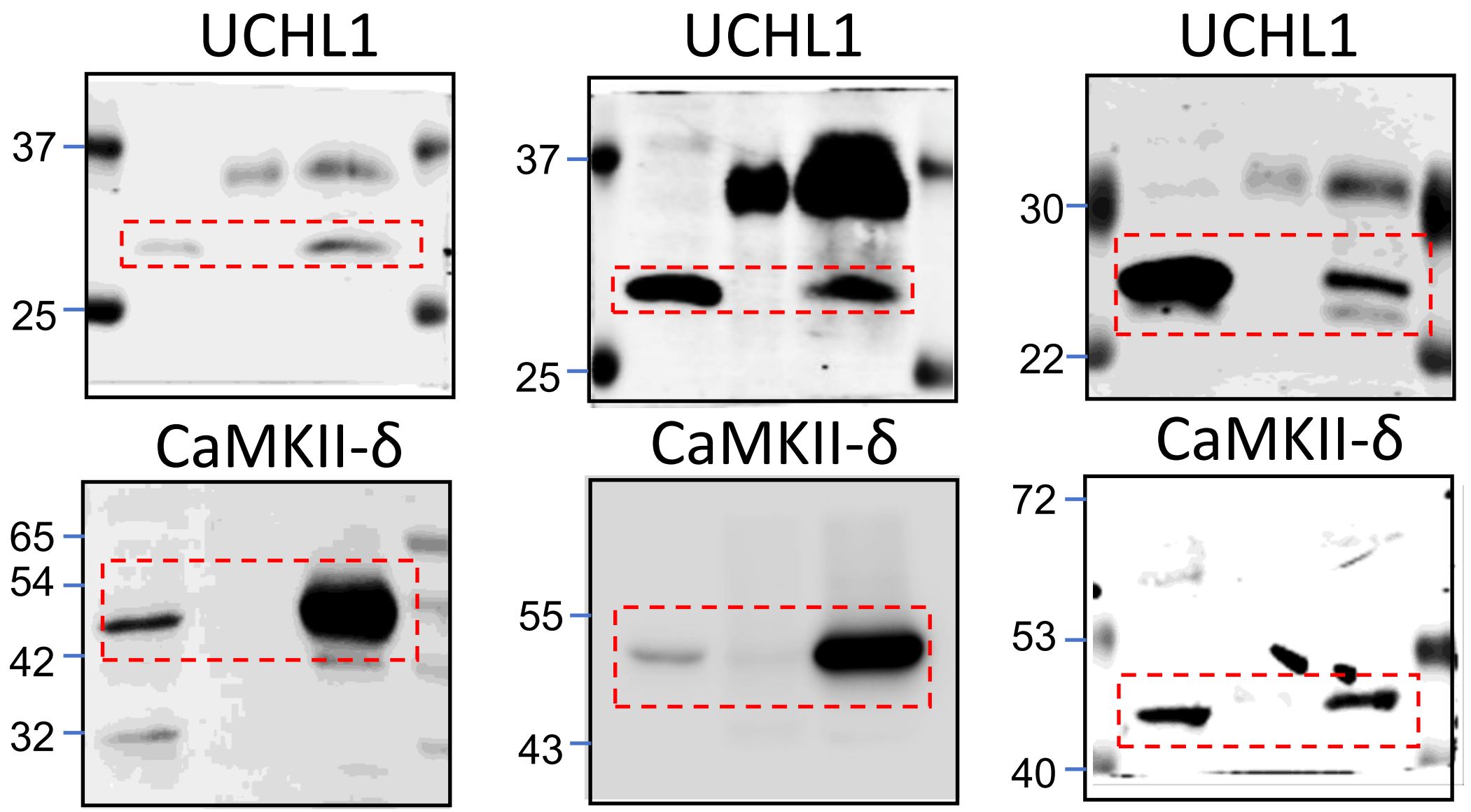

Full unedited gel for Figure 4F

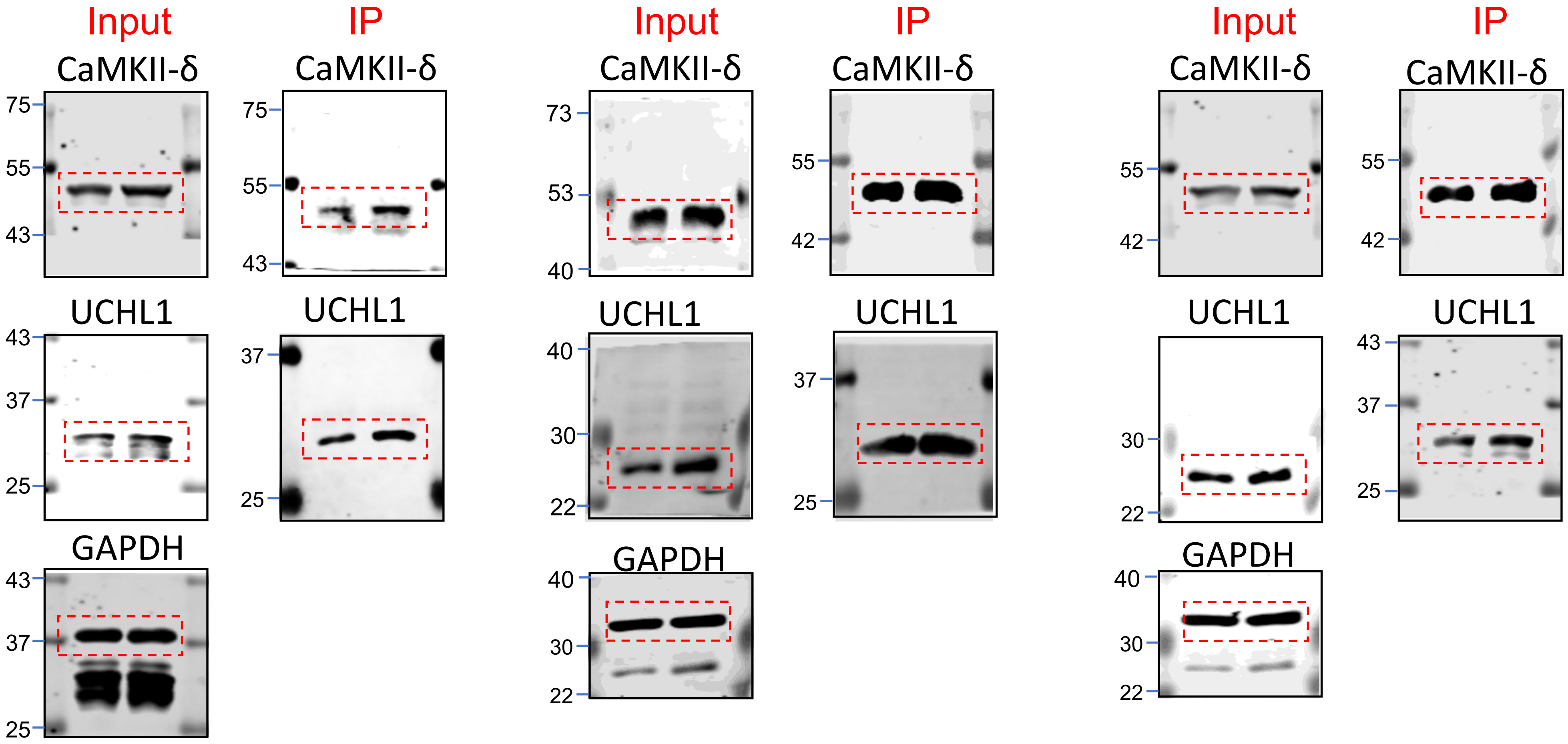

Full unedited gel for Figure 4G

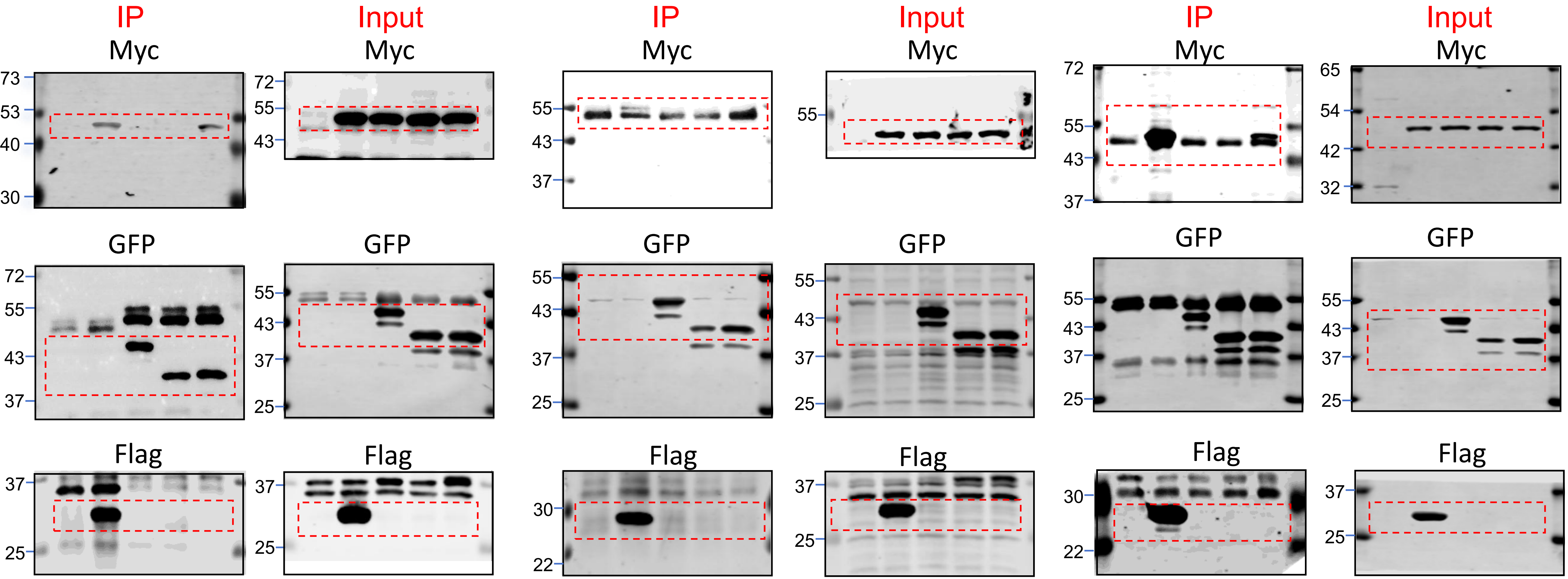

Full unedited gel for Figure 4I

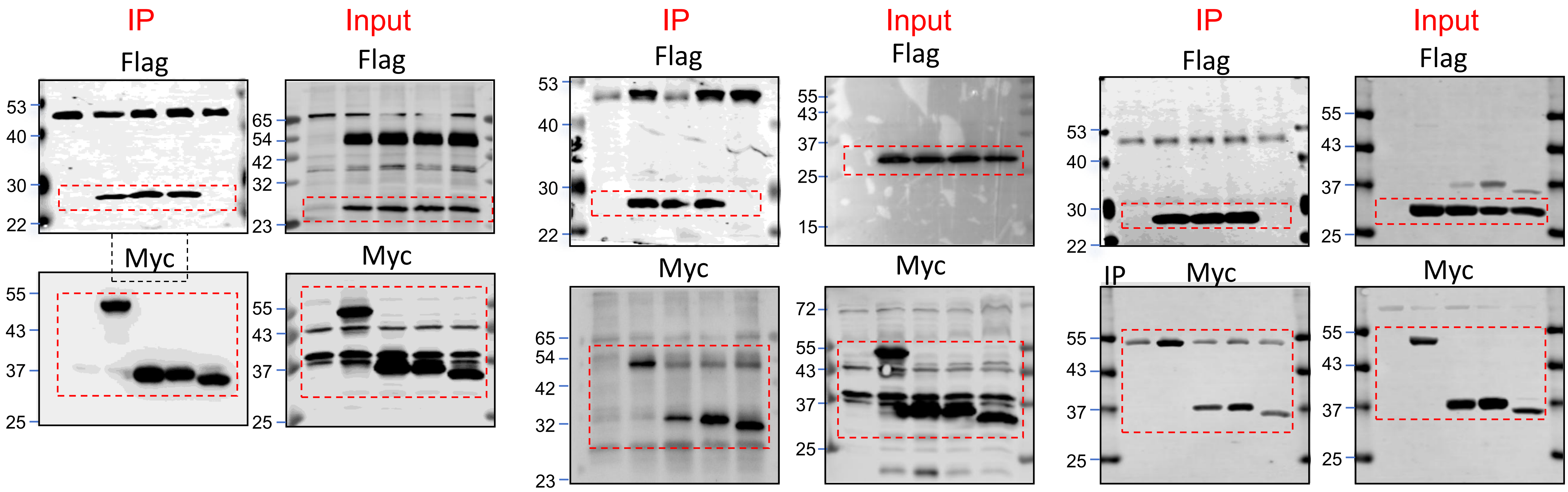

Full unedited gel for Figure 5D

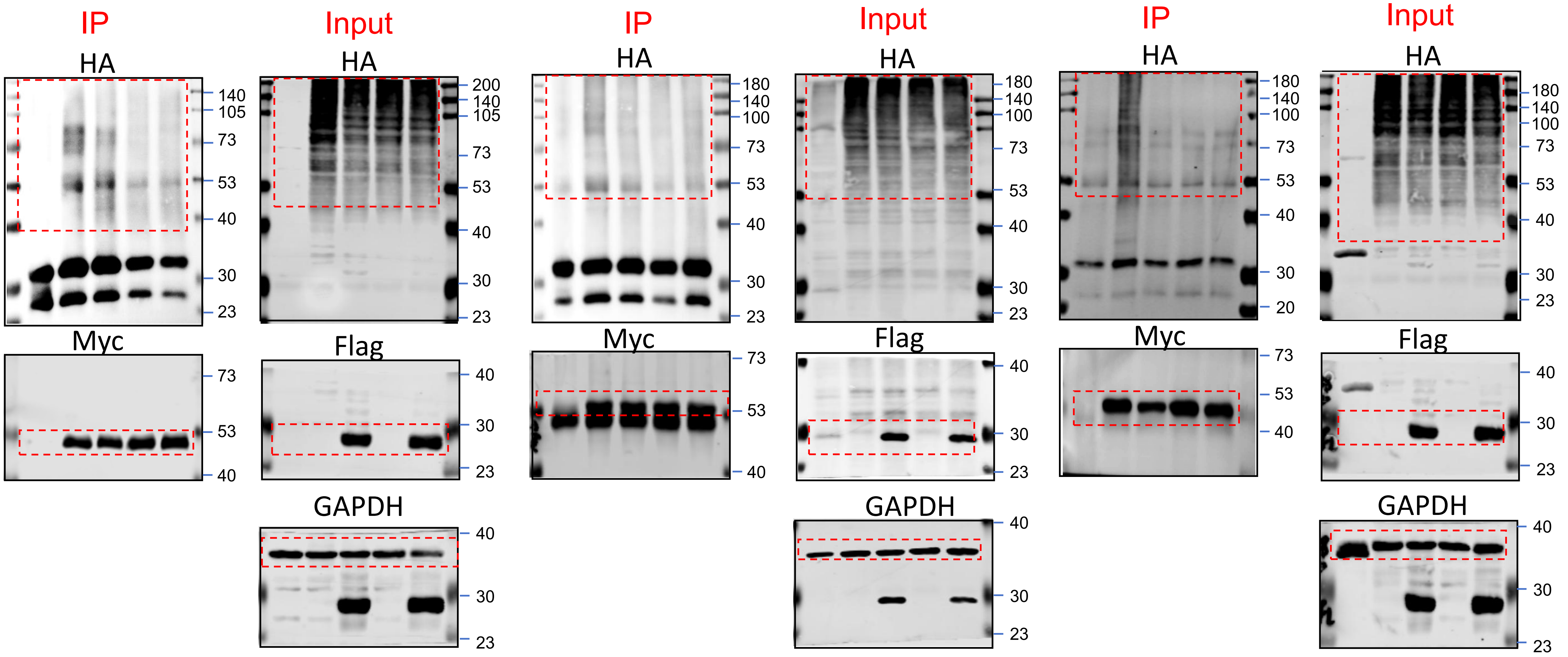

Full unedited gel for Figure 5E

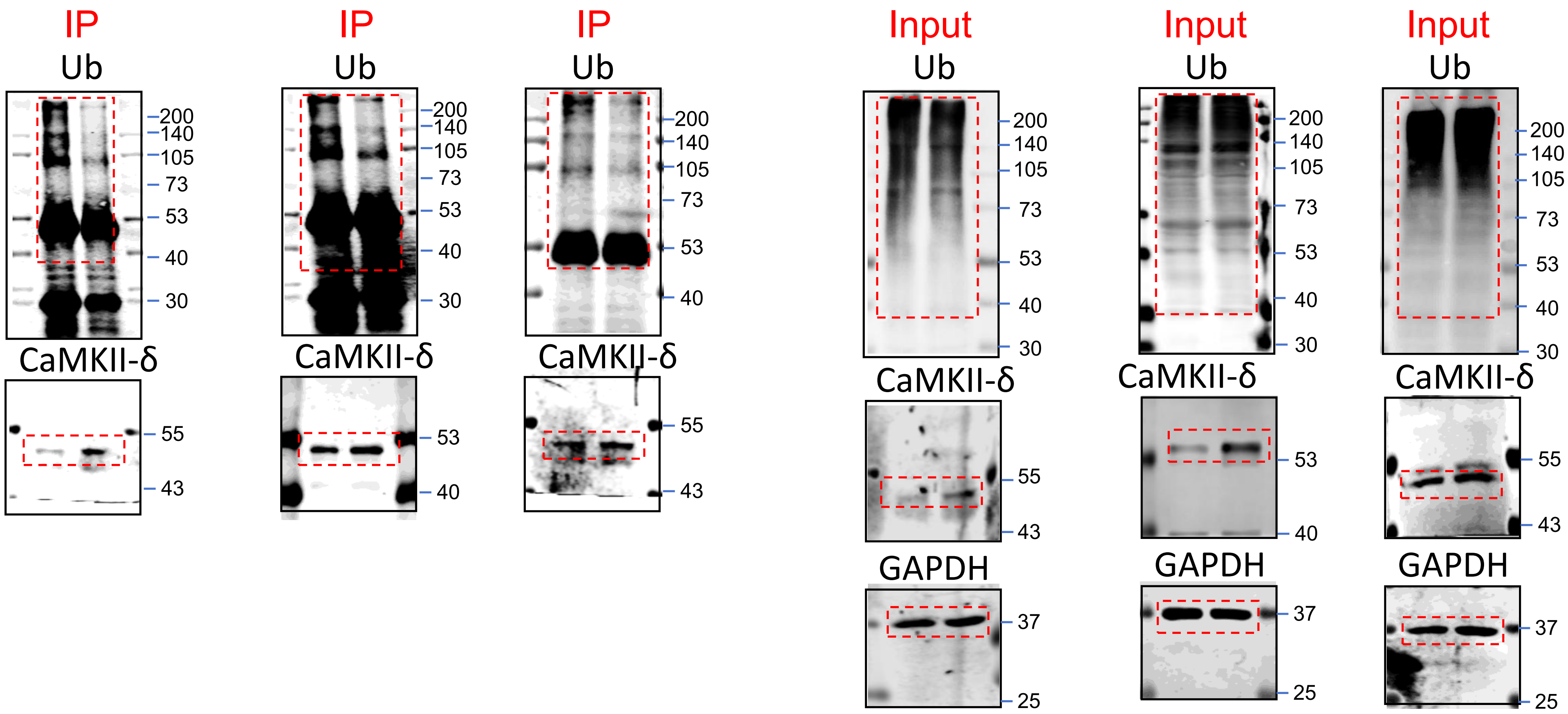

Full unedited gel for Figure 5F

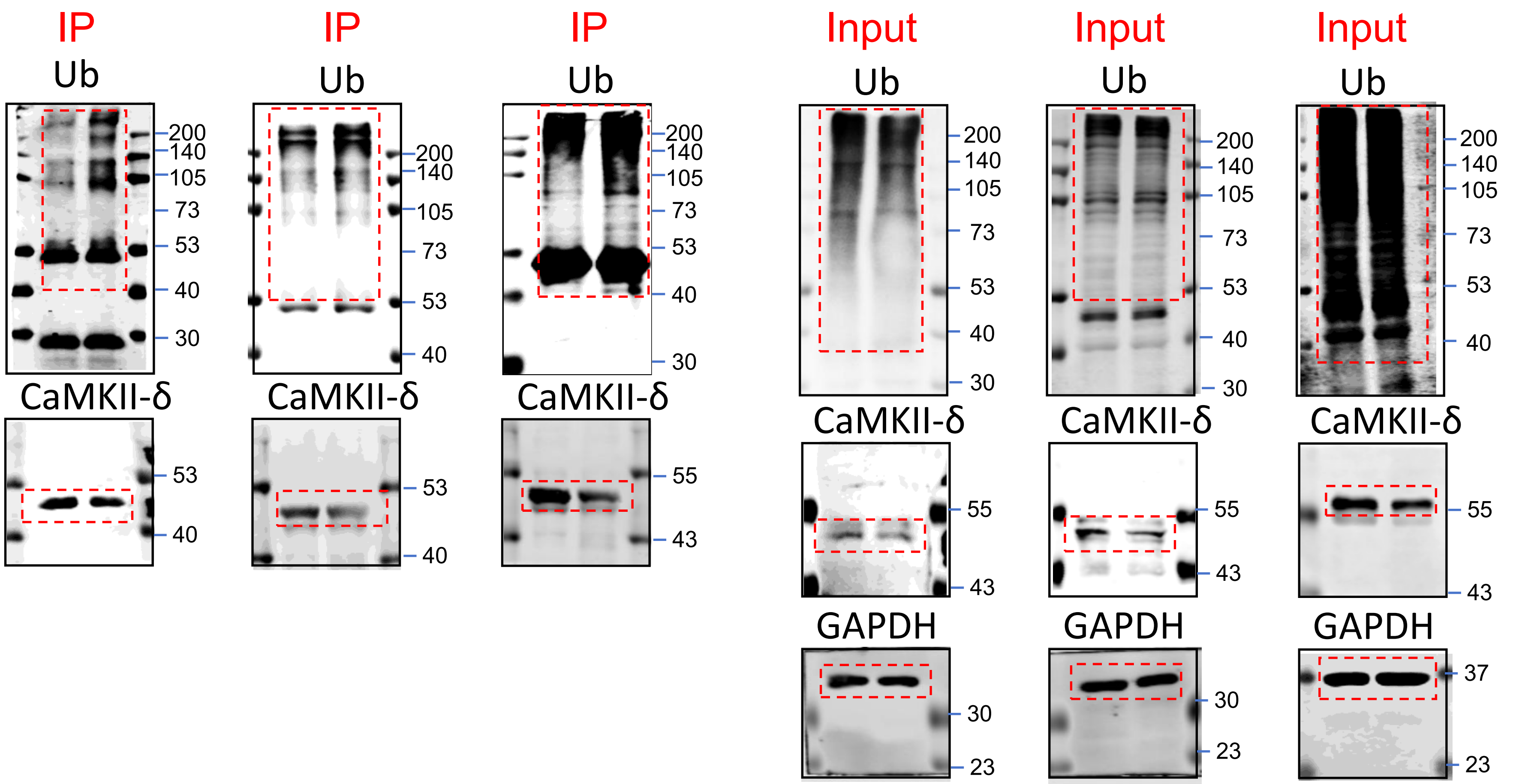

Full unedited gel for Figure 5G

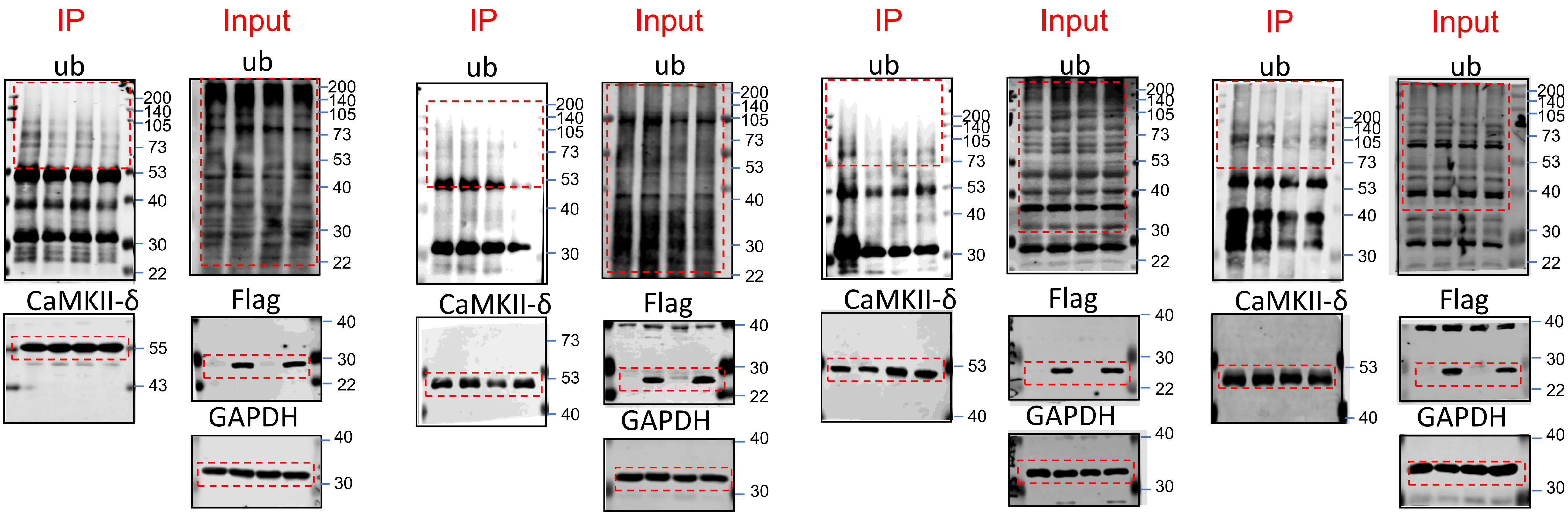

Full unedited gel for Figure 5H

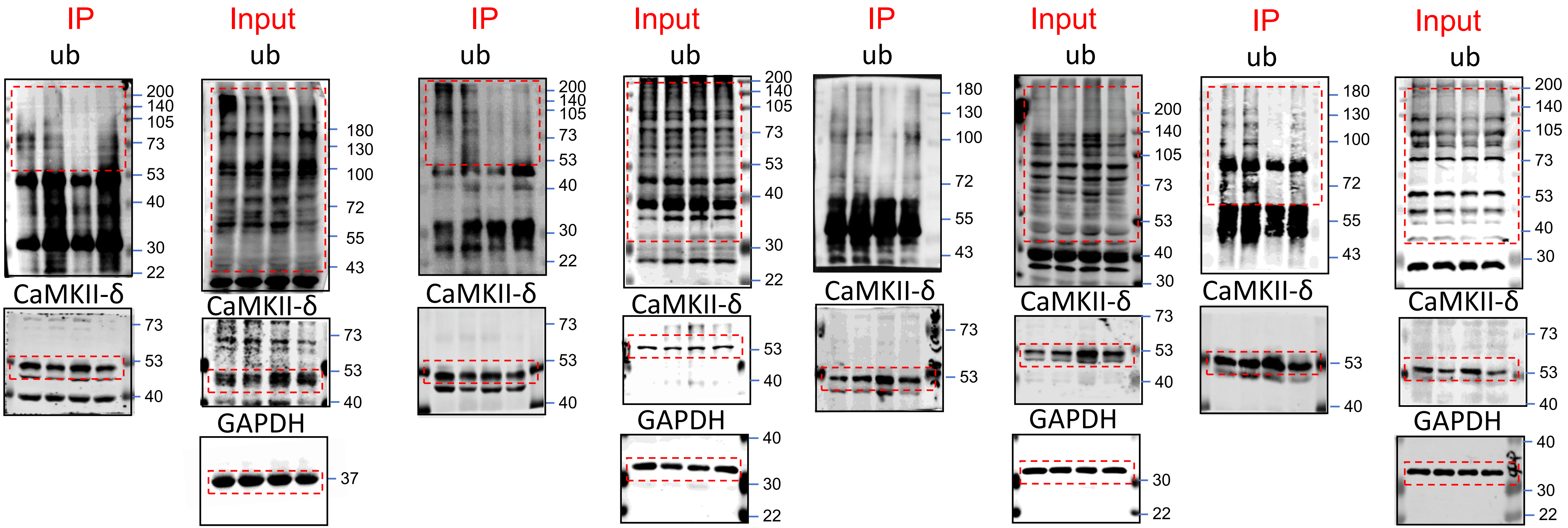

Full unedited gel for Figure 5J

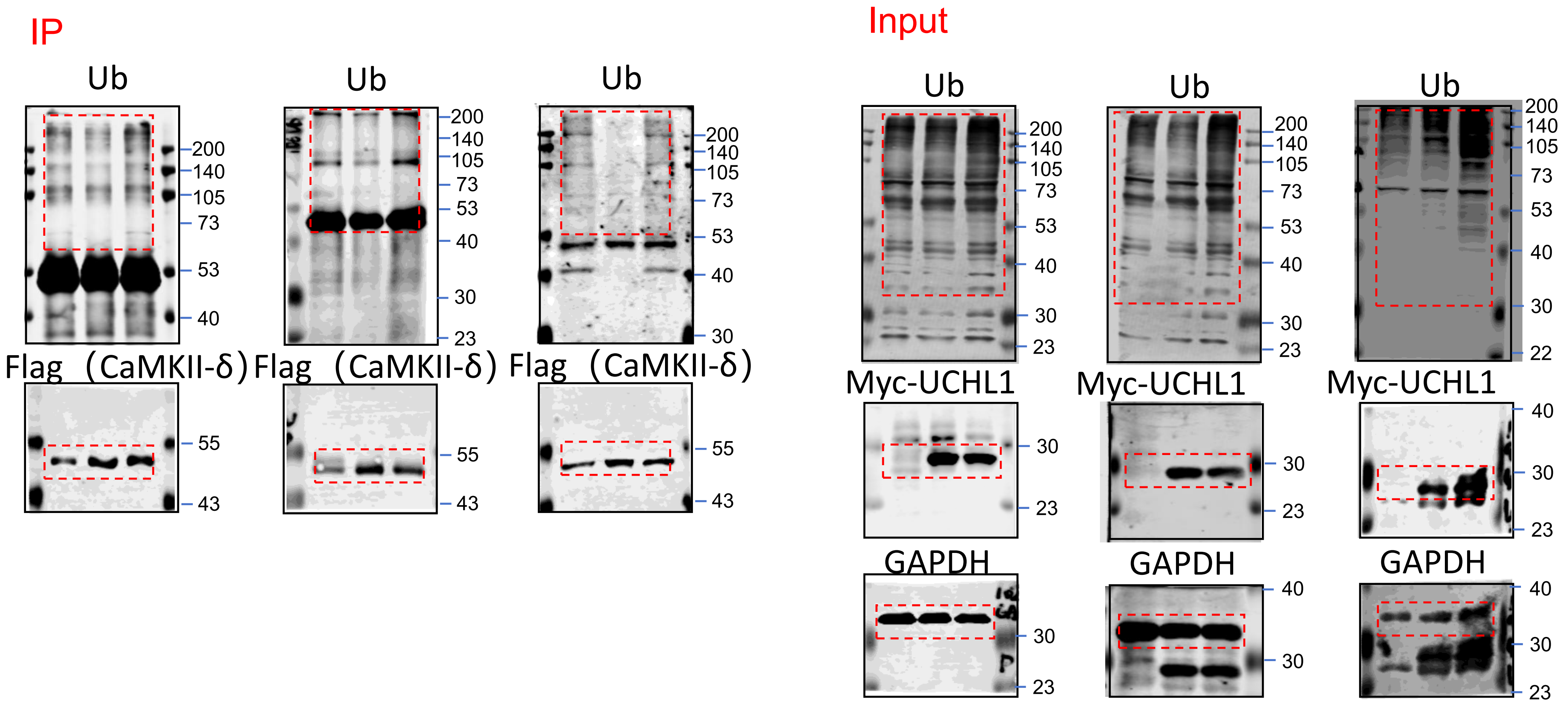

Full unedited gel for Figure 5K

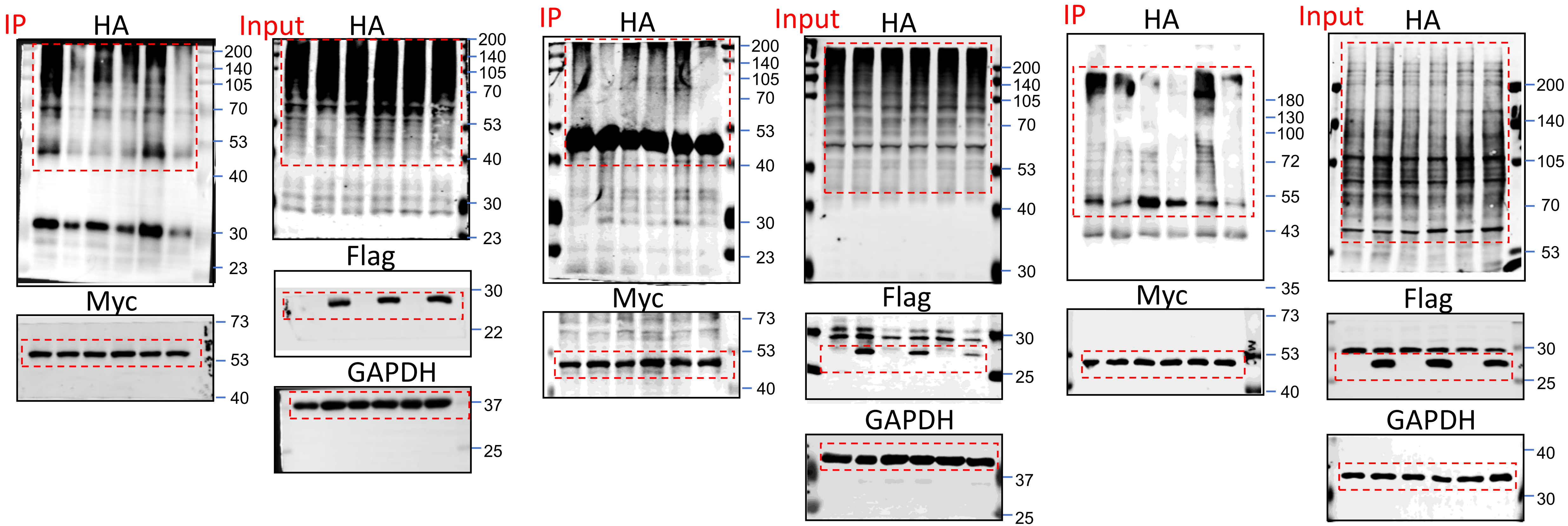

Full unedited gel for Figure 5L

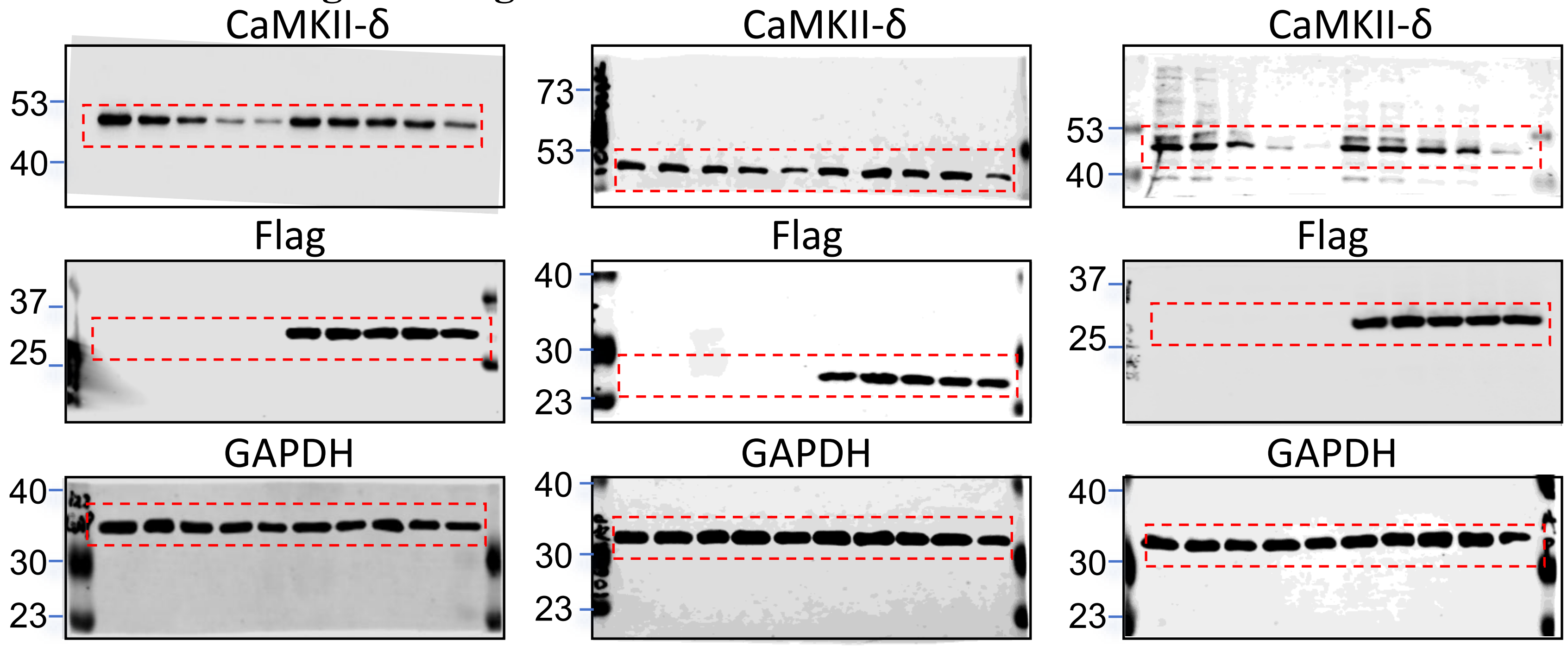

Full unedited gel for Figure 5M

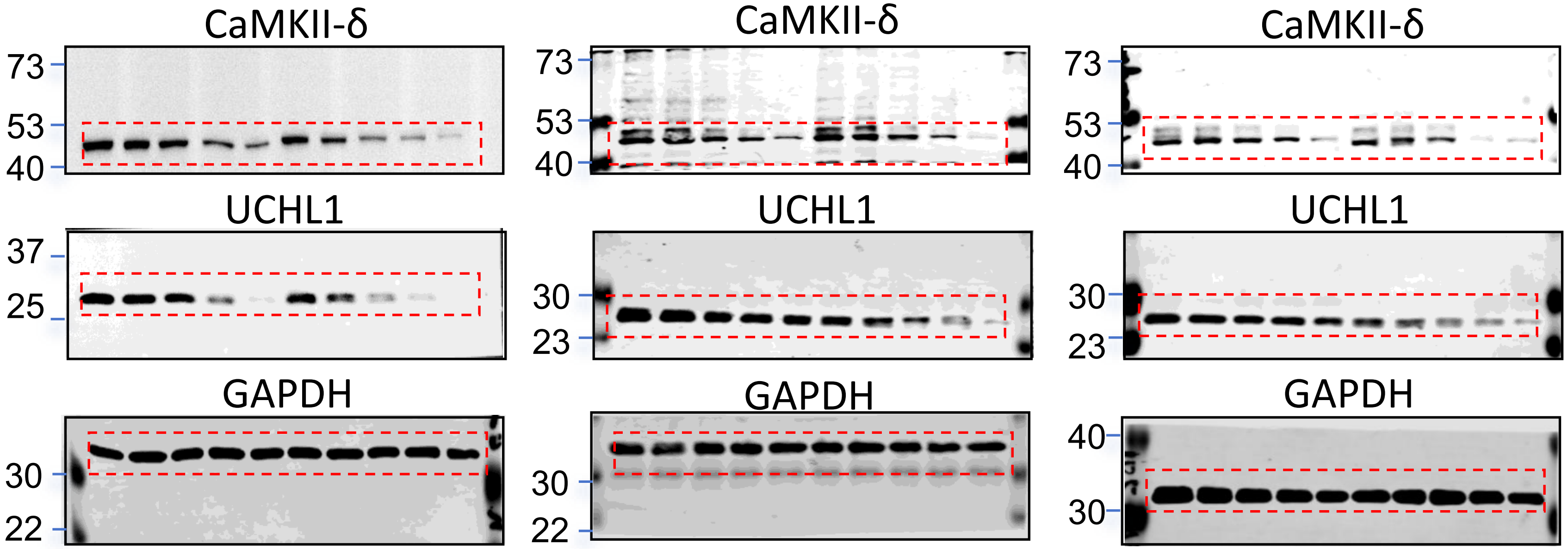

Full unedited gel for Figure 6A

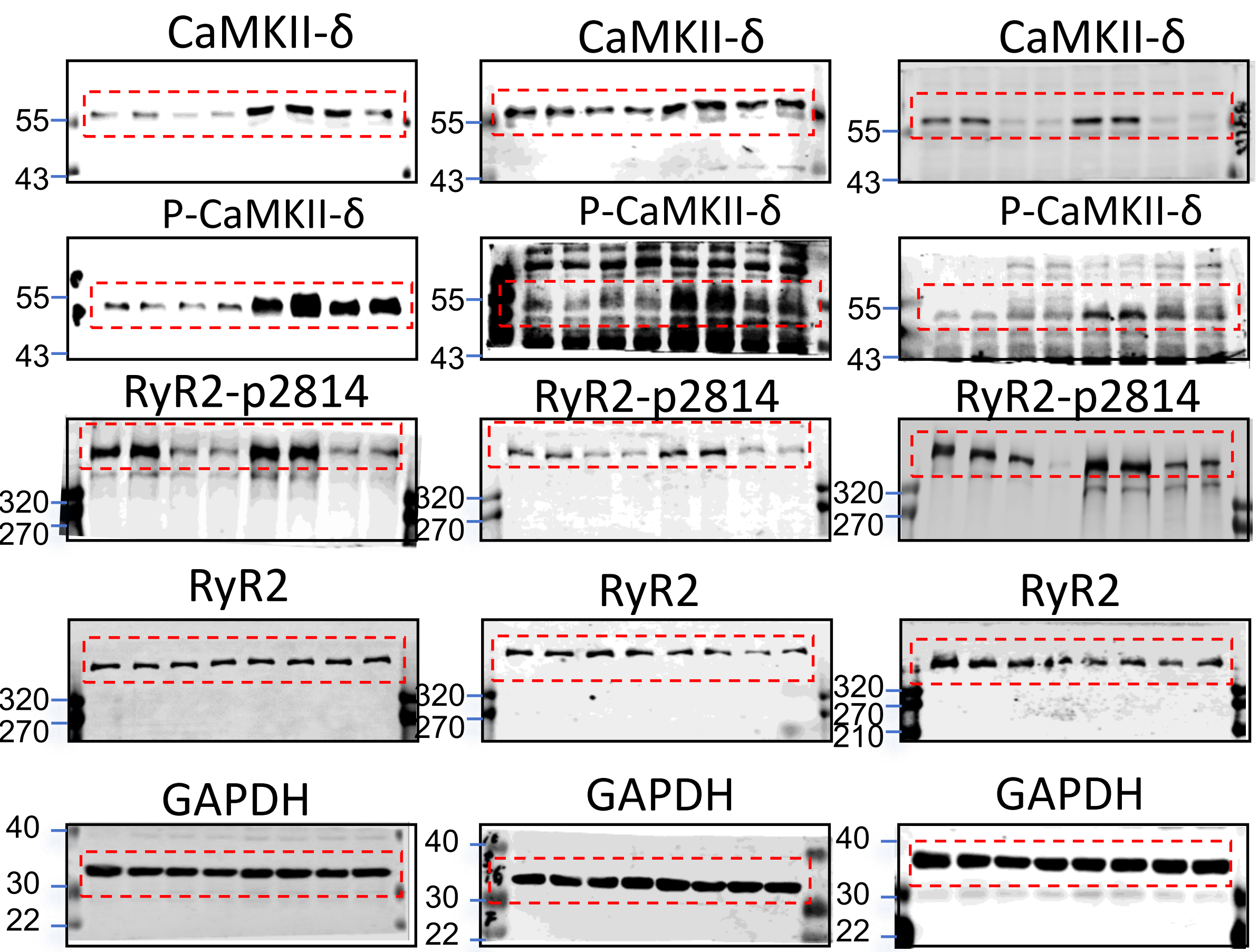

Full unedited gel for Figure 6B

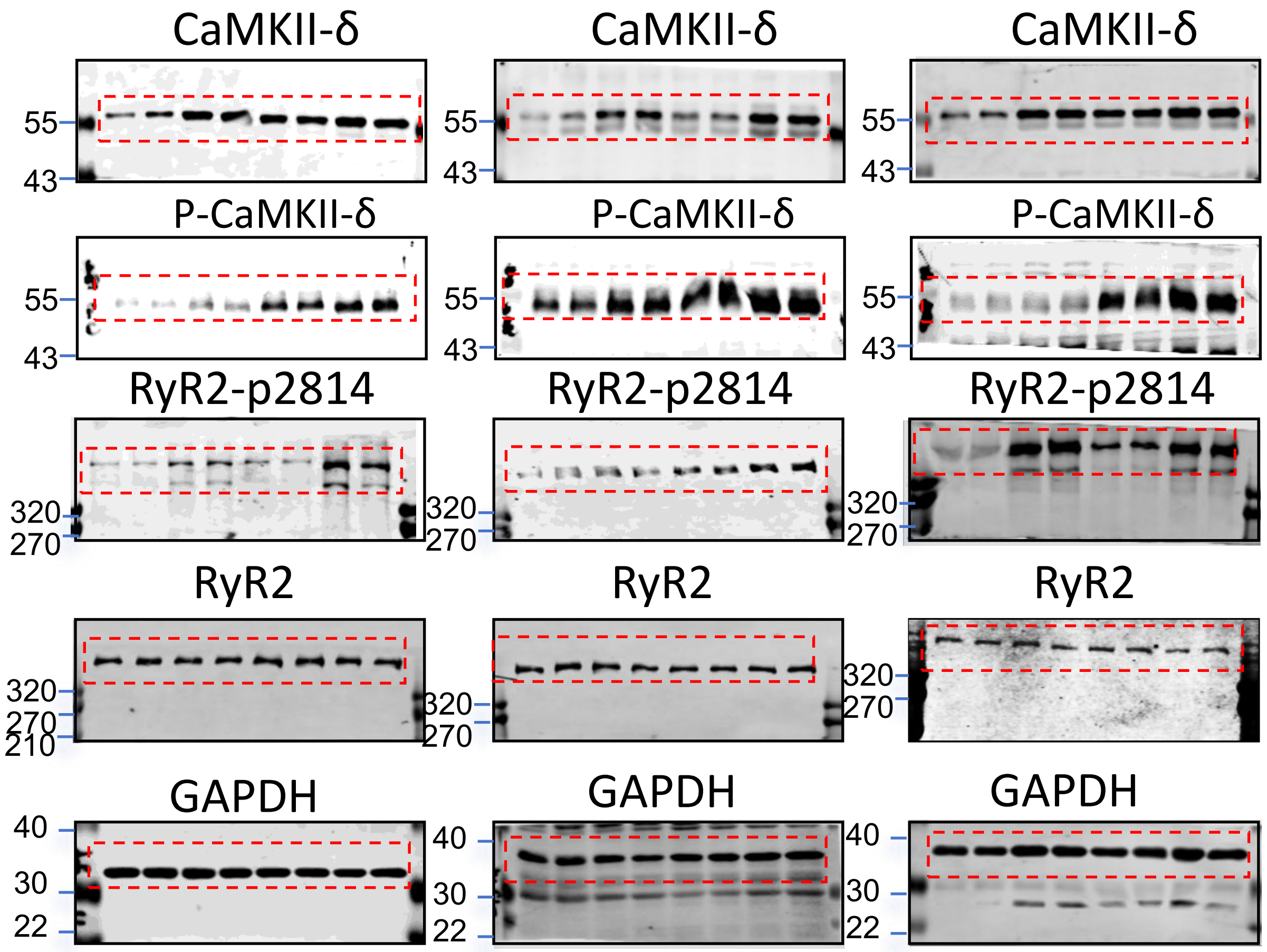

Full unedited gel for Figure 6C

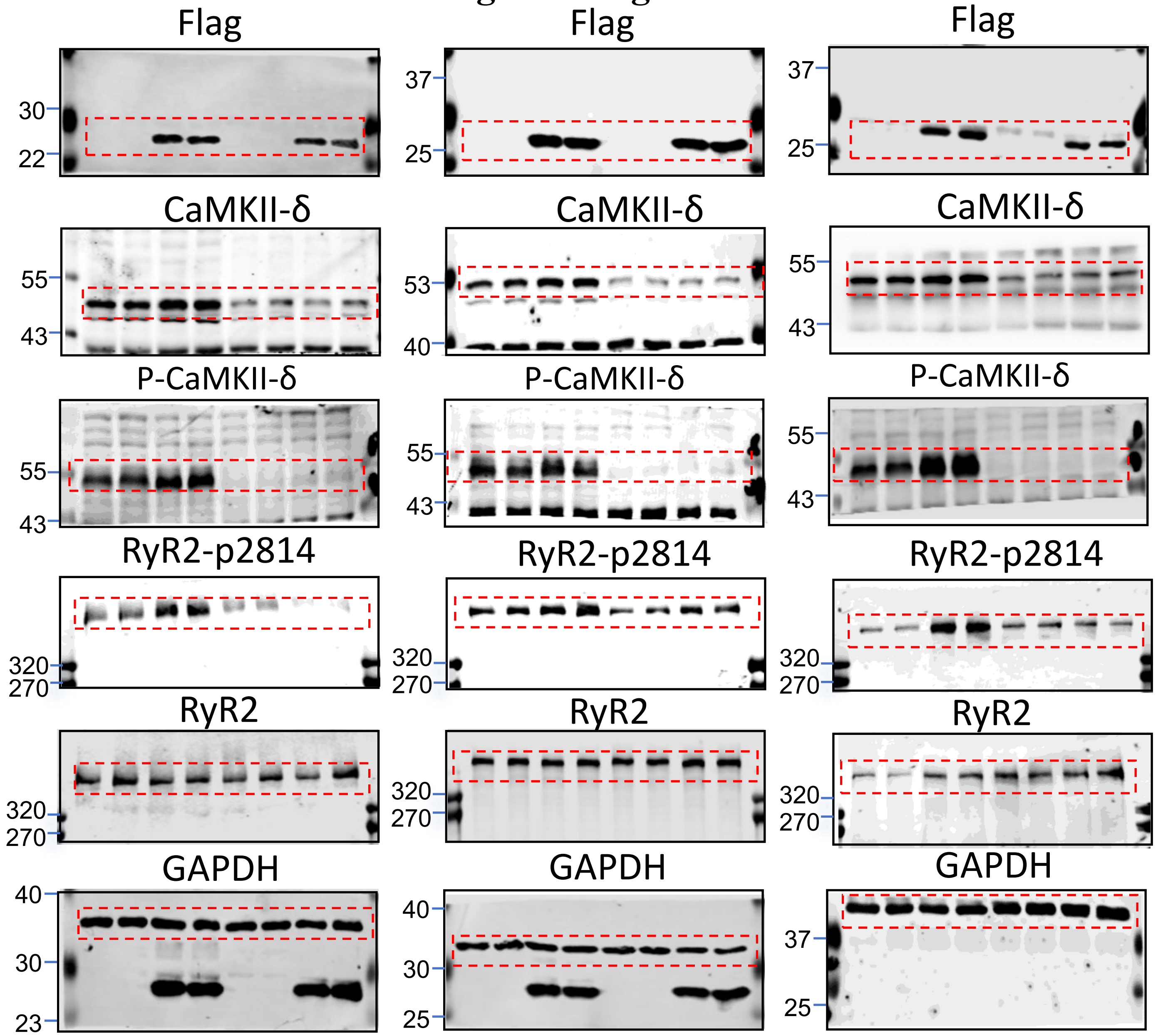

Full unedited gel for Figure 7I

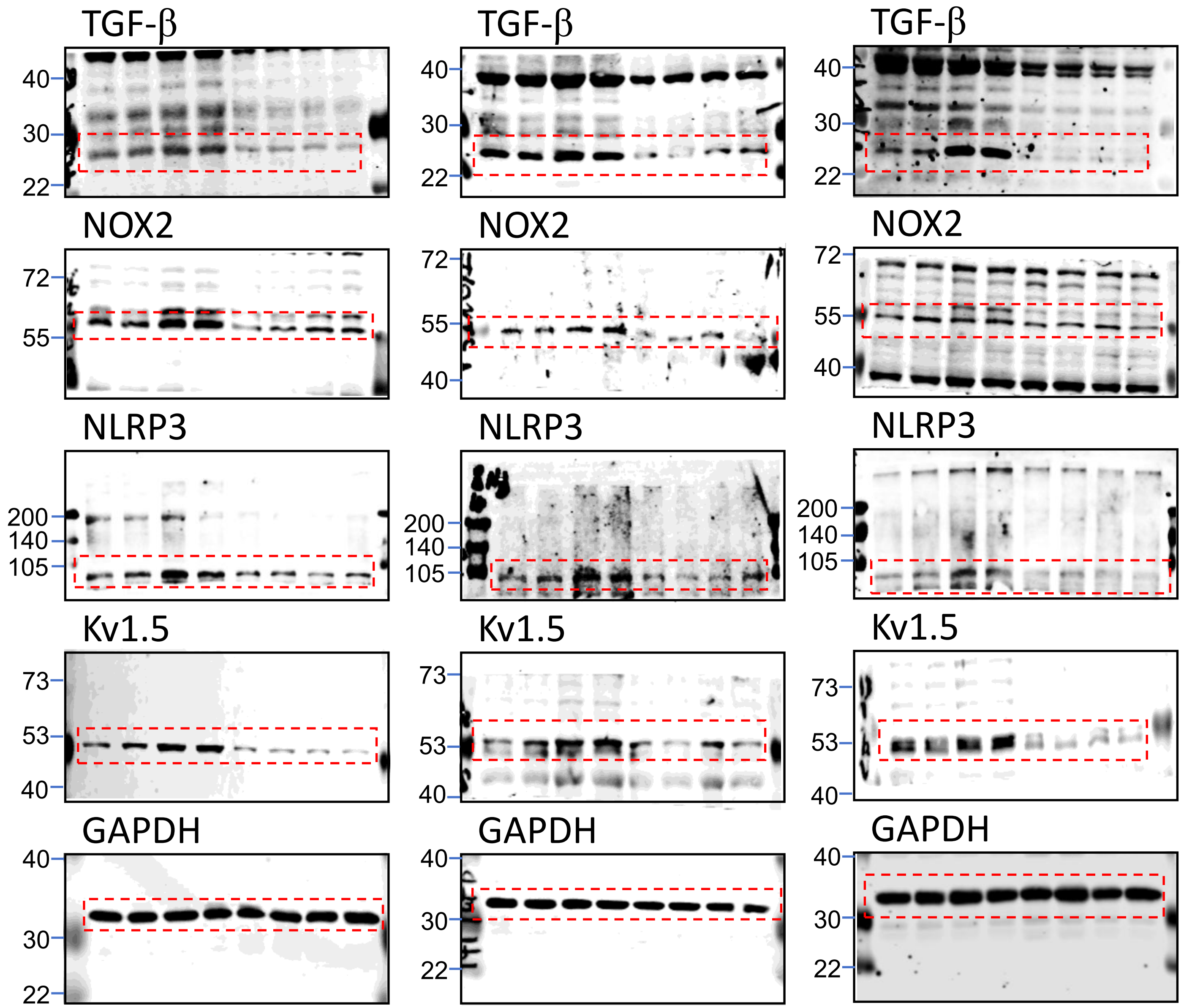

Full unedited gel for Figure 8B

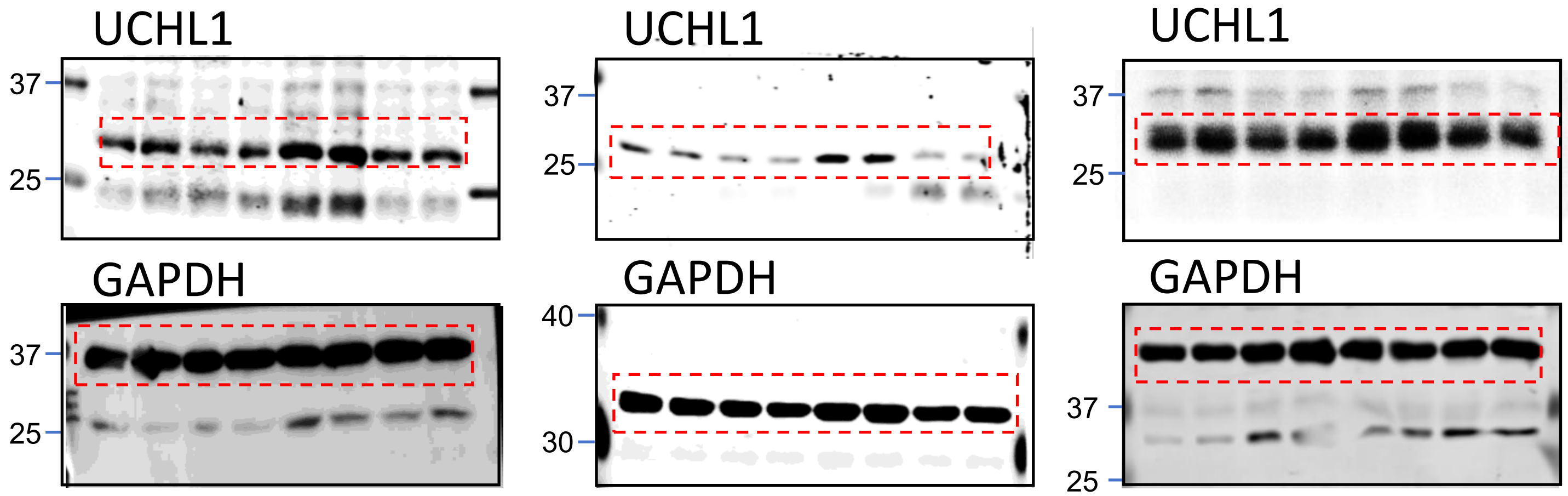

Full unedited gel for Figure 8J

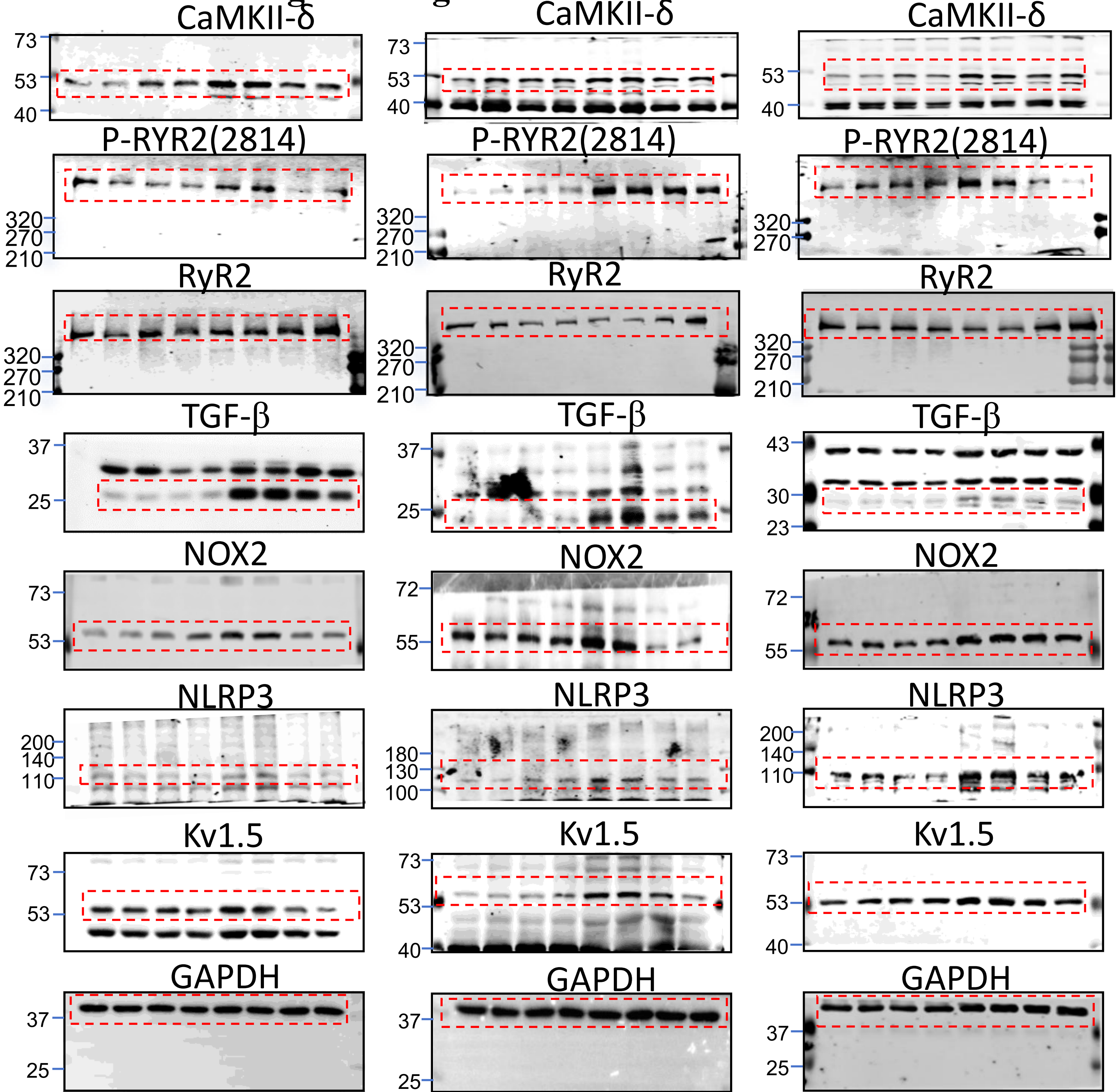

Full unedited gel for Figure S1B

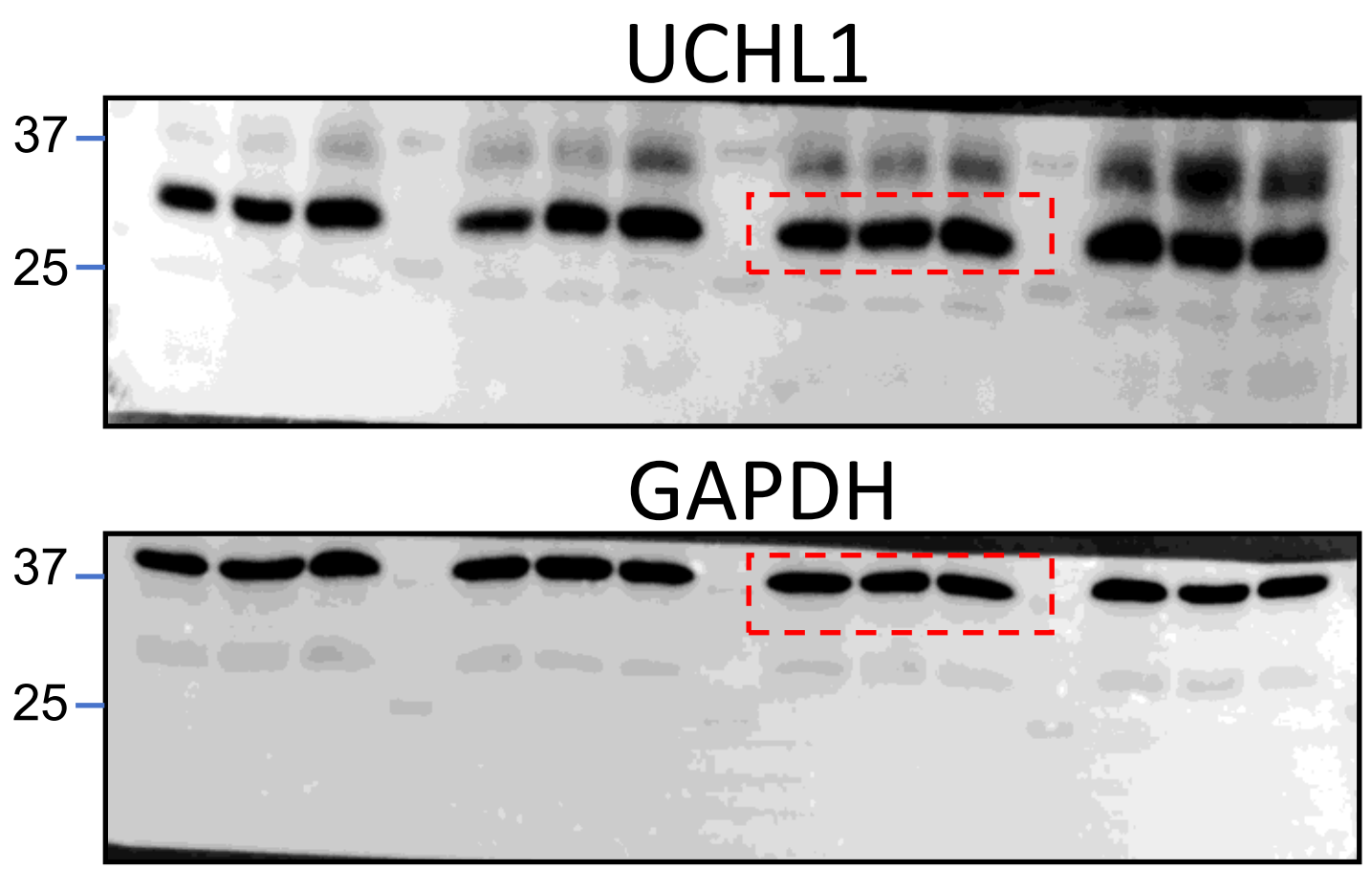

Full unedited gel for Figure S2B

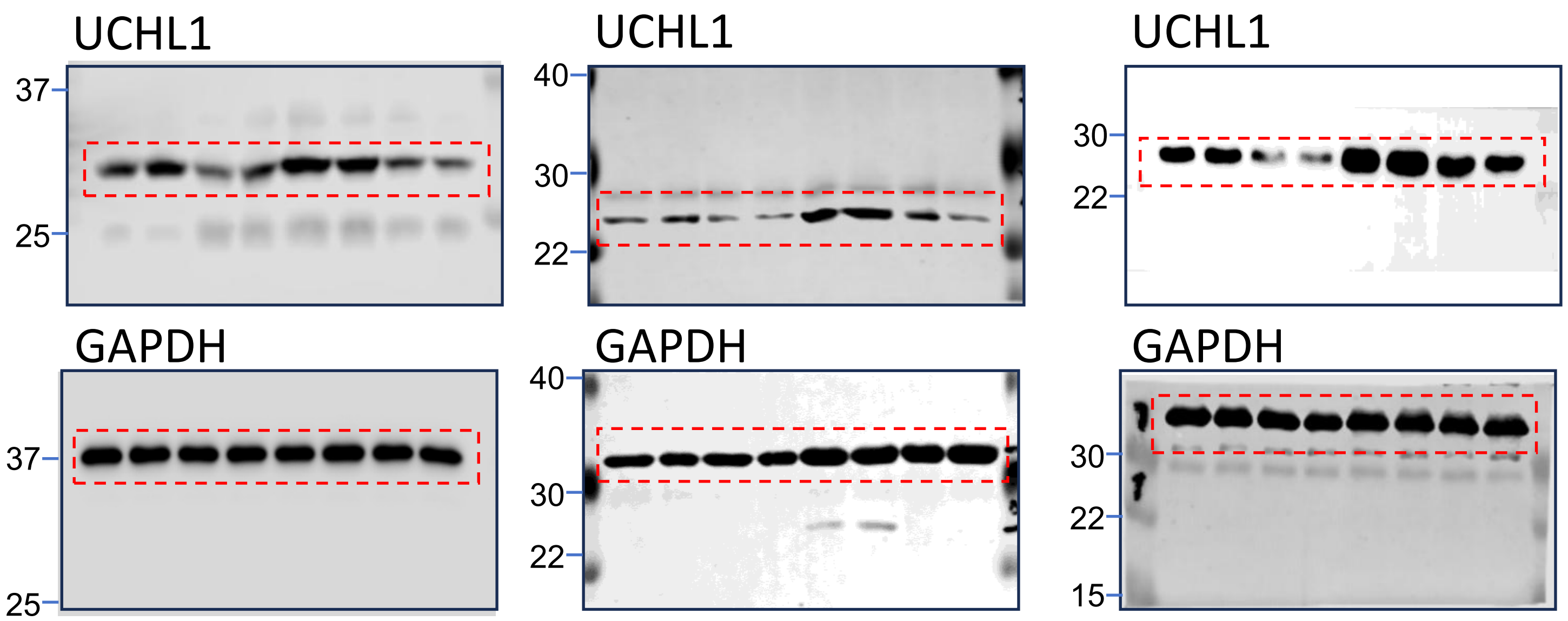

Full unedited gel for Figure S3D

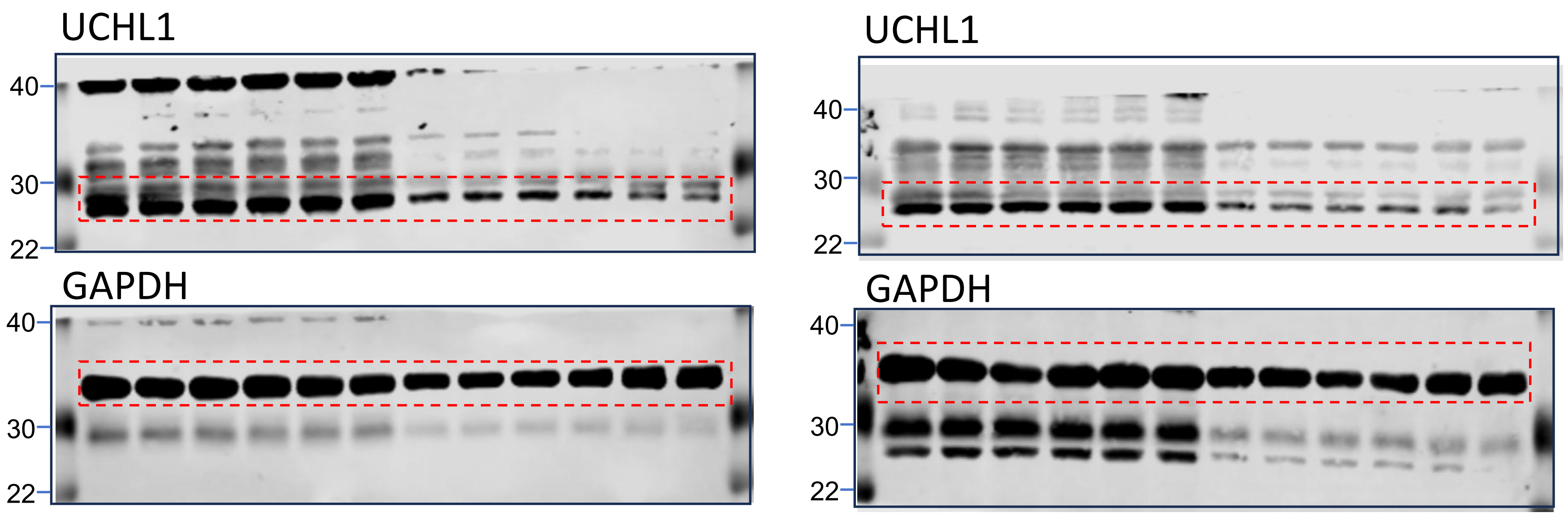

Full unedited gel for Figure S6A

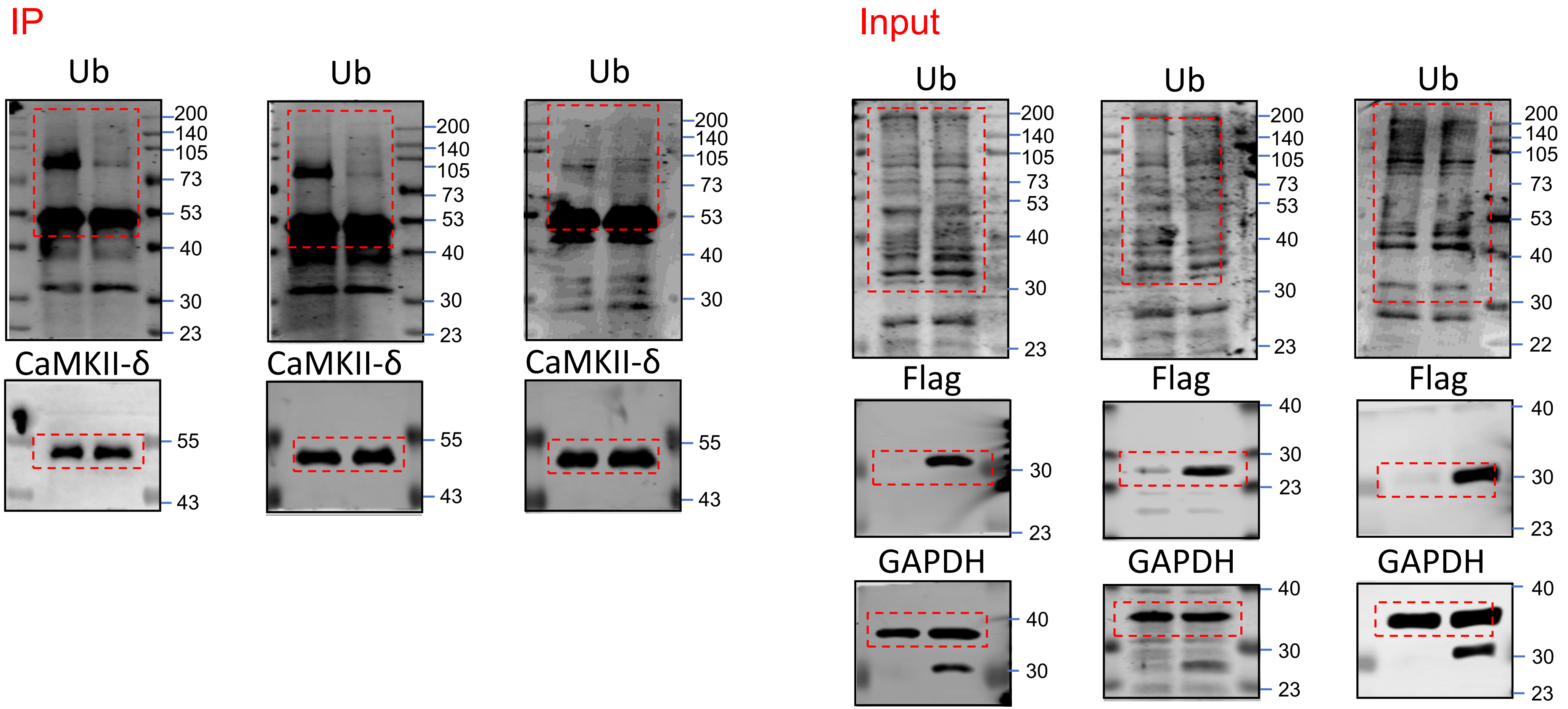

Full unedited gel for Figure S6B

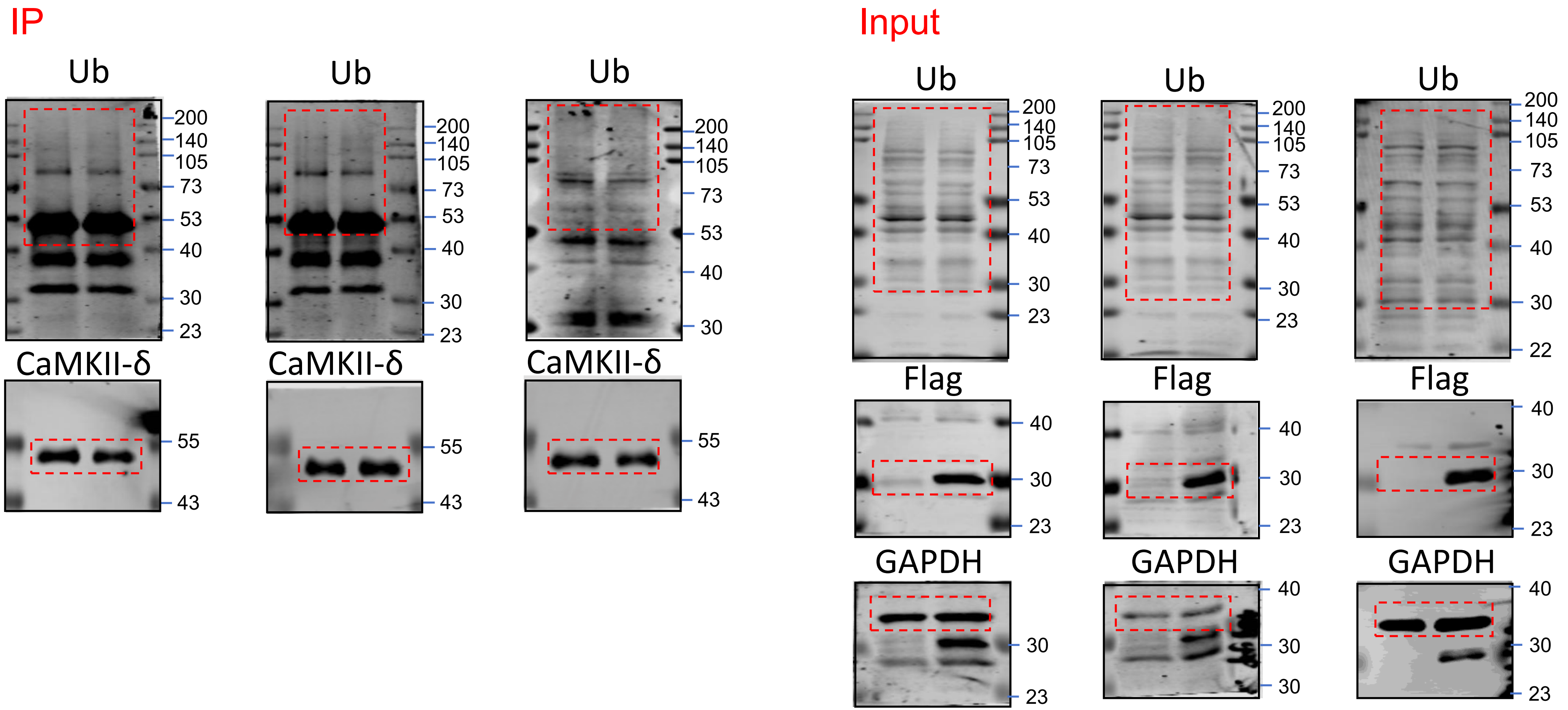

Full unedited gel for Figure S7A

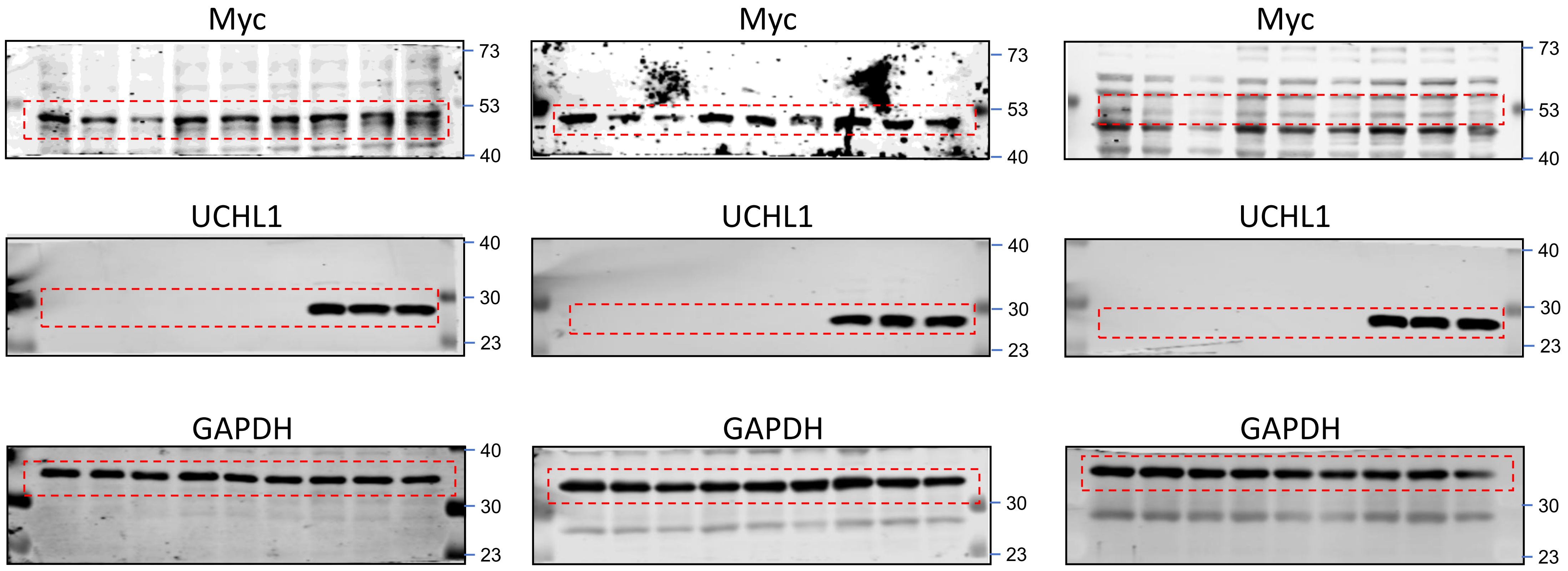

Full unedited gel for Figure S9B

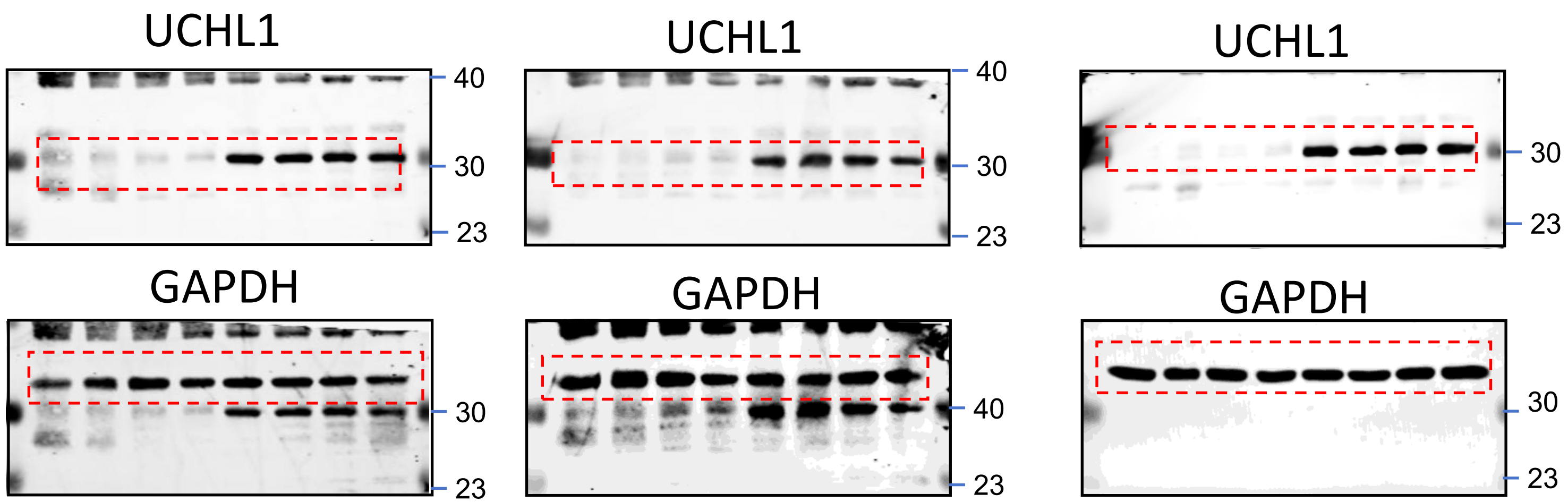

Full unedited gel for Figure S10B

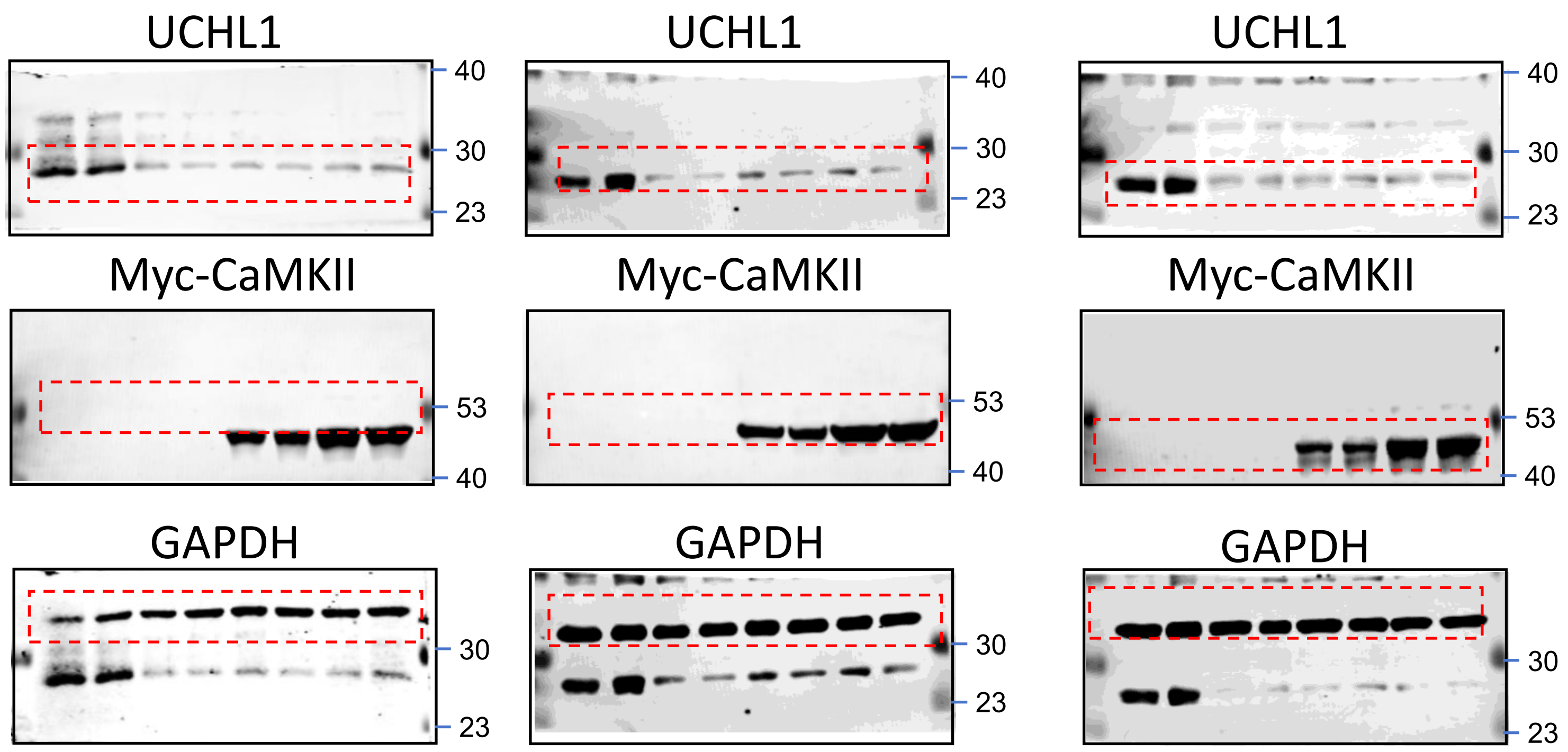

Full unedited gel for Figure S10I

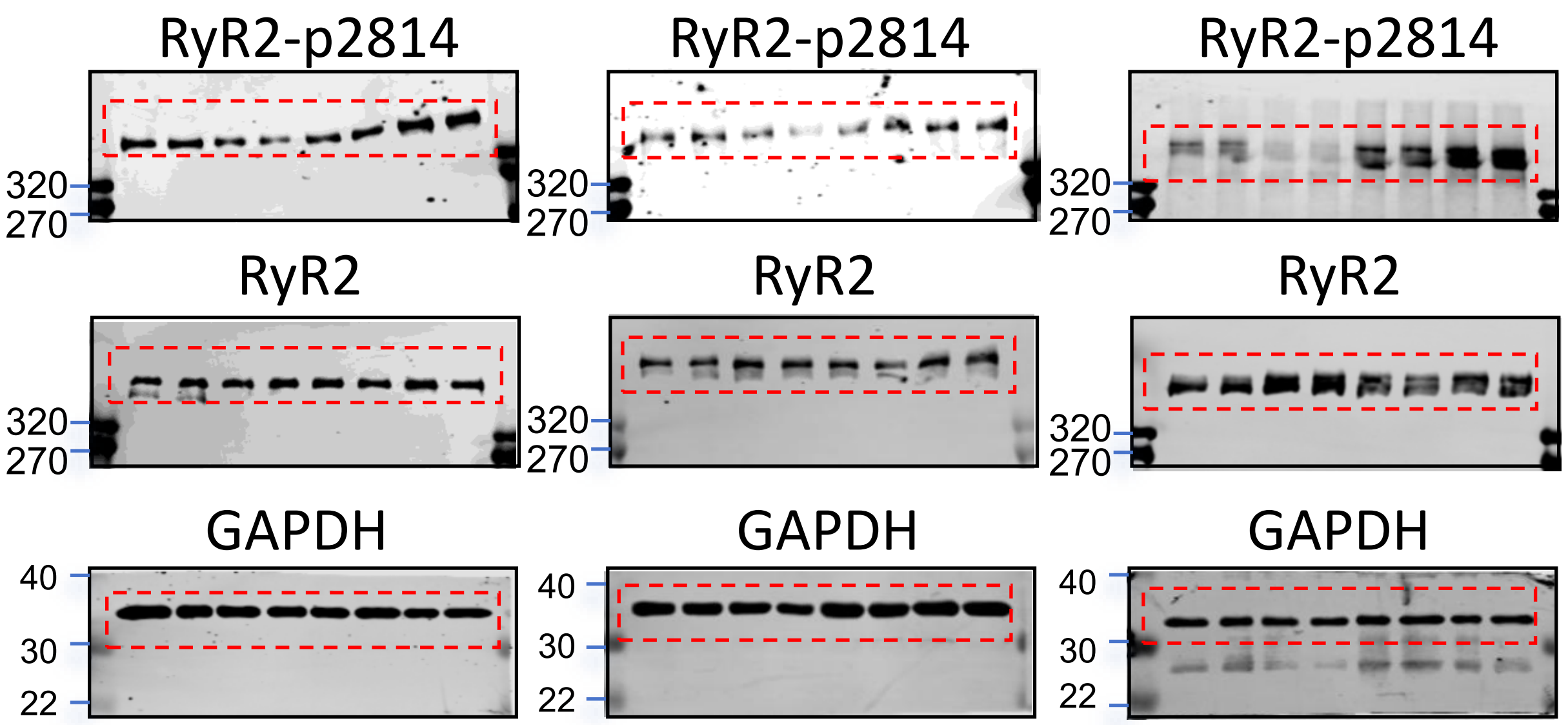

Full unedited gel for Figure S13B

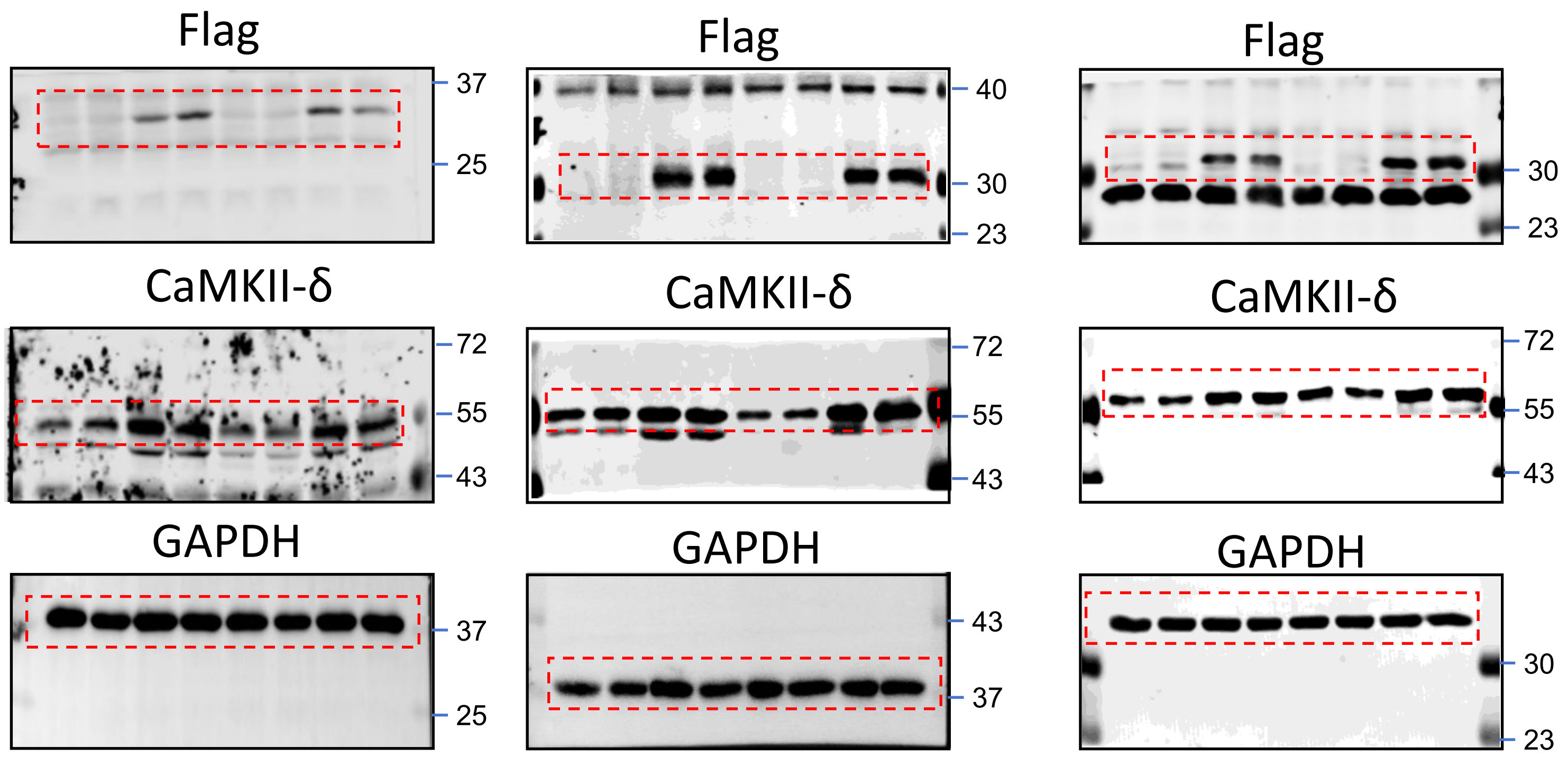

Supplement: Supplementary file 1 [file ADVS-9999-e77855-s001.pdf]
