# Supplementary material for: The Deubiquitinase UCHL1 Drives Susceptibility to Atrial Fibrillation by Stabilizing CaMKII‐δ
Source: Adv Sci (Weinh). 2026 Sep 29:e77855. Online ahead of print. doi: 10.1002/advs.77855 (PMC13624307; doi:10.1002/advs.77855)
Supplement: Supplementary file 2 [file ADVS-9999-e77855-s002.docx]

Supporting Information

Title: The Deubiquitinase UCHL1 Drives Susceptibility to Atrial Fibrillation by Stabilizing CaMKII-δ

Hai-Lian Bi^1,^*, Yu-Hui Gu^1^, Kai-Wen Ren^1^, Xiao-Hong Yu^2^, Yun-Long Xia^1,2^, Xiao-Lei Yang^1,2,^*, and Hui-Hua Li^1,3,^*

H. L. Bi, Y. H. Gu, K. W. Ren, Y. L. Xia, X. L. Yang, H. H. Li

Institute of Cardiovascular Diseases, First Affiliated Hospital of Dalian Medical University, Dalian, China

X. H. Yu, Y. L. Xia, X. L. Yang

Department of Cardiology, First Affiliated Hospital of Dalian Medical University, Dalian, China

H. H. Li

Institute of Biological Therapy, Henan Engineering Research Center for Biotherapy of Metabolic diseases, The 7th People's Hospital of Zhengzhou, Henan Academy of Innovations in Medical Science, Zhengzhou, China.

***Correspondence**: Hai-Lian Bi ([diana_521@163.com](mailto:diana_521@163.com)); Hui-Hua Li ([hhli1935@aliyun.com](mailto:hhli1935@aliyun.com)); Xiao-Lei Yang ([yangxl1012@yeah.net](mailto:yangxl_1012@yeah.net)).

Hai-Lian Bi and Yu-Hui Gu contributed equally to this work.

**Supplementary Materials and Methods**

**Animals and Treatments**

*UCHL1^flox/flox^* ( *UCHL1^fl/fl^* ) mice with LoxP sites flanking canonical exon 4 (see **Figure S3A**) were generated using Cyagen Biosciences Inc. (Guangzhou, China). Atrial-specific Cre recombinase-expressing mice (Nppa-Cre) were obtained from the same centre. *UCHL1^fl/fl^* mice were bred with Nppa-Cre mice to generate experimental cohorts including inducible atrial-specific UCHL1 homozygous knockout mice (UCHL1_cKO_*, Cre^+^/UCHL1^fl/fl^*) and wild-type (WT_KO_, *Cre^–^*/*UCHL1^fl/fl^*) littermates. C57BL/6J WT and *Uchl1^gad-2J^* (*UCHL1^+/-^*) mice (purchased from Jackson Laboratory; Bar Harbor, ME, USA) were used as described previously ^[1]^. The number of mice or atrial myocytes used for each experimental group is presented in the figures or figure legends.

Mice were housed under standard temperature (24°C) and humidity (40–60%), with a 12-hour light/dark cycle in an SPF environment. The mouse feeding and experimental procedures received approval from the Ethics Examination and Approval Committee of the Animal Experimental Center of The First Affiliated Hospital of Dalian Medical University. The wild-type C57B/6J male mice were purchased from Speaform (Beijing) Biotechnology Co., Ltd. for drug administration or rAAV9 injection.

All experiments were approved by the Animal Care and Use Committee of Dalian Medical University and performed in accordance with the US National Institutes of Health Guide for the Care and Use of Laboratory Animals (publication no. 85-23, 1996).

For the Ang II-induced AF model, WT mice (C57BL/6J, 8–10-week-old male) were injected with Ang II (2000 ng/kg/min; sigma) or saline via osmotic mini-pumps (Alzet MODEL 1004; USA) for three weeks to induce atrial fibrillation, as in our previous research ^[2]^. The blood pressure (BP) was measured and recorded once every 3 days. A BP reading of > 150 mmHg and maintained for three weeks indicated that the AF model was successfully constructed. The mice were sacrificed to collect serum and heart tissue samples at the end of the experiment.

For drug administration, mice (C57BL/6J, 8-week-old male) were intraperitoneally injected with KN93 (2.67 ug/mg of body weight per mouse) 3 times a week until tissue collection, or 6RK73 (0.5 ug/mg, 3 times a week) for 2 days prior to Ang II infusion.

For rAAV9 injection, the mice (C57BL/6J, 8-week-old male) were injected with the recombinant adeno-associated virus (rAAV9) expressing GFP or UCHL1 (pAAV-cTNT-hUCHL1-3 × Flag-P2A-EGFP vector, rAAV9-UCHL1; ViGene Biosciences, Shandong, China), UCHL1-C90S (pAAV-cTNT-hUCHL1-C90S- 3 × FLAG-P2A-EGFP, rAAV9-UCHL1-C90S; Cyagen Biosciences Inc), CaMKII-δ (pAAV-NPPA-Myc-hCAMK2D-P2A-EGFP, rAAV9-CaMKII-δ; Cyagen Biosciences Inc), CaMKII-δ-K251R (pAAV-NPPA-Myc-hCAMK2D-K251R-P2A-EGFP, rAAV9-CaMKII-δ-K251R; Cyagen Biosciences Inc), or expressing shRNA targeting CaMKII-δ (rAAV9-cTNT-shRNA-CaMKII-δ-ZsGreen, rAAV9-siCaMKII-δ; 5'-TTGTATACAA CAGATTCTAGAGA -3', HanBio Biotechnology) via the tail vein for 2 weeks ^[3]^, followed by 3 weeks of Ang II infusion. Mice were sacrificed and atrial tissues were collected for further analysis.

**Randomization and Blinding procedures**

All animal experiments were performed and analyzed in a blinded manner ^[4]^. Treatment groups were assigned in a randomized fashion. Each mouse was assigned a temporary random number within the weight range. When mice were randomly divided in each group, they were given their permanent numerical designation in the cages. For each group, a cage was selected randomly from the pool of all cages. AF induction via burst pacing, echocardiography, histological quantification, and western blot analysis were performed by two investigators blinded to group assignment and treatment. The data analyses were also conducted by the same blinded investigators. Unblinding occurred only after all data analyses were completed.

For all *in vivo* studies, we used male mice only. As studies have shown that estrogen, as well as low testosterone levels, may be protective against cardiac dysfunction ^[5]^, we performed all mechanistic studies in male mice that generates robust atrial remodeling and atrial fibrillation upon Ang II challenge. Mice that died during Ang II infusion, failed to complete the infusion protocol, or exhibited atrioventricular block during burst pacing were excluded from final analysis. No animals were excluded without documented justification.

**Echocardiography in Mice**

Transthoracic echocardiography was performed on mice using a Vevo 1100 ultrasound system with a 30 MHz transducer (VisualSonics, Canada), following previously described procedures.^4^ Mice were anaesthetised with 1% isoflurane (Cat# 792632, Sigma-Aldrich, USA) delivered at a constant flow rate of 1.0 L/min through a precision vaporiser connected to a nose cone, maintaining a light plane of anaesthesia that preserved spontaneous respiration and stable haemodynamic parameters. Prior to imaging, the thoracic fur was carefully removed with depilatory cream and wiped with sterile saline to reduce acoustic impedance. Mice were placed supine on a thermostatically controlled heating pad (36–40°C) to maintain body temperature, with limbs secured to ECG electrodes for continuous heart rate monitoring.

Two-dimensional (2D)-guided M-mode echocardiographic tracings were acquired from the parasternal short-axis view at the papillary muscle level for left ventricular (LV) assessment, and from the parasternal long-axis view at the aortic root level for left atrial diameter (LAD) measurement. Imaging parameters were standardised as follows: depth set at 2 cm to ensure full cardiac chamber visualisation, and sweep speed fixed at 100 mm/s to accurately capture systolic and diastolic events. For each measurement, at least three consecutive cardiac cycles were recorded to ensure consistency. The following parameters were analysed offline using the system's dedicated software (Vevo LAB 3.1.0, VisualSonics): end-diastolic left atrial diameter (LAD); LV end-diastolic and end-systolic dimensions; end-diastolic and end-systolic thicknesses of the LV anterior and posterior walls. All measurements were performed by a single investigator blinded to the experimental groups, with intra-observer variability confirmed to be <5% through repeated analyses of 10 randomly selected samples.

**High-resolution Optical Mapping**

Mice were injected intraperitoneally with 0.2 mL heparin (1000 IU/mL) and euthanised 15 min later. Hearts were flushed with K-H buffer containing heparin, mounted on a perfusion needle, and transferred to a Langendorff apparatus (37°C; flow rate 2–2.5 mL/min). K-H buffer contained (mM): 119 NaCl, 4 KCl, 1.8 CaCl_2_·2H_2_O, 1 MgCl_2_·6H_2_O, 1.2 NaH_2_PO_4_, 25 NaHCO_3_, and 10 D-Glucose (pH 7.4) and was bubbled with 95% O_2_/5% CO_2_. The perfusate contained 6.84 µM Blebbistatin (ab120425, Abcam, UK), a substance that uncouples excitation and contraction, to avoid motion artifacts during optical recording ^[6]^. The calcium-sensitive dye was made more hydrophilic by adding 20 µL of 20% Pluronic F127 (P3000MP, Invitrogen, USA) for 15 minutes. Rhod-2 AM (40 µL of 1 mg/mL in DMSO, final concentration 0.71µM) was added for 30 minutes. Electrocardiographic electrodes were used to monitor cardiac rhythm.

Excitation light was provided by two LED sources at 530±25 nm and bandpass filtered (511–551 nm). (LEDC-2001, MappingLab, United Kingdom). An objective was used to gather the atria's emission light, and intracellular Ca^2+^ signals were isolated with a 590 nm bandpass filter (32 nm bandwidth). Digital images (150×150 pixels) were acquired simultaneously using a high-speed CMOS cameras (OMS-PCI-2002, MappingLab, UK) at a frame rate of 0.9 kHz. OMapScope software (v5.9.45) was used to record and analyse several parameters, including the conduction velocity of calcium transients, time to peak of calcium transients, calcium transient duration at 90% recovery (CaD_90_), and calcium transient amplitude.

**Adult Mouse Cardiomyocyte Isolation**

Cardiomyocytes were isolated from adult mice using a Langendorff retrograde perfusion system with an optimized enzymatic digestion protocol. Mice received an intraperitoneal injection of sodium heparin (100 U/20 g body weight); after 15 min of heparinization, they were anesthetized with tribromoethanol (150 mg/kg, i.p.). The heart was rapidly excised and placed in ice-cold Ca^2+^-free Tyrode’s solution (135 mM NaCl, 5.4 mM KCl, 10 mM HEPES, 10 mM D-Glucose, 1 mM MgCl_2_·6H_2_O, 0.33 mM NaH_2_PO_4_, 10 mM Taurine, pH 7.4 adjusted with NaOH). The ascending aorta was cannulated and retrogradely perfused with oxygenated Ca^2+^-free Tyrode's solution at 37°C for 5 minutes, followed by switching to an enzymatic digestion solution containing digestive enzymes such as 0.5 mg/ml Collagenase II, 1 mg/ml bovine serum albumin (BSA), and 0.1 mM CaCl_2_. After enzymatic digestion, the heart was transferred to Tyrode's solution containing 2% BSA. Cut the tissue into small pieces of 1 mm³, gently pipette the pieces to dislodge the cardiomyocytes, and filter the cell suspension through a 200-mesh sieve. Ca^2+^ was reintroduced stepwise to the final desired concentration of 1.8 mM, and the cardiomyocytes were allowed to sediment by gravity to obtain a high-yield rod-shaped cell preparation.

**Intracellular Ca^2+^ Transient and Ca^2+^ Spark Recording**

For Ca^2+^ transient and Ca^2+^ spark measurements, freshly isolated adult cardiomyocytes were loaded with 2 uM Fluo-4 AM (S1060, Beyotime, China) supplemented with 0.05% Pluronic F-127 (P3000MP, Invitrogen, USA) in standard Tyrode's solution for 30 min at room temperature. In order to fully de-esterify intracellular Fluo-4 AM, the cells were twice rinsed with dye-free Tyrode's solution and then incubated for a further 20 minutes at room temperature. Fluo-4 AM-loaded cardiomyocytes were transferred to a 1.5 mL experimental chamber mounted on the stage of an inverted confocal microscope. All Ca^2+^ imaging recordings were performed using a Leica STELLARIS 5 confocal system (Leica Microsystems, Germany) operated by LAS X acquisition software, using an 63 × oil objective. Ca^2+^ transients were evoked by field stimulation at 1 Hz through platinum electrodes and recorded in line-scan mode after steady-state transients were achieved. Spontaneous Ca^2+^ sparks were recorded in line-scan mode in quiescent cardiomyocytes. Ca^2+^ spark frequency was calculated as the number of sparks per 100 µm of scan distance per second (sparks per 100 µm/s). Ca^2+^ transient amplitude was expressed as ΔF/F0, where ΔF = F − F0, F is the peak fluorescence intensity of Ca^2+^ transient and F0 is the baseline fluorescence at resting cytosolic Ca^2+^ levels. Ca^2+^ sparks were analyzed using SparkMaster, an automated ImageJ plugin.

**Statistical Analysis**

All values are reported as mean ± standard deviation (SD) of independent experiments. Sample numbers are shown in the figures and figure legends or in the Methods. Statistical analysis was carried out using Prism 9.0 (GraphPad) and Excel (Microsoft 2020). First, the data were tested for normality using the Shapiro–Wilk normality test. If each group satisfied the normality (*P* value in Shapiro–Wilk normality test was >0.05), we used an unpaired student’s t-test for two-group-only comparisons, and one-way (one variable) or two-way (two variable) ANOVA followed by the Bonferroni post hoc test for comparisons involving three or more groups. Otherwise, the nonparametric Mann–Whitney U test was employed. Pearson Chi-Square/Fisher's exact test was used to test atrial fibrillation (AF) inducibility. Differences were considered statistically significant when the *P* value was < 0.05.

**References**

[1]	L. H. Bi H Zhang X, Zhang Y, Xie X, Xia Y, Du J., *Science Advances* **2020**.

[2]	L. H. Bi H Zhang Y, Yang J, Shu Q, Yang X, Yan X, Chen C, Li Z., *Hypertension Research* **2020**, 43, 168.

[3]	D. Zhao, G. Zhong, J. Li, J. Pan, Y. Zhao, H. Song, W. Sun, X. Jin, Y. Li, R. Du, J. Nie, T. Liu, J. Zheng, Y. Jia, Z. Liu, W. Liu, X. Yuan, Z. Liu, J. Song, G. Kan, Y. Li, C. Liu, X. Gao, W. Xing, Y.-Z. Chang, Y. Li, S. Ling, Circulation **2021**, 144, 694.

[4]	J. Chen, J. Lai, L. Yang, G. Ruan, S. Chaugai, Q. Ning, C. Chen, D. W. Wang, *Br* *J Pharmacol* **2016**, 173, 545.

[5]	P. Ortiz-Sánchez, M. Villalba-Orero, M. M. López-Olañeta, J. Larrasa-Alonso, F. Sánchez-Cabo, C. Martí-Gómez, E. Camafeita, J. M. Gómez-Salinero, L. Ramos-Hernández, P. J. Nielsen, J. Vázquez, M. Müller-McNicoll, P. García-Pavía, E. Lara-Pezzi, *Circulation Research* **2019**, 125, 170.

[6]	J. Liao, S. Zhang, S. Yang, Y. Lu, K. Lu, Y. Wu, Q. Wu, N. Zhao, Q. Dong, L. Chen, Y. Du, *Front Immunol* **2021**, 12, 758157.

**Supplemental Figure S1**

**Supplementary Figure S1. UCHL1 expression in atrial tissues, primary atrial cardiac fibroblasts, and patient cohort flow chart.**

(A) Immunofluorescence staining of UCHL1 and Troponin T (cTNT, a cardiac marker) in atrial tissues from mice treated with saline or Ang II infusion (n = 6 mice/group). Scale bars: 20 μm. (B) Representative immunoblot (left) and quantification (right) of UCHL1 protein levels in primary ACFs treated with Ang II (100 nM) for 12 or 24 h. GAPDH was used as an internal control (n = 4 independent experiments). (C) Flowchart of patient enrollment and cohort allocation. After applying pre-defined exclusion criteria (NYHA class ≥ III heart failure, severe heart disease, endocrine disorders, cancer, renal dysfunction: eGFR < 30 mL/min/1.73 m², stroke history, infection, recent surgery/trauma, and incomplete clinical or laboratory data ), 50 SR controls and 82 AF patients were included in the final serum cohort for ELISA. Atrial tissues were collected from 7 AF patients and 7 SR controls for microarray, qPCR, immunohistochemistry (IHC), and immunoblotting analyses. Data in **B** are presented as the mean ± SD. *P* values were analyzed using one way ANOVA followed by the Bonferroni post hoc test. ns: not significant.

**Supplementary Figure S2**

**Supplementary Figure S2. UCHL1 Knockdown inhibits Ang II-Induced Atrial Fibrillation.**

(A) Protocol for mouse model of AF. WT and UCHL1-knockdown (KD) mice received Ang II (2000 ng/kg/min) or saline for 3 weeks, followed by ECHO and electrical stimulation. (B) Immunoblots (left) and quantification (right) of UCHL1 protein levels in atria (n = 6 mice per group). GAPDH as an internal control. (C) Intracardiac electrogram (EGM) recordings of each group. Red dotted lines represent atrial fibrillation (AF), and green solid lines represent sinus rhythm (SR). (D) Percentage of successful AF induction (left, n = 20 mice per group) and the duration of AF (right). (E) Representative images of M-mode echocardiography (ECHO, left) and quantification (right) of LA diameter (LAD, mm) (n = 6 mice per group). (F) Masson’s staining images (left) and quantitation (right) of atrial fibrosis (n = 6 mice per group). Scale bar=20 μm. (G) Immunohistochemical (IHC) staining of LA sections with an anti-CD68 antibody (left) and quantification of CD68-positive cells (right, n = 6 mice per group). Scale bar=20 μm. (H) DHE staining and fluorescence intensity (n = 6 mice per group). Scale bar=20 μm. (I) qPCR analysis of the mRNA expression of *COL1A1, COL3A1, IL-1β,* and *CYBB* (NOX2) in atrial tissues (n = 6 mice per group). The results are presented as mean ± SD. Data presented in B, E, F, G, H, and I were analyzed using two-way ANOVA followed by the Bonferroni post hoc test. Data in D were analysed using Fisher’s exact test for fraction of successful AF induction, and Mann–Whitney U tests followed by Dunn's multiple comparison test for duration of AF.

**Supplementary Figure S3**

**Supplementary Figure S3. Construction of atrial cardiomyocyte-specific UCHL1 knock out mice.**

(A) Schematic of UCHL1 conditional knockout (UCHL1-CKO) via NPPA-Cre-mediated excision of floxed *UCHL1* exon 4 (atrial cardiomyocyte-specific). (B) Representative PCR genotype image of UCHL1 ^flox/flox^ (226 bp) and primers used (n = 6 mice per group). (C) Representative PCR genotype image of NPPA Cre (363 bp) and primers used (n = 6 mice per group). (D) Representative immunoblot (top) and quantification (bottom) of UCHL1 protein levels in ventricular myocardium and skeletal muscle from ACM-specific UCHL1-knockout mice (*UCHL1^fl/fl^ / NPPA-Cre^+^*, UCHL1-CKO; male, 8-week-old) and age-matched littermates (*UCHL1^fl/fl^*, CTR, male; n = 6 mice per group). GAPDH as an internal control. (E) Average systolic blood pressure of each group before and after angiotensin II treatment (n = 6 mice per group). Data are presented as mean ± SD. Data were analyzed using two-way ANOVA followed by the Bonferroni post hoc test. ***P*<0.01 versus CTR+Saline to CTR+Ang II.

**Supplementa****ry Figure S4**

**Supplementary Figure S4. UCHL1 overexpression does not influence the Ang II-induced augmentation of SBP in Mice.**

Average systolic blood pressure of each group before and after angiotensin II treatment (n = 6 mice per group). The results are presented as mean ± SD. Data were analyzed using two-way ANOVA followed by the Bonferroni post hoc test. ***P*<0.01 versus rAAV9-EV+Saline to rAAV9-EV+Ang II.

**Supplementary Figure S5**

**Supplementary Figure S5. Lysine ubiquitinome profile in UCHL1 overexpressed ACMs**

(A) Heatmap indicating the enrichment (red) and depletion (green) of amino acids at each position flanking the Ub sites. (B) The motif sequences within UCHL1-interacting proteins. (C) The differentially-ubiquitinated proteins (DUPs) were classified into four clusters by the Q classification method for further enrichment analysis. KEGG analysis for DUPs in UCHL1-overexpressed ACMs.

**Supplementary Figure S6**

**Supplementary Figure S6. UCHL1 specifically cleaves K48-linked polyubiquitin chains from CaMKII-δ.**

(A and B) Primary atrial cardiomyocytes (ACMs) were infected with Ad-EV or Ad-UCHL1 for 24 h, treated with MG132 (10 μM) for 6 h, and subjected to immunoprecipitation with anti-CaMKII-δ antibody, followed by immunoblotting with K48-specific ubiquitin antibody (A) or K63-specific ubiquitin antibody (B).

**Supplementary Figure S7**

**Supplementary Figure S7. UCHL1 prolongs CaMKII-δ half-life via K251 deubiquitination.**

(A and B) ACMs were infected with Ad-CaMKII-δ-WT or Ad-CaMKII-δ-K251R together with Ad-EV or Ad-UCHL1, treated with CHX (10 μM), and harvested at 0 h, 24 h, and 48 h. CaMKII-δ protein levels were detected by immunoblotting (A) and quantified (B, n = 3 independent experiments). Data are presented as mean ± SD. Statistical analysis was performed using two-way ANOVA followed by the Bonferroni post hoc test. ***P*<0.01 versus Ad-K251R+Ad-EV to Ad-WT+Ad-EV.

**Supplementary Figure S8**

**Supplementary Figure S8. UCHL1 overexpression does not affect CaMKII-δ transcriptional level.**

(A) qPCR analysis of the mRNA expression of *CaMKII-δ* in cardiomyocytes infected with Ad-GFP or Ad-UCHL1 (n = 6 mice per group). (B) qPCR analysis of the mRNA expression of *CaMKII-δ* in cardiomyocytes infected with Ad-siRNA-control or Ad-siRNA-UCHL1 (n = 6 mice per group). The results are presented as mean ± SD. Data were analyzed by Mann-Whitney U tests. “ns” indicates not significant.

**Supplementary Figure S9**

**Supplementary Figure S9. The deubiquitinase activity of UCHL1 is required for its pro-arrhythmic effect *in vivo*.**

(A) Protocol for rAAV9 injection and AF mouse model generation. UCHL1-CKO or *UCHL1^fl/fl^* (CTR) mice were injected with rAAV9-EV, rAAV9-UCHL1-WT (UCHL1^OE^) and rAAV9-UCHL1-C90S (catalytically inactive mutant; UCHL1-C90S^OE^; under the cardiac-specific cTNT promoter) for 2 weeks, followed by Ang II infusion for 3 weeks. (B) Representative immunoblots (left) and quantification (right) of Flag-tagged UCHL1 (WT or C90S) and endogenous CaMKII-δ protein levels in atrial tissues from the indicated groups (n = 6 mice per group). GAPDH as internal control. (C) Representative intracardiac electrogram recordings following burst pacing in each group. Red dotted lines represent AF, and green solid lines represent sinus rhythm (SR). (D) Percentage of successful AF induction (upper) and total duration of AF (lower, n = 20 mice per group). (E-F) Representative M-mode echocardiography images (left) and quantification of left atrial diameter (LAD, right) in each group (n = 6 mice per group). (G-H) Representative images of Masson’s staining (left) and quantification (right) of atrial fibrotic area (n = 6 mice per group). Scale bar: 20 μm. Data are presented as mean ± SD. AF inducibility was analyzed using chi-square test (χ² test) with Bonferroni correction for multiple comparisons; AF duration was analyzed using Mann-Whitney U test followed by Dunn's multiple comparison test; data in (B), (F) and (H) were analyzed using two-way ANOVA followed by the Bonferroni post hoc test.

**Supplementary Figure S10**

**Supplementary Figure S10. The K251 ubiquitination site in CaMKII*-*δ is critical for UCHL1-mediated CaMKII-δ stabilization and pro-arrhythmic effects *in vivo*.**

(A) Experimental protocol. UCHL1-CKO mice were injected with rAAV9-EV, rAAV9-CaMKII*-*δ-WT, or rAAV9-CaMKII*-*δ-K251R (under the atrial cardiomyocyte-specific NPPA promoter), followed by Ang II infusion for 3 weeks. (B) Representative immunoblots (upper) and quantification (lower) of UCHL1 and total CaMKII*-*δ protein levels in atrial tissues from the indicated groups (n = 6 mice per group). GAPDH served as loading control. (C) Representative intracardiac atrial electrogram (A-EGM) recordings following burst pacing. Red dotted lines indicate AF; green solid lines indicate SR. (D) Percentage of successful AF induction (left) and total AF duration (right) (n = 20 mice per group). (E-F) Representative M-mode echocardiography images and quantification of left atrial diameter (LAD) (n = 6 mice per group). (G-H) Representative Masson’s staining images and quantification of atrial fibrotic area (n = 6 mice per group). Scale bar: 20 μm. (I-J) Representative immunoblots and quantification of p-RyR2-S2814 protein levels in atrial tissues (n = 6 mice per group). Data are presented as mean ± SD. AF inducibility was analyzed using Fisher's exact test; AF duration was analyzed using Mann-Whitney U test followed by Dunn's multiple comparison test; other data were analyzed using two-way ANOVA followed by the Bonferroni post hoc test.

**Supplementary Figure S11**

**Supplementary Figure S11. UCHL1 promotes atrial calcium handling under Ang II stress.**

(A) Quantification of time to peak at 8 Hz pacing cycle length (PCL) of 125ms in the atria from CTR and UCHL1-CKO mice after saline or Ang II infusion (n = 6 mice per group). (B) Calculation of the calcium transient alternans ratios (CaT alternans ratios, top). Statistical plots of the mean values of the calcium transient alternans ratios for 4 groups at different cycle lengths (bottom, n = 6 mice per group). (C) Representative waveforms of calcium transient alterations at various pacing cycle lengths (150 ms, 120 ms, and 90 ms) in the four groups induced by a gradual decrease in the length of the pacing cycle through electrical stimulation. (D) Quantification of time to peak at 8 Hz pacing cycle length (PCL) of 125ms in the atria of rAAV9-EV and rAAV9-UCHL1-injected mice after saline or Ang II infusion (n = 6 mice per group). (E) Calculation of the calcium transient alternans ratios (CaT alternans ratios, top). Statistical plots of the mean values of the calcium transient alternans ratios for 4 groups at different cycle lengths (bottom, n = 6 mice per group). (F) Representative waveforms of calcium transient alterations at various pacing cycle lengths (150 ms, 120 ms, and 90 ms) in the four groups induced by a gradual decrease in the length of the pacing cycle through electrical stimulation. The results are presented as mean ± SD. Data were analyzed using two-way ANOVA followed by the Bonferroni post hoc test. **P* < 0.05, ***P* < 0.01 versus CTR + saline; ^#^*P* < 0.05; ^##^*P* < 0.01 versus CTR + Ang II; n = 6 mice per group.

**Supplementary Figure S12**

**Supplementary Figure S12. UCHL1 overexpression enhances cardiomyocyte Ca**^2+^ **transient and spark properties via CaMKII-δ.**

(A-B) Representative confocal line-scan images and quantification of spontaneous Ca^2+^ sparks in cardiomyocytes from rAAV9-UCHL1- and rAAV9-siCaMKII-δ-injected mice under Ang II exposure (n=12 cells from 4 mice per group). (C-D) Representative line-scan images and quantification of Ca^2+^ transients (ΔF/F_0_) in cardiomyocytes during 1 Hz pacing under Ang II infusion (n=12 cells from 4 mice per group). (E-F) Representative normalized Ca^2+^ transient traces and statistical analysis of decay time constant (Tau) (n=12 cells from 4 mice per group). Data are presented as mean ± SD. Data were analyzed using two-way ANOVA followed by the Bonferroni post hoc test.

**Supplementary Figure S13**

**Supplementary Figure S13. The pro-arrhythmic role of UCHL1 on Ang II-induced AF** **was attenuated by the administration of CaMKII-δ inhibitor KN93.**

(A) Protocol for rAAV9 injection, KN93 administration, and mouse model of AF. WT mice were injected with rAAV9-EV or rAAV9-UCHL1 for 2 weeks, and then administrated with KN93 or DMSO and treated with Ang II for additional 3 weeks. (B) Representative immunoblots (left) and quantification (right) of UCHL1 and CaMKII protein levels using anti-Flag or anti-CaMKII-δ antibodies (n = 6 mice per group). (C) Intracardiac electrogram (EGM) recordings as in (A) and subjected to burst pacing. Red dotted lines represent AF, and green solid lines represent SR. **(**D) Percentage of successful AF induction (left, n = 20 mice per group) and the total duration of AF (right). (E) Representative images of M-mode ECHO of LAD (mm) from rAAV9-UCHL1-injected or KN93-administrated mice infused with Ang II, as indicated (n = 6 mice per group). (F) Masson’s staining of left atrial tissue (left) and quantification of fibrotic area (right, n = 6 mice per group). (G) Representative images of DHE staining in atrial tissues from rAAV9-UCHL1-injected or KN93-administrated mice infused with Ang II (n = 6 mice per group). Scale bar: 20 μm. (H) qPCR analysis of the mRNA expression of *COL1A1, COL3A1, CYBB* (NOX2)*, NOX4,* and *IL-1β* in atrial tissues (n = 6 mice per group). The results are presented as mean ± SD. Data in B, E, F, G, and H presented were analyzed using two-way ANOVA followed by the Bonferroni post hoc test.

**Supplementary Figure S14**

**Supplementary Figure S14. Administration of the UCHL1 inhibitor 6RK73 did not induce overt cardiac dysfunction and injury in Ang II-infused mice.**

WT (C57BL/6J, 8-week-old male) mice were treated with 6RK73 (0.5 mg/kg, three times per week) for 2 days, and then infused with Ang II (2000 ng/kg/min ) and 6RK73 for 3 weeks. (A) Measurement of left ventricular ejection fraction (EF%) in 6RK73- or DMSO-treated mice infused with or without Ang II (n = 6 mice per group). (B) LDH activity in mice as in (A) (n = 6 mice per group). The results are presented as mean ± SD. Data were analyzed using two-way ANOVA followed by the Bonferroni post hoc test.

**Supplementary Figure S15**

**Supplementary Figure S15. Proposed mechanism of UCHL1 as a driver of AF under hypertension induced by Ang II.**

Atrial myocytes Loss of UCHL1 represses stabilization of CaMKII-δ and its down-regulator RyR2 phosphorylation (2814), which leads to Ca²⁺ mishandling, atrial remodeling and AF.

Ang II, angiotensin II; AT1R, angiotensin II type 1 receptor; UCHL1, ubiquitin carboxyl-terminal hydrolase L1; CaMKII-δ, calcium/calmodulin-dependent protein kinase II delta; RyR2, ryanodine receptor 2; AF, atrial fibrillation.

**Supplementary Tables**

**Supplementary Table S1. Clinical characteristics of the study participants for atrial biopsies**

| Variable | SR (n=7) | AF (n=7) | *p-value* |
| --- | --- | --- | --- |
| Sex (male/female) | 4/3 | 4/3 |  |
| BMI, kg/m^2^ | 25.40±5.87 | 24.59±3.47 | 0.7929 |
| Age (years) | 66.14±11.35 | 67.43±4.65 | 0.7862 |
| Paroxysmal AF | No | No |  |
| Persistent/permanent AF | No | Yes^#^ |  |
| Unclassified AF | No | No |  |
| Ejection Fraction (%) | 56.00±4.47 | 49.43±7.39 | 0.1268 |
| Left atrial diameter (mm) | 39.43±5.41 | 60.14±13.22 | 0.0050*** |
| Left ventricle end-diastolic diameter (mm) | 54.71±10.42 | 56.00±8.72 | 0.676 |
| Medication, n | | | |
| Diuretics | 0 | 2 |  |
| β-Blockers | 1 | 2 |  |
| Ca^2+^ antagonists | 0 | 0 |  |
| Amiodarone | 0 | 0 |  |
| Warfarin | 0 | 3 |  |

Values are means ± SD. ** p<0.05*. AF patients received radiofrequency ablation procedures: 3 for PV segmental isolation, 1 for wide area circumferential ablation and 3 for PV Antrum ablation.

**Supplementary Table S2. Clinical characteristics of the study participants for serum samples**

| Parameters | SR  （n=50） | AF  （n=82） | *P* value |
| --- | --- | --- | --- |
| Demographics | | | |
| Male, n (%) | 15(30.0%) | 55(67.1%) |  |
| Age, y | 49.84±12.98 | 65.87±7.17 | <0.001 |
| Hemodynamic variables | | | |
| Resting heart rate, beats/min | 71.3±10.1 | 76.4±15.0 | 0.021 |
| SBP, mmHg | 123.4±10.2 | 134.2±18.8 | <0.001 |
| DBP, mmHg | 76.2±7.7 | 85.4±12.9 | <0.001 |
| Echocardiography | | | |
| LVEF, % | 58.8±1.3 | 57.5±2.2 | <0.001 |
| LA diameter, mm | 33.8±3.2 | 40.1±5.0 | <0.001 |
| LV diameter, mm | 45.6±3.3 | 46.8±4.0 | 0.070 |
| Blood-based biomarkers | | | |
| UCHL1, ng/ml | 1.817±3.338 | 34.337±75.053 | <0.001 |
| FBG, mmol/L | 4.6±0.6 | 5.2±0.9 | <0.001 |
| AST, umol/L | 18.5±5.4 | 20.2±6.0 | 0.097 |
| SCr, umol/L | 61.5±12.8 | 74.9±16.7 | <0.001 |
| TC, mmol/L | 1.4±0.9 | 1.4±0.8 | 0.786 |
| LDL, mmol/L | 2.4±0.6 | 2.3±0.8 | 0.750 |
| HDL, mmol/L | 1.3±0.3 | 1.3±0.3 | 0.109 |
| BNP, pg/ml | 29.418±21.780 | 138.956±102.660 | <0.001 |

**P<0.05,* ***P<0.01* vs SR control; SR, sinus rhythm; AF, atrial fibrillation; SBP, systolic blood pressure; DBP, diastolic blood pressure; LA, left atrial; LVEF, left ventricular ejection fraction; FBG, fasting blood glucose; AST, aspartate transaminase; SCr, serum creatinine; TC, Total cholesterol; and LDL, low-density lipoprotein; HDL, high-density lipoprotein; NA, not applicable; and. BNP indicates B-type natriuretic peptide. The parameters are mean (SD) or n (%).

**Supplementary Table S3. Multiple logistic regression analysis of UCHL1 associated with AF**

| UCHL1, per U increment | OR (95%*CI*) | *P* value |
| --- | --- | --- |
| Model 1 | 1.222(1.023, 1.459) | 0.027 |
| Model 2 | 1.280 (1.054, 1.553) | 0.013 |
| Model 3 | 1.293 (1.043, 1.602) | 0.019 |

Model 1 was unadjusted, Model 2 was adjusted for sex and age, and Model 3 was adjusted for all potential confounders including sex, age, SBP, SCr, BNP, LA diameter, and HDL cholesterol.

**Supplementary Table S4. RT-qPCR Primer Information**

| **Gene** | **Forward Primer (5’-3’)** | **Reverse Primer (5’-3’)** |
| --- | --- | --- |
| ***Human*** | | |
| *UCHL1* | CTTCATGAAGCAGACCATTG | ATCATGGGCTGCCTGTATG |
| *GAPDH* | GGAGCGAGATCCCTCCAAAAT | GGCTGTTGTCATACTTCTCATGG |
| ***Mouse*** | | |
| *UCHL1* | CGGCCCAGCATGAAAACTTC | TTATTGGCCACTGCGTGGAT |
| *CYLD* | GTCTAGGATATTTTGTTGGTGTGG | ACGCTATCTGATAAAGCTGGG |
| *ANP* | CACAGATCTGATGGATTTCAAGA | CCTCATCTTCTACCGGCATC |
| *Collagen I (COL1A1)* | AGTCGATGGCTGCTCCAAAA | AGCACCACCAATGTCCAGAG |
| *Collagen III (COL3A1)* | TCCTGGTGGTCCTGGTACTG | AGGAGAACCACTGTTGCCTG |
| *IL-1β* | TGAAAACACAGAAGTAACGTCCG | CCCAGGAGGAAATTGTAATGGGA |
| *IL-6* | TTCCATCCAGTTGCCTTCTTG | TTGGGAGTGGTATCCTCTGTGA |
| *NOX2 (CYBB)* | CTTCTTGGGTCAGCACTGGC | GCAGCAAGATCAGCATGCAG |
| *NOX4* | CTTGGTGAATGCCCTCAACT | TTCTGGGATCCTCATTCTGG |
| *CaMKIID* | AGGGACCTGAAGCTGAGAA | GTGCCAGCAAAACCAAACCA |
| *GAPDH* | GGTTGTCTCCTGCGACTTCA | GGTGGTCCAGGGTTTCTTACTC |
| ***Rat*** | | |
| *CaMKIID* | AGGGACCTGAAGCTGAGAA | GTGCCAGCAAAACCAAACCA |
| *GAPDH* | CCCCCAATGTATCCGTTGTG | TAGCCCAGGATGCCCTTTAGT |

**Supplementary Table S5. Antibody Information**

| **Target antigen** | **Vendor or Source** | **Catalog #** | **Working concentration** |
| --- | --- | --- | --- |
| UCHL1 | Proteintech | 14730-1-AP | 1:1500 (WB), 1:100 (IF) |
| UCHL1 | Proteintech | 66230-1-Ig | 1:200 (IF) |
| CaMKIID | Proteintech | 20667-1-AP | 1:1000 (WB), 1:50 (IF) |
| Kv1.5 | Proteintech | 21659-1-AP | 1:1000 (WB) |
| NOX2 | Absin | abs124860 | 1:1500(WB) |
| TGF-β | Proteintech | 21898-1-AP | 1:1000 (WB) |
| NLRP3 | Proteintech | 19771-1-AP | 1:1000 (WB) |
| CD68 | Proteintech | 28058-1-AP | 1:100 (IF) |
| RYR2-p2814 | Abclone | AP0624 | 1:800(WB), 1:200 (IF) |
| RYR2 | Proteintech | 19765-1-AP | 1:1500 (WB) |
| Ub | CST | #3936 | 1:1000 (WB) |
| Flag | Proteintech | 20543-1-AP | 1:3000 (WB) |
| Myc | Proteintech | 60003-2-Ig | 1:2000(WB) |
| HA | Proteintech | 51064-2-AP | 1:2000(WB) |
| GFP | Proteintech | 50430-2-AP | 1:2000(WB) |
| cTNT | Proteintech | 68300-1-Ig | 1:200 (IF) |
| α-Actinin | Sigma | A7811 | 1:400 (IF) |
| GAPDH | Huabio | ET1601-4 | 1:5000(WB) |
